# A Highly Selective and Versatile Probe Platform for Visualization of Monoacylglycerol Lipase

**DOI:** 10.1002/anie.202413405

**Published:** 2025-02-07

**Authors:** Axel Hentsch, Mónica Guberman, Silke Radetzki, Sofia Kaushik, Mirjam Huizenga, Jerome Paul, Maria Schippers, Jörg Benz, Bernd Kuhn, Dominik Heer, Andreas Topp, Ludivine Esteves Gloria, Alexander Walter, Remo Hochstrasser, Matthias B. Wittwer, Jens Peter von Kries, Ludovic Collin, Julie Blaising, Mario van der Stelt, Noa Lipstein, Uwe Grether, Marc Nazaré

**Affiliations:** ^1^ Leibniz Forschungsinstitut für Molekulare Pharmakologie (FMP) Robert-Roessle-Strasse 10 13125 Berlin GER; ^2^ Leiden Institute of Chemistry (LIC) Universiteit Leiden Einsteinweg 55 2333 CC Leiden NL; ^3^ Pharma Research & Early Development (pRED) F. Hoffmann-La Roche Ltd Grenzacherstrasse 124 CH-4070 Basel CH

**Keywords:** monoacylglycerol lipase, fluorescent probe, microscopy, click chemistry, activity-based protein profiling

## Abstract

Monoacylglycerol lipase (MAGL) is a key enzyme for signal termination in the endocannabinoid system (ECS). MAGL inhibition results in indirect activation of the cannabinoid receptors, which offers unique advantages for the treatment of, e.g., multiple sclerosis, epilepsy, and other neurological disorders. Molecular imaging techniques are valuable tools to overcome the current poor understanding of MAGL's distribution and role in patho‐ and physiological processes within ECS signaling. Herein, we report the design, synthesis, and validation of highly selective versatile fluorescent and click‐chemistry probes for MAGL. Structure‐based design combined with a reverse‐design approach allowed the development of a structural unit that selectively and effectively recognizes MAGL while offering a versatile platform to attach different fluorophores and further reporter units. In this way, labeled probes with sub‐nanomolar potency carrying diverse fluorescent dyes were obtained. Probe affinity and selectivity remained invariant to changes in the fluorophore subunit, showing the remarkable robustness of this platform in delivering tailor‐made probes. Highly consistent inhibition across species supports pharmacological model translatability. Extensive profiling and validation in various cellular systems shows the ability of these highly potent and selective probes to elucidate the complex role of MAGL in ECS cellular signaling, inflammatory processes, and disease progression.

## Graphical abstract

Monoacylglycerol lipase (MAGL) is a key enzyme in the endocannabinoid system. We developed a highly flexible and specific probe platform to visualize MAGL in several cell types such as neurons or primary human immune cells. These results demonstrate the potential of the probe platform to unravel the manifold (patho)physiological roles and functions of MAGL in the endocannabinoid system.

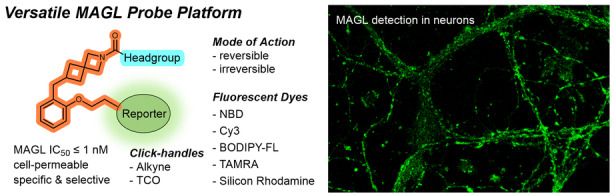

## Introduction

Monoacylglycerol lipase (MAGL) has emerged as a therapeutic target of high interest for treating a multitude of diseases related to inflammation, cancer, and neurodegeneration.[^1^, ^2^, ^3^, ^4^] MAGL is the key enzyme responsible for the hydrolysis of 2‐arachidonoylglycerol (2‐AG), the most abundant endocannabinoid (EC) ligand in the brain.^[5]^ The endocannabinoid system (ECS) is a cell‐signaling network vital for the central nervous system (CNS). It comprises the cannabinoid receptors (CB1 and CB2), endogenous cannabinoid ligands, and degrading enzymes and regulates several physiological and pathological processes, including inflammation, neuroprotection, addiction, and appetite.^[4]^ Targeting MAGL offers unique therapeutic advantages as its inhibition leads to indirect activation of the cannabinoid receptors, thereby avoiding the adverse effects associated with the use of exogenous cannabinoid ligands, such as cognitive and psychological impairment, sedation, and dependency.^[6]^ In addition, MAGL influences the production of pro‐inflammatory prostaglandins, fatty acid metabolism, and lipid signaling that promote pathogenesis in cancer cells. Consequently, the therapeutic interest in MAGL beyond ECS signaling includes treating inflammation, cancer, and metabolic disorders.[^2^, ^7^]

**Figure 1:**
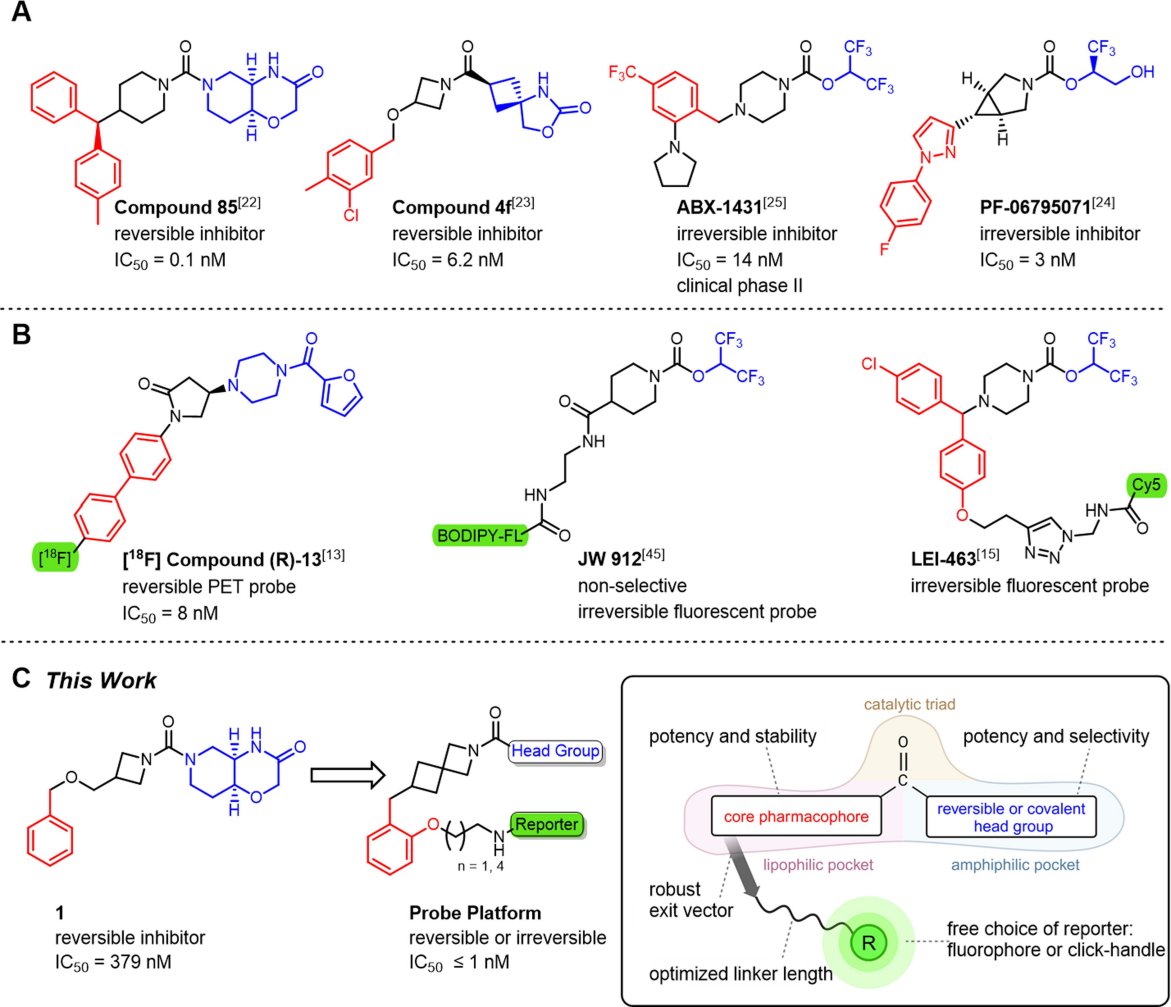
Overview of relevant MAGL inhibitors from drug‐development programs (A), chemical probes for MAGL (B), and the principle design approaches considered in this study (C). Structural elements that bind the amphiphilic pocket (headgroup, blue), the lipophilic pocket (red), or act as reporting group (green) are highlighted. The inset depicts the key probe blueprint and features considered for the construction of the MAGL probe platform attributed to the structural region.

Despite the relevance and remarkable therapeutic potential of MAGL, the understanding of its distribution and role in (patho)physiological processes and EC signaling is scarce.[^8^, ^9^, ^10^] This is partly due to the lack of appropriate imaging tools to characterize MAGL and the ECS. Molecular imaging may greatly expedite therapeutic approaches, as it can provide evidence of biological activity, target expression, and distribution and confirm on‐target drug effects.^[11]^ Although various irreversible and reversible MAGL inhibitors have been reported over the last ten years,[^6^, ^12^] this has poorly translated into imaging probes. MAGL visualization was, up to now, mostly limited to the use of immunohistochemistry, PET tracers,[^13^, ^14^] and one irreversible activity‐based probe, **LEI‐463** ^[15]^ (Figure 1 B). Enzymatic activity of MAGL can be determined in high‐throughput screenings by monitoring the hydrolysis of rather unspecific fatty acid‐based fluorogenic‐ or photoacoustic substrates.[^16^, ^17^, ^18^, ^19^] PET tracers for MAGL[^13^, ^14^] are useful tools in vivo due to their applicability in deep tissue. However, their inferior spatiotemporal resolution, short half‐life, and need for specialized facilities^[20]^ limit their applications in cellular settings. On the contrary, fluorescence‐based approaches allow for the non‐invasive, real‐time, high‐resolution, qualitative, and/or quantitative imaging of biological systems in diverse settings, such as cells, tissues, and animal models.^[21]^ Herein, we describe highly specific labeling and imaging probes for the direct and selective visualization of MAGL in its native state in vitro and in tissue samples with high spatiotemporal resolution.

## Results and Discussion

### Design of the MAGL Probe Platform

We first set out to construct a structural unit that would selectively and effectively recognize MAGL with high affinity while providing a versatile platform to host a wide variety of dyes with different physical and spectroscopic properties without significant unfavorable alteration of its characteristics. The probe blueprint was based on a reverse design approach considering pre‐existent structure–activity relationship (SAR) of synthetic drug‐like MAGL ligands with a defined pharmacological profile as starting points. We considered known MAGL ligands (Figure 1)[^13^, ^15^, ^22^, ^23^, ^24^, ^25^] for the construction of high‐quality, tailored, labeled probes.[^26^, ^27^] This leveraged the selection of suitable headgroups and spacers to address key pharmacophores and facilitated the identification of a suitable linker attachment point for the fluorophore reporter. In particular, the structure‐guided identification of a suitable linker region between the fluorophore reporter and the ligand subunit was crucial to allow the placement of a fluorophore outside the binding pocket (Supporting Information Figure S1). It is only in this way that the affinity and selectivity of the probe remain unaffected by changes in the fluorophore subunit, providing a robust platform for the generation of tailor‐made probes where a wide variety of fluorophores could be attached. Nevertheless, a moderate loss of affinity has to be expected for conjugated structures, compared to the parent ligands, due to suboptimal positioning of the linker and entropic effects.^[28]^ Analysis of available MAGL co‐crystal structures further revealed the key requirements for favorable interaction patterns for reversible and irreversible inhibition.[^6^, ^12^, ^29^] The general construction principle of these inhibitors generally follows a set of common pharmacophoric features. Namely, a carbonyl group that interacts with the key serine nucleophile (Ser122) of this serine hydrolase, an aromatic moiety occupies the hydrophobic pocket that accommodates the fatty acid of the endogenous 2‐AG ligand (referred to as left‐hand‐side, LHS) and a headgroup or electrophilic warhead that occupies the amphiphilic glycerol binding pocket (referred to as right‐hand‐side, RHS), as depicted in Figure 1. The central carbonyl (ketone, amide, carbamate, or urea) provides the key interactions with the oxyanion hole (backbone NH donors of Ala51 and Met123) in proximity to the Ser122/Asp239/His269 catalytic triad.^[30]^ MAGL inhibitors can be divided into two classes according to their mode of action into irreversible and reversible inhibitors. As for the irreversible inhibitors, the central carbonyl consists of an activated carbamate with good RHS leaving groups such as hexafluoro‐isopropanol (HFIP)^[31]^ or trifluoromethyl glycol (TFMG),^[32]^ which acylate the catalytic Ser122, while reversible inhibitors bear stable urea or amide structures.[^23^, ^33^, ^34^] Diverse cyclic amines predominantly bridge the space between the aromatic core and the central carbonyl moiety.

### Structure‐Activity Relationship Study

**Scheme 1:**
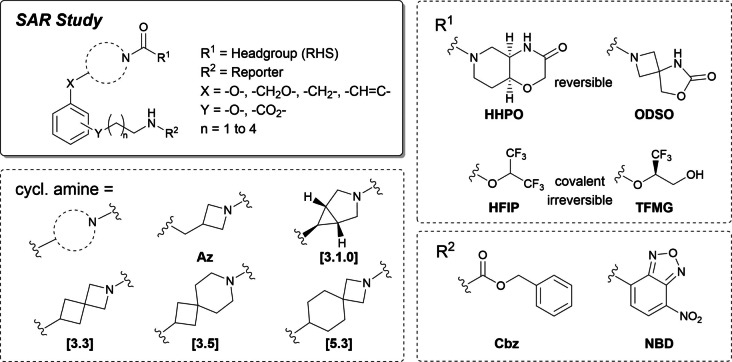
Privileged structural moieties used in the initial reverse design‐based structure–activity relationship (SAR) study.

We conducted a structure–activity relationship study to identify appropriate high‐affinity probes bearing LHS structures and headgroups. Following this we installed different linker lengths and exit vector orientations to probe their ability to still inhibit MAGL. The SAR study is summarized in Scheme 1 and Table 1, with eight key prototypic structures forming the cornerstones of the labeling platform. For detailed SAR data, see Supporting Information Tables S1–S5.

**Table 1:** The *s*elected key structures of the structure–activity relationship (SAR) study that guided the design of the probe platform with their respective half maximal inhibitory concentrations (IC_50_) values on human and mouse MAGL (hMAGL & mMAGL) and in the cell‐based nanoBRET assay using transfected HEK293 cells. *n*=1 to 3.

Cmpd.	R^1^	cycl. amine	X	Y	n	R^2^	hMAGL IC_50_ [nM]	mMAGL IC_50_ [nM]	HEK293 nanoBRET IC_50_ [nM]
1	HHPO	Az	CH_2_O	–	–	–	379	526	>10,000
2	HHPO	Az	CH_2_O	*m*‐O	1	Cbz	30.8	97.4	406
3	HHPO	Az	CH_2_O	*o*‐O	1	Cbz	2.14	5.41	141
4	HHPO	Az	CH_2_O	*o*‐O	1	NBD	3.92	4.53	2,560
5	ODSO	Az	CH_2_O	*o*‐O	1	Cbz	0.29	0.59	61
6	ODSO	Az	CH_2_O	*o*‐O	1	NBD	0.72	1.03	318
7	HFIP	Az	CH_2_O	*o*‐O	1	Cbz	0.65	0.85	222
8	ODSO	[3.3]	CH_2_	*o*‐O	1	NBD	0.09	0.11	307

We started our investigation by selecting privileged azetidine and azetidine‐like structures as scaffolds for the LHS cyclic amine moiety due to their high affinity in MAGL reversible and irreversible inhibitors.[^23^, ^24^, ^35^, ^36^] A benzyl ether was connected to the cyclic amine to facilitate synthetic access to prototypic ligands for initial probe assessment and profiling. Compound **1** combines 3‐((benzyloxy)methyl) azetidine as a lipophilic LHS with the privileged HHPO^[22]^ (see Scheme 1) headgroup via a central urea structure. The initial structure **1** showed moderate MAGL inhibitory potency with an IC_50_ of 379 nM on human MAGL (hMAGL). A docking study (Supporting Information Figure S1) identified the *ortho* and *meta* positions of the aromatic residue as suitable candidates for linker attachment. Ester, amide, and ether connections were tested at this exit vector region, with *ortho*‐ and *meta*‐aryl ether structures having the highest potency against MAGL. After testing different cyclic amine analogs (Scheme 1 and Supporting Information Tables S1–S2), we found that the benzyl ether motif was suitable for generating a set of MAGL ligands with IC_50_ values ranging from 1 to 100 nM against human MAGL (Supporting Information Tables S1–2) with **2** and **3** demonstrating the superiority of the *ortho* exit vector. Notably, adding the Cbz‐protected aminoethoxy linker structure increased potency towards MAGL by up to two orders of magnitude, suggesting favorable interaction of the linker moiety and MAGL at the exit region of the lipophilic pocket.

Compared to prominent MAGL reference compounds (JZL184,^[37]^ MJN110,^[38]^ LEI‐463^[15]^), the series was devoid of a second phenyl ring and a (pro)chiral center at the LHS, thereby reducing its complexity, size, and lipophilicity. Derivative **3** was selected to test the design for its potential for conjugated fluorescent probe generation. Cbz deprotection and conjugation of the amine with nitrobenzoxadiazole (NBD) yielded a first fluorescent probe **4**, which showed similar activity against hMAGL (IC_50_=3.92 nM) as precursor **3**, indicating no detrimental interaction between the fluorophore and the protein.

Demonstrating cellular delivery and target engagement is crucial for an intracellular target such as MAGL. We, therefore, confirmed cellular MAGL target engagement in a nanoBRET assay using live HEK293 cells.^[39]^ Here, we observed that NBD probe **4** was much less active than the Cbz‐protected analog **3**, most probably due to a drastic increase in polarity (**3** vs **4**: HBA 7→10, tPSA 119 Å^2^→177 Å^2^, clogP 1.81→0.95) significantly limiting the passive permeation capability, thus lowering intracellular probe concentration, as observed by the apparent cellular IC_50_. To overcome the low cellular potency of **4**, we exploited the modular construction principle to modulate unfavorable physicochemical properties and investigated less polar, privileged head groups[^23^, ^33^, ^40^] (Supporting Information Table S4). We found that the replacement of the HHPO headgroup with a spiro azetidine carbamate (7‐oxa‐2,5‐diazaspiro[3.4]octan‐6‐one (ODSO)[^23^, ^41^] showed improved affinity towards MAGL for the Cbz‐protected (**5**) and NBD‐labeled (**6**) analogs by approximately one order of magnitude. Moreover, the substitution resolved affinity differences between species isoforms. Therefore, probes bearing the ODSO headgroup were selected for further probe optimization.

**Figure 2:**
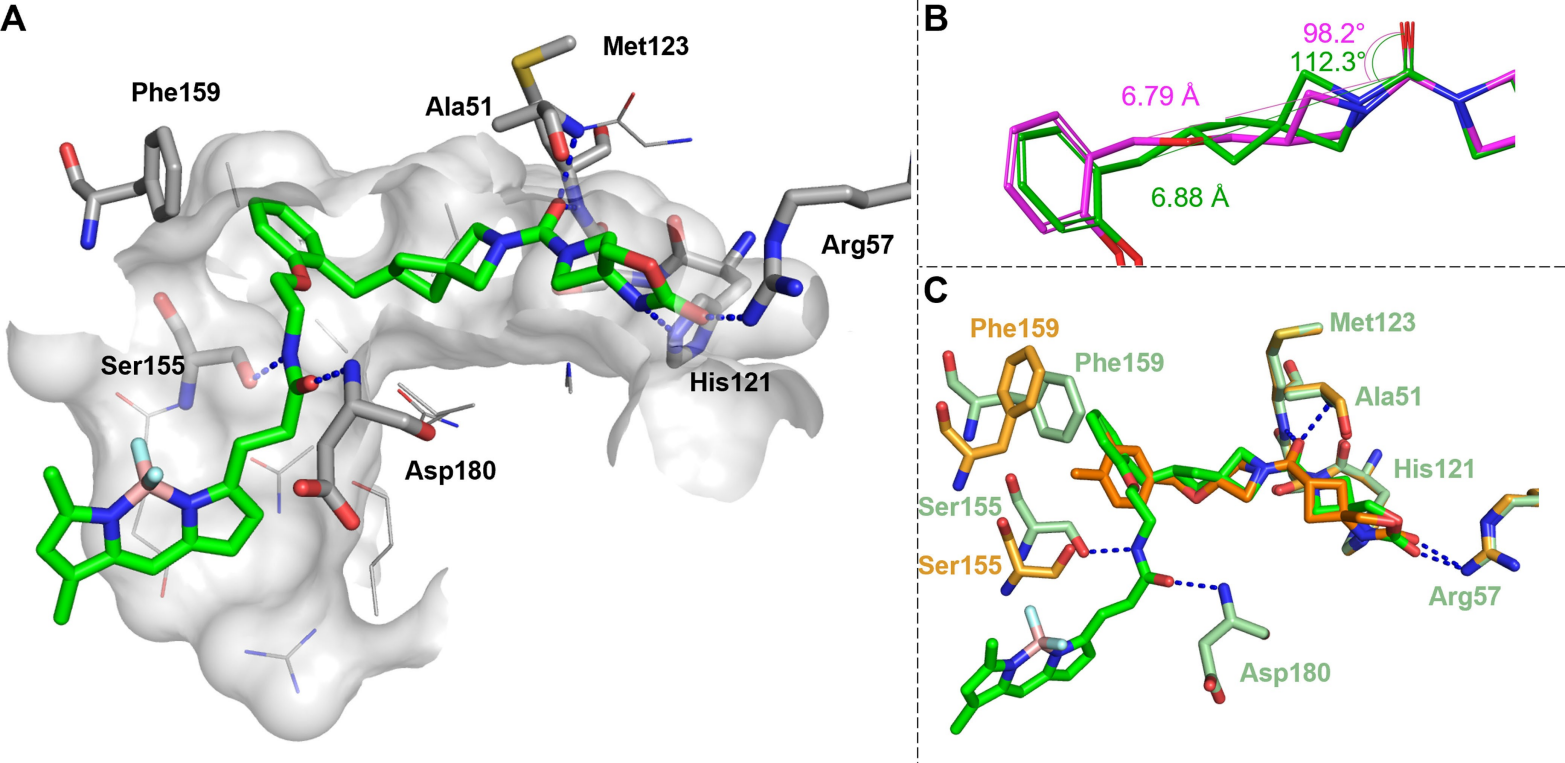
A) The co‐crystal structure of compound **9** (green) with hMAGL (PDB: 9G4M). Hydrogen bonds (blue) are indicated as dashed lines. Amino acid residues with important interactions (Phe159, Ser155, Asp180, Met123, Ala51, His121, Arg57) are highlighted. B) The spirocyclic [3.3] structure of **9** (green) is a rigidified bioisostere of the azetidine ether structure of **5** (magenta). The structure of the minimized conformer of **5** is superimposed with the ligand structure of the X‐ray co‐crystal structure (PDB: 9G4M), truncated for clarity. The distance between the carbonyl C and the benzyl C and the respective angles to C=O are indicated. C) Superimposition of the binding sites of compounds **4 f** (orange, PDB: 7L50) and **9** (green, PDB: 9G4M).

In‐silico analysis by flexible alignment studies suggested that the considerable molecular flexibility of the azetidine LHS in **2**–**6** could be limited by the introduction of a spirocyclic moiety locking the bioactive conformation while omitting the benzylic oxygen.[^42^, ^43^, ^44^] Replacement of the simple benzyloxymethyl azetidine motif by a spirocyclic [3.3]‐amine structure yielded key probe **8** (Figure 2 B), which showed significantly improved MAGL affinity up to the sub‐nanomolar range.

Remarkably, by exchanging the diazaspiro octanone headgroup with an electrophilic HFIP‐carbamate[^36^, ^45^, ^46^] or TFMG‐carbamate,^[24]^ as irreversible MAGL warheads resulted in irreversible fluorescent probe **7** exhibiting a high affinity for MAGL without any further modification required.

**Scheme 2:**
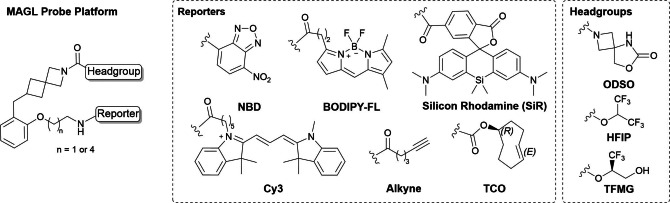
Key structures of MAGL probes with the optimal headgroups and reporter unit combinations were used throughout this investigation.

Overall, this optimization led to the identification of a MAGL probe platform (Scheme 2) with reversible or irreversible, high‐affinity MAGL binders that can be utilized to tailor versatile MAGL labeling probes **8**–**14** (Table 2).

**Table 2:** In vitro characterization of the optimized fluorescent MAGL probes. Half maximal inhibition concentration (IC_50_) is represented as the mean from three independent measurements±standard deviation (SD). Fluorescence excitation and emission maxima determined in PBS at pH 7.4.

Cmpd.	Headgroup	MoA	n	Reporter	hMAGL IC_50_±SD [nM]	mMAGL IC_50_±SD [nM]	HEK 293 nanoBRET IC_50_±SD [nM]	λ_max_(Ex/Em) [nm]
**8**	ODSO	reversible	1	NBD	0.09±0.03	0.12±0.04	308±114	479/554
**9**	ODSO	reversible	1	BODIPY‐FL	0.12±0.05	0.14±0.07	162±104	508/514
**10**	ODSO	reversible	4	SiR	0.15±0.11	0.16±0.09	285±240	654/673
**11**	HFIP	irreversible	1	BODIPY‐FL	0.91±0.39	0.91±0.45	>1000^[a]^	508/514
**12**	HFIP	irreversible	4	SiR	1.15±0.29	1.35±0.30	>1000^[a]^	654/673
**13**	HFIP	irreversible	1	Alkyne	0.14±0.04	0.13±0.05	99±23	n.a.
**14**	TFMG	irreversible	4	TCO	0.31±0.13	0.42±0.17	1253±507	n.a.

[a] The fluorophores interfered with the nanoBRET assay performance at elevated concentrations above 1 μM.

### Elucidation of the Binding Mode by X‐Ray Crystallography

We obtained a co‐crystal structure of BODIPY labeled probe **9** in complex with hMAGL (PDB: 9G4M, Figure 2 A). This provided insights into the binding mode of the ligand platform and the structural determinants for the observed high affinity and the placement of the reporter moiety outside the binding pocket (Figure 2 A). Analysis of the co‐crystal structure showed that the MAGL binding moiety, consisting of the ODSO headgroup, the central carbonyl, and the lipophilic LHS, deeply protrudes the catalytic pocket of hMAGL. The ODSO headgroup forms hydrogen bonds with the Arg57 and His121, similar to the structural analog compound **4 f** ^[23]^ (Figure 2 C). A hydrogen bonding network in the oxy‐anion hole consisting of Ala51 and Met123 firmly anchors the central carbonyl. The LHS phenyl moiety directly interacts by a T‐shaped pi‐pi stacking with Phe159. This residue is part of the α4 helix lid domain of MAGL, which is here in its closed conformation, thereby limiting the size of the lipophilic pocket. The optimized *ortho‐*alkoxy linker is placed at an exit vector at the narrow entry side, where the aminoethoxy bridge is in the favorable gauche conformation. Additionally, the linker amide forms an H bond with Asp180 at the protein‘s surface. However, this interaction does not appear to be essential for high‐affinity binding, as comparison of matched molecular pairs with pentyl instead of the ethylene linker have at least an on‐par affinity towards MAGL.

### Synthesis

**Scheme 3:**
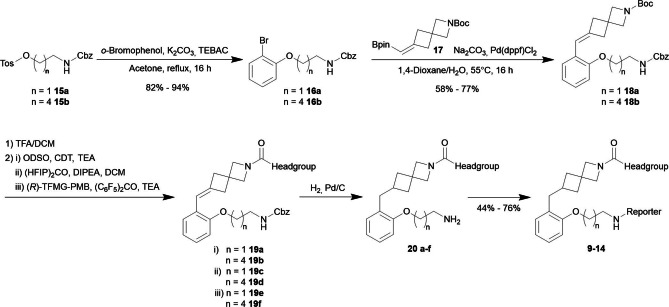
General synthesis scheme to access MAGL probes **9** to **14**.

The synthesis of the probe platform started from *o*‐bromo phenoxy building blocks **16 a** and **16 b** by coupling the spirocyclic building block **17** in a Suzuki reaction to the LHS‐building block precursors **18 a** and **18 b** (Scheme 3). The vinyl boronic acid pinacol (Bpin) ester **17** was generated from the respective ketone via a Boron‐Wittig olefination[^47^, ^48^] using bis[(pinacolato)boryl]‐methane. *N*‐Boc deprotection of the LHS‐building block precursors was followed by connection to the head group via an installation of the central urea structure of the reversible probes (**19 a**–**b**) and carbamates (**19 c**–**f**) of the irreversible probes, which were achieved under standard conditions.

Hydrogenation of the double bond and simultaneous liberation of the free amine by hydrogenolytic Cbz‐cleavage yielded intermediates **20 a**–**f**. For conjugation of the reporter units, a variety of standard coupling reactions were used, e.g., with fluorophore carboxylic acids via HATU coupling or NHS esters, NBD derivatives via S_N_Ar with NBD‐fluoride to afford the target molecular probes **9**–**14** (Table 2) in good yields.

### Probe Profiling and Target Engagement with MAGL

Next, we investigated whether fluorescent probes **9**–**12** carrying BODIPY‐FL and SiR fluorophores would retain their affinity and overall specificity for visualizing and detecting MAGL in cellular settings. Specificity and selectivity is of paramount importance when developing chemical probes.^[27]^ All probes showed negligible species differences in IC_50_ values between human and mouse MAGL, an important requirement for the translatability of different pharmacological models (Table 2). The irreversible, covalent mode of action was confirmed via mass spectrometry of purified human MAGL enzyme, where incubation with the irreversible probe **12** yields the expected labeled protein mass (Supporting Information Figure S3).

**Figure 3:**
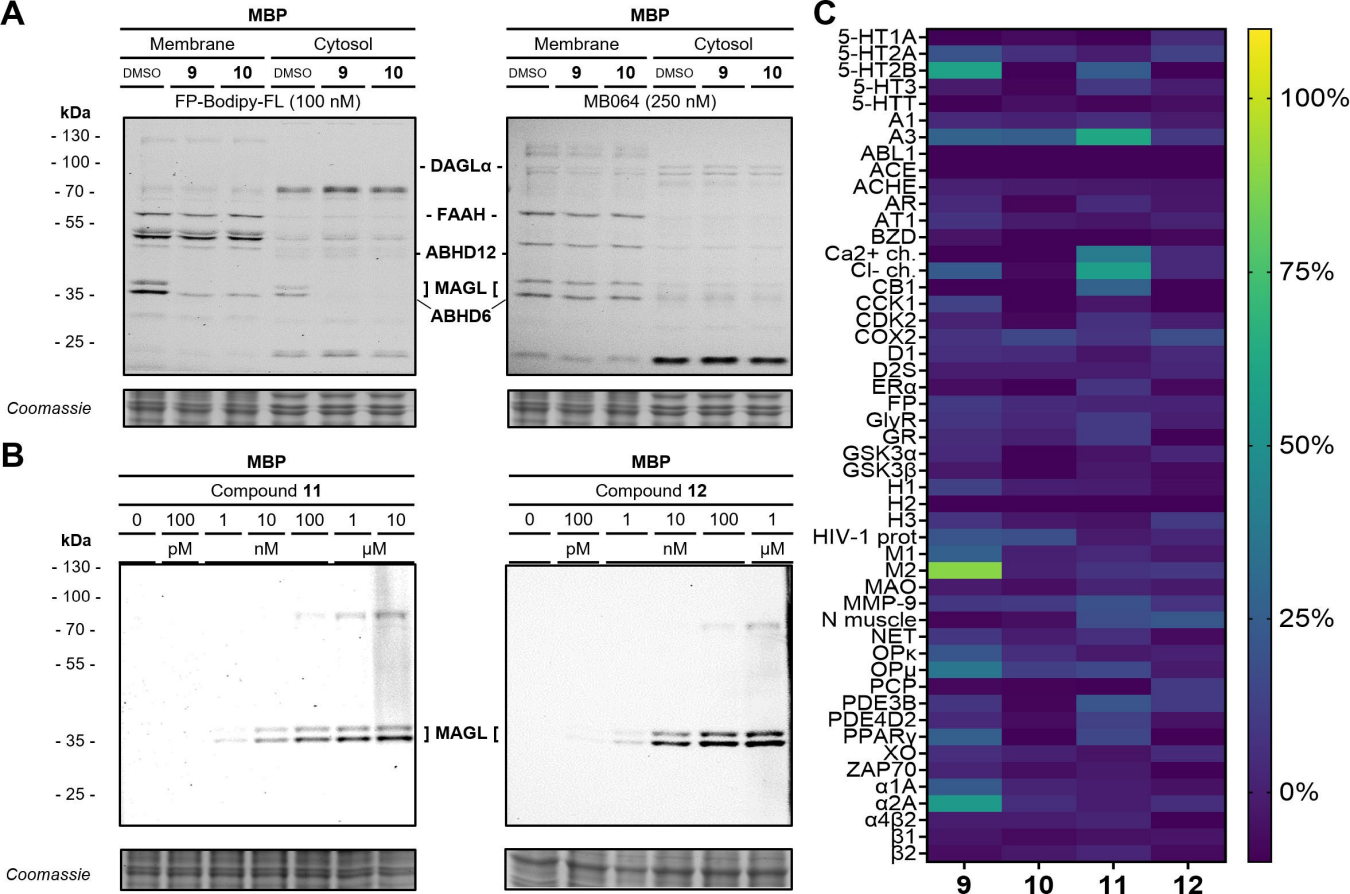
Assessment of the selectivity and specificity of the fluorescent MAGL probes **9** to **12** A) Multiplex ABPP assay labels a variety of serine hydrolases in the membrane and cytosolic fraction of the mouse brain proteome (MBP). The two MAGL bands at approx. 35 kDa are selectively blocked by 1 μM of reversible probes **9** and **10**. Both panels depict the same representative SDS‐PAGE at the respective fluorescent channel settings. B) Dose‐dependent labeling of the two MAGL splicing variants in MBP with the two irreversible fluorescent probes **11** and **12**. C) Heatmap representation of off‐target panel screen of **9** to **12** at 10 μM (ca. 10,000‐fold MAGL IC_50_). A brighter color indicates higher inhibition.

Activity‐based protein profiling (ABPP)^[49]^ was used to determine the selectivity of the reversible probes (**9**, **10**) versus a broad range of other serine hydrolases, including the relevant ECS off‐targets DAGL, FAAH, ABHD6, and ABHD12.[^45^, ^49^, ^50^, ^51^] The ABPP assay was studied in the mouse brain proteome (MBP) at 1 μM concentration as mouse brain homogenate is the most relevant model system for studying ECS enzymes.[^45^, ^49^, ^50^, ^51^] Complete blocking of the fluorescence signal was exclusively observed for MAGL, indicating a very high selectivity of probes **9** and **10** (Figure 3 A). The irreversible probes **11** and **12** could be used to visualize MAGL by in‐gel SDS‐PAGE fluorescence of MBP samples and, thereby, directly showing the specificity of **11** and **12** towards the two MAGL splicing variants present in MBP over a broad range of concentrations. Starting at 1 nM probe concentration, MAGL was detected and only at elevated concentrations greater than 1 μM, minor off‐target protein labeling at approx. 80 kDa started to appear (Figure 3 B). In addition to testing in mouse brain lysates, we also determined the binding of MAGL probes **9**–**12** towards a panel of 50 off‐targets via a competitive radioligand binding screen to detect interference with potential safety‐related off‐targets (Figure 3 C).

Even at a very high concentration of 10 μM, corresponding 10,000‐fold of the IC_50_ MAGL, no relevant off‐target binding was observed for probes **9**–**12**, which is in line with the results obtained by profiling the mouse brain lysates.

### ABBP Assay: Two‐Step Labeling and Target Occupancy Assessment

For a variety of cellular investigations, two‐step labeling using clickable handles in activity‐based probes can significantly extend the scope of applicable reporters and experimental settings.^[52]^ Potential application of click‐chemistry probes could be in the identification of transient protein‐protein interactions or in studying the turnover of MAGL in cellular environments. This strategy also facilitates the use of diverse chemical reporters, including biotin for streptavidin pull‐down assays, fluorophores for super‐resolution microscopy, radiolabels for autoradiography, or spin labels for electron paramagnetic resonance spectroscopy. We therefore installed the respective alkyne and trans‐cyclooctene (TCO) handles for two‐stage labeling via click chemistry in **13** and **14**, respectively, bearing irreversible HFIP or TFMG warheads. To balance out the lipophilicity characteristics of the probe and avoid solubility issues due to the presence of the lipophilic TCO moiety, we used TCO in combination with the more polar TFMG warhead.

**Figure 4:**
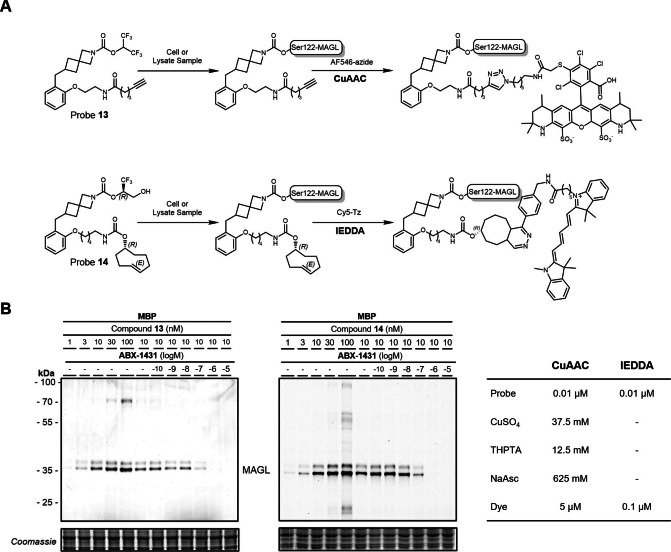
Probes **13** and **14** selectively and specifically bind to MAGL covalently and can be functionalized in a second step via click chemistry. A) Reaction scheme of probes **13** and **14** with MAGL and with **AF546‐azide** and **Cy5‐Tz**, respectively. The table gives an overview of CuAAC and IEDDA reaction conditions, 30 min, r.t. B) Dose‐dependent labeling of the two MAGL splicing variants in MBP with **13** and **14** and respective click labeling. The MAGL fluorescence signal is **ABX‐1431** dose‐dependent.

While usage of copper‐catalyzed azide‐alkyne cycloaddition (CuAAC) may encounter limitations^[53]^ in live cells and tissue due to the cytotoxic interference by the reagents used, i.e. copper(I) and ligands such as THPTA, bio‐orthogonal strain‐promoted click reactions with TCO exhibit high reaction rates even without a catalyst.^[54]^ Irreversible probes **13** and **14** were incubated with mouse brain homogenates for specific and selective labeling of MAGL (Figure 4). First, the lysate was treated with probe **13** (1–100 nM) for 30 min at room temperature for covalent labeling of MAGL. The sample was then subjected to the CuAAC reaction with **AF546‐azide** (5 μM).

Similarly, we used probe **14** with the TCO handle for a biorthogonal inverse electron demand Diels–Alder conjugation reaction (IEDDA) with Cy5 dye connected to a reactive tetrazine (**Cy5‐Tz**, 0.1 μM) reaction under a similar setting but in the absence of any catalyst. Probes **13** and **14** gave comparable results for the specific labeling of MAGL in mouse brain homogenate (Figure 4 B). The observed very high specificity for the two‐step labeling of MAGL by irreversible probes **13** and **14** could be further confirmed by dose‐dependent signal depletion, upon pre‐incubation with the non‐labeled, irreversible, MAGL‐selective inhibitor **ABX‐1431** (Elcubragistat)^[25]^ (Figure 4 B).

**Figure 5:**
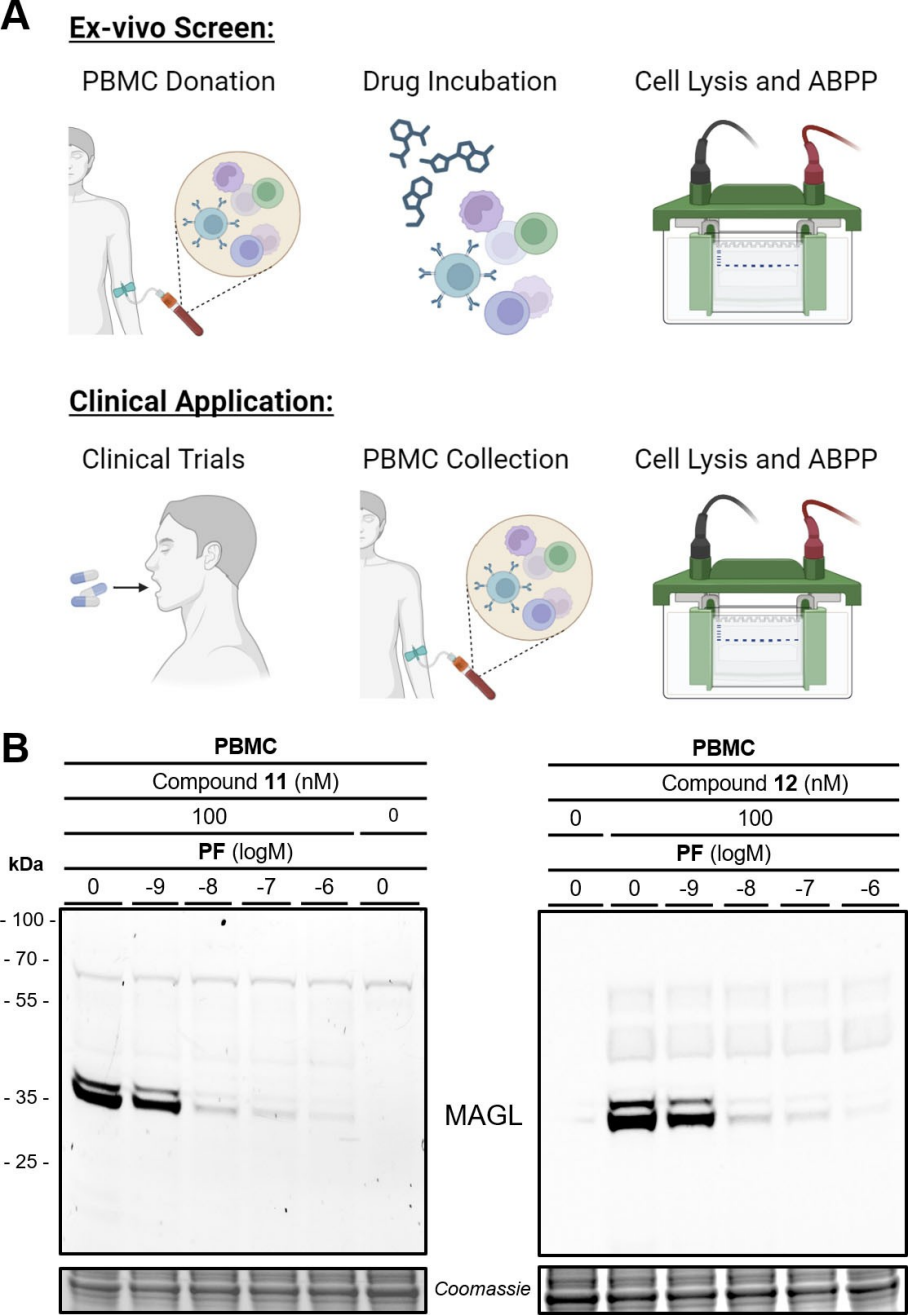
Fluorescent probes **11** and **12** are functional ABPP drug‐target‐occupancy probes in patient‐derived peripheral blood monocytes (PBMC). A) Schematic overview of either ex vivo target occupancy screens or clinical use to determine drug‐target engagement. B) SDS‐PAGE in gel fluorescence of MAGL‐specific ABPP probes **11** and **12** at 100 nM concentration is PF‐06795071 (**PF**) dose‐dependent. Both panels depict the same representative SDS‐PAGE at the respective fluorescent channel settings and Coomassie protein staining as a loading control.

In particular, the very high potency and specificity of the developed MAGL probes offer the opportunity to assess ex vivo target occupancy in clinical applications as a diagnostic biomarker. In this context, patient‐derived peripheral blood monocytes (PBMC) are an essential model system allowing direct readout of drug action in metabolic disorders and inflammatory processes.^[55]^ We established therefore an ex vivo ABPP assay using MAGL‐specific ABPP probes **11** and **12** to determine vacant MAGL in the PBMCs after sample lysis. The drug‐target‐occupancy of the MAGL‐selective inhibitor **PF** ^[24]^ in intact patient PBMCs was investigated over a wide concentration range of 1 nM to 1000 nM. The corresponding target occupancy of **PF** was reliably visualized and quantified with SDS‐PAGE in‐gel fluorescence (Figure 5). These results in native, primary PBMCs show that the labeled probes can be used to determine the target occupancy in patients under clinical settings to assess the drug action on MAGL activity (Figure 5).

### Visualization of MAGL in Live Cancer Cells and Neurons

**Figure 6:**
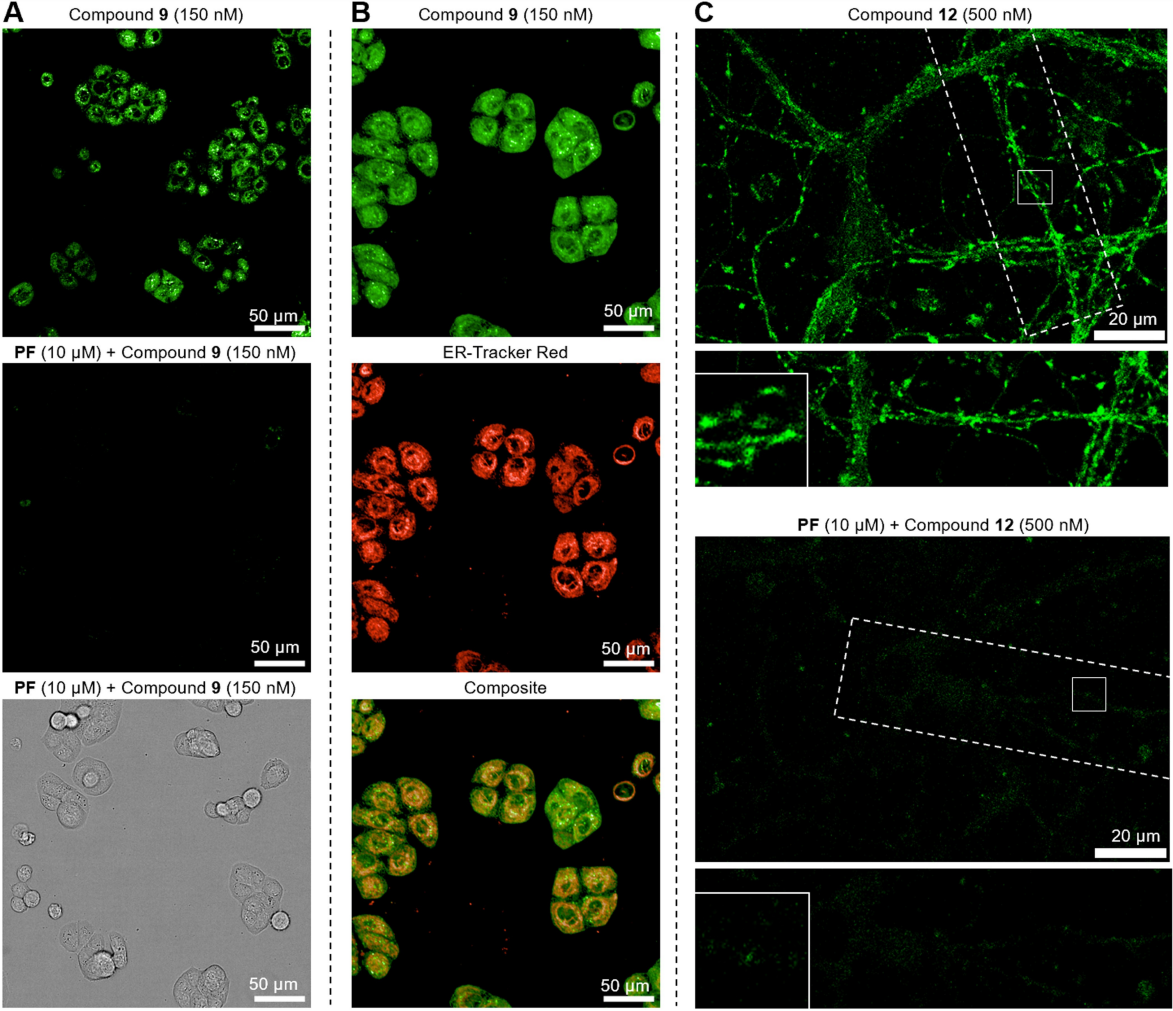
Fluorescence microscopy applications of fluorescent MAGL probes **9** and **12**. A) HT‐29 cells are stained with probe **9** (150 nM), with and without preincubation of 10 μM MAGL specific inhibitor PF‐06795071 (**PF**), showing specific MAGL labeling. B) Compound **9** MAGL labeling colocalizes with ER‐Tracker Red (Invitrogen). Green spherical structures can be observed that do not correlate with ER‐tracker. C) Confocal images of the live dissociated primary hippocampal neuron culture at day‐in vitro 14–21. Cells were treated with **12** (0.5 μM) at 37 °C for 15 min; negative controls were treated with **PF** (10 μM) for 2 h prior to MAGL labeling. Neuronal processes (dashed line) and potential synapses (insets) are highlighted.

With these highly selective probes in hand, we next investigated their ability to specifically visualize MAGL in live cell imaging applications. The enzymatic activity of MAGL plays a critical role in cancer lipid metabolism and is overexpressed in several particularly aggressive cancer cell lines.^[56]^ Therefore, the expression levels of MAGL are considered a prognostic biomarker for the degree of cancer tumor differentiation and progression.^[57]^ Here the colon cancer HT‐29 cell line is one of the prototypical systems regarding the impact of MAGL in proliferating and invasive cancer.[^45^, ^58^, ^59^] We studied the ability of probes **8**–**12** to detect MAGL by confocal imaging microscopy (Figure 6 A). Consistent staining of MAGL could be achieved with all optimized probes **8**–**12** carrying NBD, BODIPY, and SiR, respectively, providing a bright and stable fluorescent signal in live cancer cells. In addition, no signs of toxicity were observed over prolonged incubation times for all probes **8**–**12** in the applied concentration range of 0.15 to 10 μM.

As expected, the more lipophilic probe construct bearing Cy3 (**S21**, see Supporting Information Table S5) was more prone to unspecific accumulation in membranes, and excess probe was, therefore, more difficult to remove by washing steps due to a more adherent behavior. A decreased specificity and adherent behavior of overly lipophilic drugs and chemical probes is a well‐known obstacle in their design process.[^60^, ^61^] Nevertheless, probes such as **S21** may be of interest for application for both cell‐free assays and tissue lysates. BODIPY‐FL labeled probe **9**, which has a well‐equilibrated lipophilicity profile (clogP=2.21, vs. 5.76 for **S21**), showed a very specific and selective MAGL staining in HT‐29 cells at 0.15 μM, even without any additional washing steps (Figure 6 A). The selectivity was confirmed by pre‐incubation with the MAGL‐selective inhibitor **PF** (10 μM), which completely suppressed the fluorescent signal. To further demonstrate the general applicability of probe **9**, we investigated additionally A549 cells, a human lung carcinoma cell line, that has been previously reported as a model system of MAGLs’ influence on invasiveness and metastasis.^[62]^ Also here we could observe a bright staining under equivalent conditions (Supporting Information Figure S4).

We then investigated the subcellular localization of MAGL in HT‐29 using probe **9**. Strong signal co‐localization with ER tracker red (Figure 6 B; Pearson correlation coefficient r=0.99) supported previous investigations about its subcellular localization.[^45^, ^63^] Co‐staining with MitoTracker or LysoTracker showed no strong correlation, indicating that MAGL is not extensively localized in mitochondria or lysosomes in this cell line (Supporting Information Figure S5). Besides the ER staining, dense MAGL localization was observed in small, spherical structures reminiscent of lipid droplets. This observation is in agreement with the reported role of lipid droplets and MAGL in cancer metabolism.[^64^, ^65^]

MAGL plays a vital role in neuronal inflammation[^24^, ^66^, ^67^] and the integrity of the blood–brain barrier.[^68^, ^69^] Therefore, we investigated the imaging capability of the probes in live dissociated primary hippocampal neuron cultures (Figure 6 C) as a prime model system in CNS research, particularly in the context of its role in shaping neurotransmitter release and synaptic plasticity properties.^[70]^ Confocal microscopic imaging of the live cultures, at day 14–21 in vitro, was performed on cells treated with probe **9** (1 μM) and the particularly bright silicon‐rhodamine (SiR) probes **10** and **12** (0.5 μM) at 37 °C for 15 min, all of them producing a strong fluorescent signal. Irreversible probe **12** proved to be optimal in this experimental setting, as an intense fluorescent signal was observed along regions of neuronal processes (Figure 6 C) with minimal noise from autofluorescence or unspecific staining. The specificity of the MAGL labeling was confirmed via pre‐incubation with MAGL‐specific inhibitor **PF** (10 μM), which led to a strong decrease in mean fluorescent intensity (p <0.01). We observed an accumulation of the fluorescent signal in neuronal varicosities with a distribution that is reminiscent of that of synapses, indicating the localization of MAGL. Overall, these findings show that our probes can assist in fluorescent labeling and detection of MAGL in live, native cells with a wide variety of state‐of‐the‐art fluorophores that will support elucidating MAGL‐dependent physiological and pathophysiological processes.

## Conclusion

We reported herein the development and optimization of a fluorescent probe platform that selectively targets monoacylglycerol lipase (MAGL), a key therapeutic target within the endocannabinoid system. Based on a rational reverse design approach from known MAGL inhibitor structures, a thorough structure–activity relationship study and analysis of structural information from X‐ray crystallography guided the development of highly selective and versatile probes. The probe platform allows the selection of a reversible labeling mode using the ODSO headgroup or irreversible, covalent activity‐based trapping of MAGL with either HFIP or TFMG serine warheads. The utilization of a very broad range of fluorophores ranging from, i.e., NBD, Cy3, BODIPY‐FL, TAMRA, SiR, or a click chemistry handle such as alkyne or TCO functionalities for two‐step labeling was well tolerated. The conjugated probes show appropriate solubility and cell permeability, and the selectivity remained invariant to changes in the reporter subunit, demonstrating great robustness for the construction of tailor‐made probes. In addition, we were able to obtain a high‐resolution co‐crystal structure of the conjugated ligand in complex with hMAGL, which provided insight into key structural features determining the binding mode and greatly facilitated the modular construction of the probe platform. Their applicability for MAGL detection and visualization was demonstrated in various settings, ranging from MAGL ABPP probes in cellular lysates of clinically relevant patient PBMC cell samples over bio‐orthogonal covalent modification of native MAGL protein click chemistry to confocal fluorescence microscopy imaging of MAGL in live neuronal cells and cancer cells with very high subcellular spatiotemporal resolution. Among the variety of synthesized reversible conjugates, MAGL‐selective BODIPY probe **9** stood out, showing well‐balanced physicochemical properties, photochemical brightness, and useability in cellular staining protocols. For irreversible fluorescent labeling, SiR probe **12** consistently provided superior results in specific MAGL detection in human PBMC samples as well as in live neuronal cells. Besides fluorescent detection, probe **14** enabled bio‐orthogonal covalent modification of native MAGL protein via TCO‐Tz click chemistry, thereby enabling basic biochemical research on native MAGL.

Overall, we identified the first highly selective and specific MAGL labeling platform. Given the rapidly growing interest and high relevance of MAGL as a pharmaceutical target, we believe that our probes can serve as chemical tools for unraveling the manifold (patho)physiological roles and functions of MAGL in the endocannabinoid system and beyond.

## Abbreviations

AF546: Alexa Fluor 546
2‐AG: 2‐arachidonoylglycerol
ABPP: activity‐based protein profiling
AF546: Alexa Fluor 546
CB1: cannabinoid receptor type 1
CB2: cannabinoid receptor type 2
CNS: central nervous system
Cmpd: compound
CuAAC: copper(I)‐catalyzed azide‐alkyne cycloaddition
Cy: cyanine dye—tetramethylindo(di)‐carbocyanines
DAGL: diacylglycerol lipase
ECS: endocannabinoid system
FAAH: fatty acid amide hydrolase
HFIP: hexafluoroisopropanol
HHPO: (4a*R*,8a*S*)‐hexahydro‐2*H*‐pyrido[4,3‐b][1,4]oxazin‐3(4H)‐one
IC_50_
: half maximal inhibitory concentration
IEDDA: inverse electron demand Diels–Alder
LD: lipid droplets
LHS: left‐hand side
MAGL: monoacylglycerol lipase
MBP: mouse brain proteome
MoA: mechanism of action
NaAsc: sodium ascorbate
NBD: nitrobenzoxadiazole
ODSO: 7‐oxa‐2,5‐diazaspiro[3.4]octan‐6‐one
PBMC: peripheral blood mononuclear cells
PET: positron emission tomography
RHS: right‐hand side
SAR: structure–activity relationship
SDS‐PAGE: sodium dodecyl sulfate polyacrylamide gel electrophoresis
SiR: silicon rhodamine
TCO: trans‐cyclooctene
TFMG: trifluoromethyl glycol
THPTA: tris(3‐hydroxypropyltriazolylmethyl)amine

## Supporting Information

Primary data for methodology, fluorescence imaging, probe

synthesis, and characterization are provided in the Supporting Information. The authors have cited additional references within the Supporting Information.[^71^, ^72^, ^73^, ^74^, ^75^, ^76^, ^77^, ^78^, ^79^]

## Conflict of Interests

The authors declare no conflict of interest.

1

## Supporting information

As a service to our authors and readers, this journal provides supporting information supplied by the authors. Such materials are peer reviewed and may be re‐organized for online delivery, but are not copy‐edited or typeset. Technical support issues arising from supporting information (other than missing files) should be addressed to the authors.

Supporting Information

## Data Availability

The data that support the findings of this study are available in the supplementary material of this article.
