# Supplementary material for: A Highly Selective and Versatile Probe Platform for Visualization of Monoacylglycerol Lipase
Source: Angew Chem Int Ed Engl. 2025 Feb 7;64(10):e202413405. doi: 10.1002/anie.202413405 (PMC11878344; doi:10.1002/anie.202413405)
Supplement: Supplementary file 1 — Supporting Information [file ANIE-64-e202413405-s001.pdf]

Angewandte Chemie
Eine Zeitschrift der Gesellschaft Deutscher Chemiker

Supporting Information

A Highly Selective and Versatile Probe Platform for Visualization of Monoacylglycerol Lipase

A. Hentsch, M. Guberman, S. Radetzki, S. Kaushik, M. Huizenga, J. Paul, M. Schippers, J. Benz, B. Kuhn, D. Heer, A. Topp, L. Esteves Gloria, A. Walter, R. Hochstrasser, M. B. Wittwer, J. P. von Kries, L. Collin, J. Blaising, M. van der Stelt, N. Lipstein, U. Grether, M. Nazaré**

Supporting Information

A Highly Selective and Versatile Probe Platform for Visualization of Monoacylglycerol Lipase

Axel Hentsch# [a], Mónica Guberman# [a], Silke Radetzki [a], Sofia Kaushik [a], Mirjam Huizenga [b], Jerome Paul [a], Maria Schippers [c], Jörg Benz [c], Bernd Kuhn [c], Dominik Heer [c], Andreas Topp [c], Ludivine Esteves Gloria [c], Alexander Walter [c], Remo Hochstrasser[c], Matthias B. Wittwer [c], Jens Peter von Kries [a], Ludovic Collin [c], Julie Blaising [c], Mario van der Stelt [b], Noa Lipstein [a], Uwe Grether *[c], and Marc Nazaré *[a]

Contents

1. Probe Design and Optimization 2

2. Synthesis and Characterization of the Probe Molecules 9

2.1. General Methods 9

2.2. Main Text Compounds 10

2.3. Further Supporting Information Compounds 27

2.4. Building Blocks 34

3. Supporting Experiments 43

3.1. Mass-Spectroscopic Proof of Covalency 43

3.2. Confocal Fluorescence Imaging of Additional Cells 44

3.3. Photophysical Characterization 46

4. Experimental Details of Biochemical Assays and Crystal Structure 48

5. NMR Spectra of Key Probes 8–14 55

1. Probe Design and Optimization

As a starting point for our probe design, we chose the (4a*R*,8a*S*)-hexahydro-2*H*-pyrido[4,3-b][1,4]oxazin-3(4H)-one (HHPO)[22] due to their high affinity, selectivity, and overall favorable ADME properties. For the left-hand-side (LHS), azetidine-like structures were chosen, which are privileged scaffolds that have shown improved lipophilicity and good affinity for MAGL irreversible and reversible inhibitors.

We conceived that connecting a benzyl ether to the cyclic amine should allow for easy synthetic access to model molecules for an initial assessment and probe profiling. A docking study allowed the identification of ortho and meta positions of the aromatic residue as suitable candidates for the attachment of an exit vector (Figure S1). Initial screening of probes containing different cyclic amines (azetidine, spirocyclic, and fused ring) indicated that the benzyl ether motif for the LHS was suitable for generating MAGL ligands with IC_{50} values in the 1-100 nM range for human MAGL (Table S1, entries 1-6). Analogs of compounds **S1**-**S3** carrying the linker in *meta* position of the aromatic ring resulted in slightly higher IC_{50} values than their *ortho*-substituted counterparts and lower-yielding synthetic routes (Table S2). Methylene azetidine derivative **S2 (3)** with IC_{50} values of 2 and 5 nM for humane and murine variants, respectively, was identified as the best candidate from this series and used as a reference compound for further optimization. An increase in the length of the linker's alkane chain (compounds **S7**-**S8**) did not result in an improved affinity for model compounds bearing a carboxybenzoyl (Cbz) protecting group at the end of the linker chain. With these results in hand, derivative S2, bearing an ethanolamine-type linker, was selected as a model ligand to proceed with fluorophore attachment for proof-of-principle evaluation.

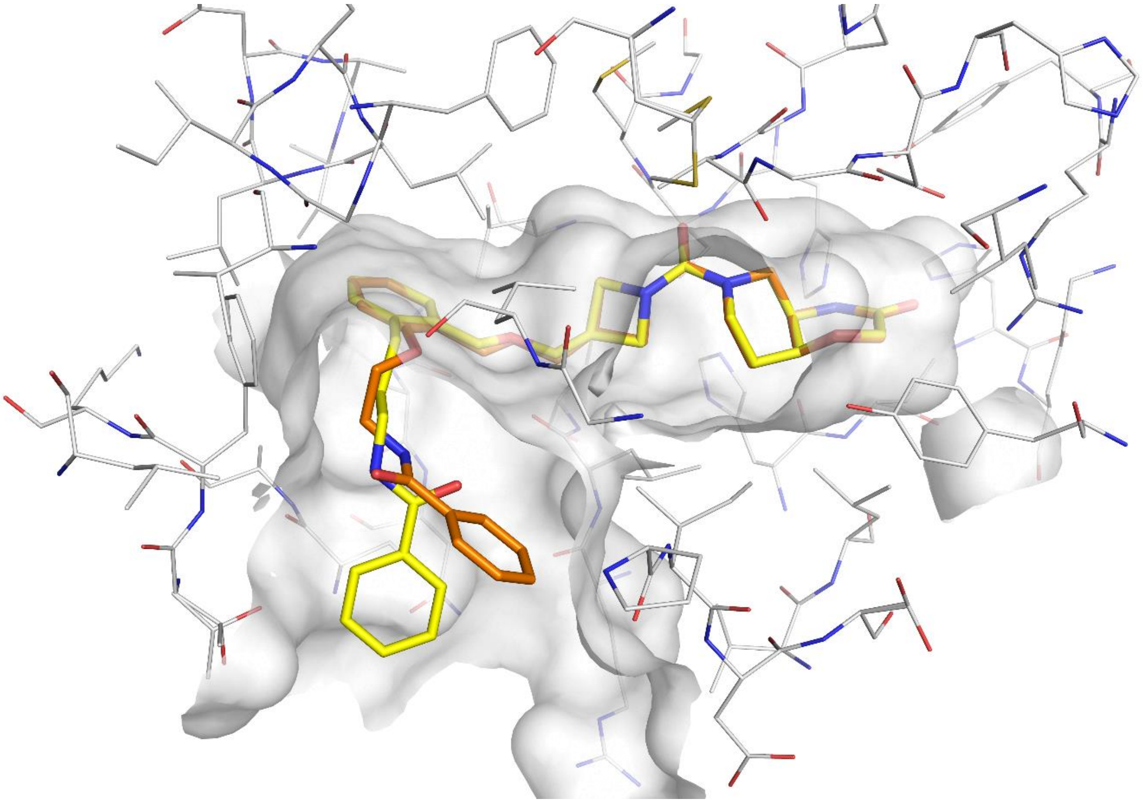

Figure S1. Modeling of MAGL binding pocket with a ligand **3**, as a surrogate for conjugate probes.

Table S1. Initial screening probes. The half-maximal inhibition concentrations IC_{50} of compounds **S1**-**S8** against human and mouse MAGL enzymes

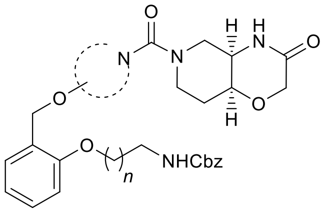

Entry	Compound	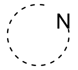	*n*	**IC_{50}** hMAGL (nM)	**IC_{50}** mMAGL (nM)
1	**S1**	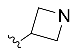	1	70	-
2	**S2 (3)**	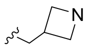	1	2	5
3	**S3**	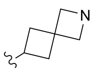	1	11	38
4	**S4**	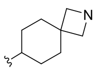	1	15	38
5	**S5**	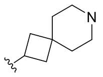	1	7	21
6	**S6**	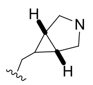	1	97	315
7	**S7**	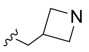	2	3	7
8	**S8**	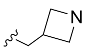	4	46	144

Table S2. Initial Screening Probes The half-maximal inhibition concentrations IC_{50} of compounds with a *meta* exit vector **S9**-**S11** against human and mouse MAGL enzymes

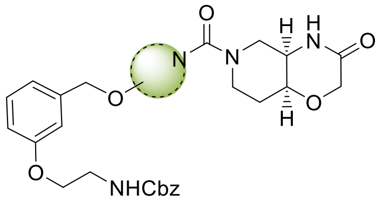

Entry	Compound	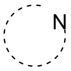	IC_{50} hMAGL (nM)	IC_{50} mMAGL (nM)
1	**S9**	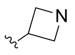	44	38
2	**S10 (2)**	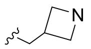	31	97
3	**S11**	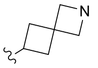	28	76

Compound **S2** was used to test its suitability for the generation of drug-fluorophore conjugates. Following Cbz deprotection of **S2**, fluorophores representatives of the benzoxadiazole, BODIPY, cyanine and rhodamine families were attached. IC_{50} values ranging from single- to double-digit nanomolar for conjugates bearing Nitrobenzoxadiazole (NBD) **S12 (4)**, BODIPY-FL **S13,** and Cyanine-3 (Cy3) **S14** fluorophores (Table S3) showed that the envisioned strategy was suitable and thus confirmed the proof of principle for dye versatility. The exception was conjugate **S15**, bearing a 5(6)-tetramethylrhodamine (TAMRA) fluorophore (Table S3). It was hypothesized that the loss in binding affinity was associated to the presence of a bulky rhodamine group in proximity to the binding pocket. Unlike cyanines or BODIPYs, rhodamine dyes are not typically available from commercial sources with a pre-installed linker for amide coupling. Indeed, a decrease in IC_{50} values was observed when testing TAMRA conjugates containing C3 and C5 linker chains (**S16** and **S17**). Based on these results, C2 alkane linkers were selected for the generation of fluorophore conjugates that involve either low molecular weight fluorophores (such as NBD) or fluorophores that are available from commercial sources with a pre-installed linker (e.g., BODIPYs, cyanines), and C5 alkane linkers were selected for conjugates involving rhodamine derivatives.

Table S3. Dye versatility and linker optimization.

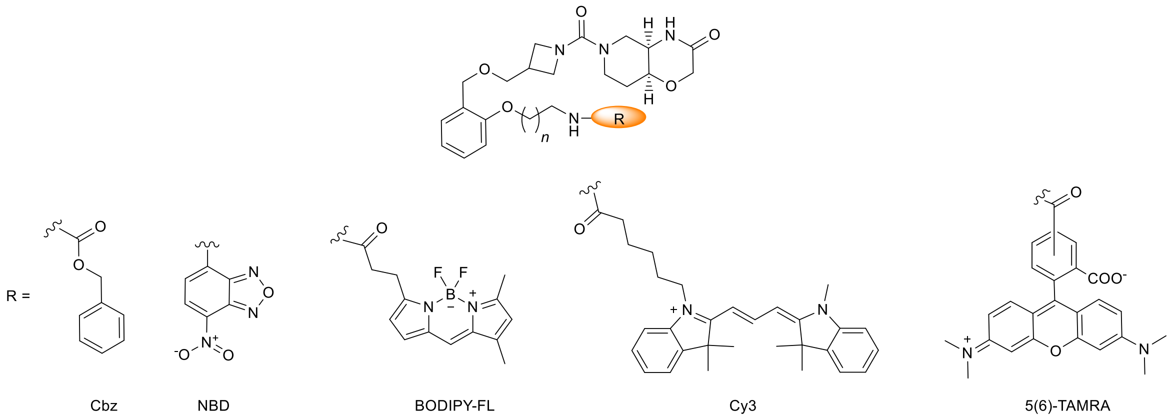

Entry	**Compd.**	**R**	***n***	**IC50 (hMAGL) [nM]**	**IC50 (mMAGL) [nM]**	**λ Absmax / Emmax [nm]**
1	**S2 (3)**	Cbz	1	2	5	**-**
2	**S12 (4)**	NBD	1	3.7	1.7	467/539
3	**S13**	BODIPY-FL	1	8	51	503/512
4	**S14**	Cy3	1	10	31	552/570
5	**S15**	5(6)-TAMRA	1	521	413	544/576
6	**S16**	5(6)-TAMRA	2	215	107	544/576
7	**S17**	5(6)-TAMRA	4	96	117	544/576

Alternative headgroup analogs for **S2** were evaluated (Table S4). Benzoxazinone **S18** (hMAGL IC_{50}: 7.5 nM, entry 2) and carbamate **S19** (hMAGL IC_{50}: 9.2 nM, entry 3) resulted in worsened ligand properties as they showed a slight increase in hMAGL IC_{50} accompanied by a significant difference between human and mouse MAGL. Spiroazetidine derivative **S20** (**5**) afforded subnanomolar IC_{50} values for human, mouse, and cynomolgus monkey MAGL and was therefore selected for further probe optimization.

Table S4. Headgroup analysis

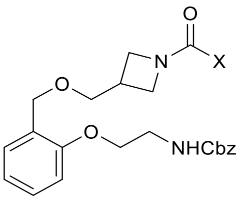

**Entry**	**Compd.**	X	**IC_{50}** hMAGL (nM)	**IC_{50}** mMAGL (nM)	**IC_{50} Cyno** MAGL (nM)
1	**S2 (3)**	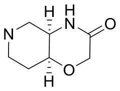	2	5	-
2	**S18**	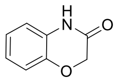	7.5	35	-
3	**S19**	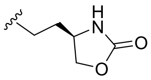	9.2	66	-
4	**S20 (5)**	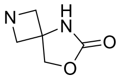	0.29	0.59	0.094

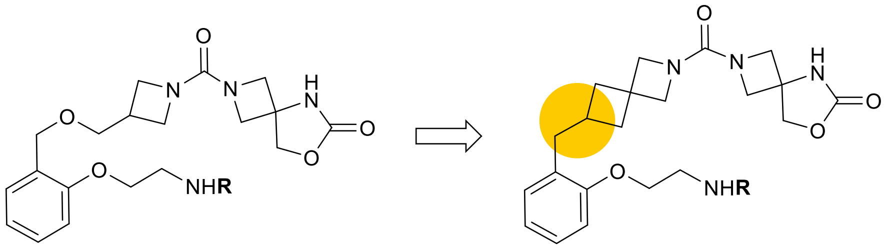

Figure S2. LHS optimization.

Further modifications to the ligand's LHS were implemented, aiming to improve overall probe properties and overcome shortages detected for reference ligand S2, namely chemical instability and flexibility. A modified LHS motif was investigated, in which the benzylic oxygen was absent, and a more rigid spiroazetidine was included (Figure S2). This modification showed an improved affinity for MAGL, affording subnanomolar IC_{50} values for NBD and Cy3 model conjugates even for the HHPO headgroup series (data not shown). All probes, which differ largely in fluorophore structure and properties, afforded IC_{50} values in the 0.1-0.2 nM for human and mouse MAGL, thereby showcasing the robustness of the conjugate platform approach.

Table S5. Binding affinities for optimized reversible MAGL conjugate fluorescent probes.

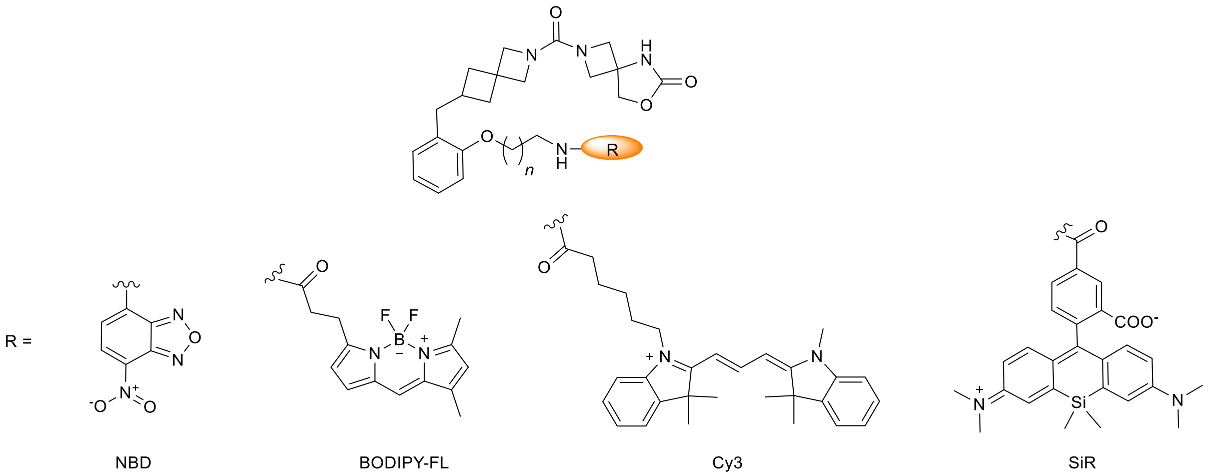

Entry	Compd.	R	*n*	**IC$_{50}$** hMAGL (nM)	**IC$_{50}$** mMAGL (nM)
1	**8**	NBD	1	0.124	0.138
2	**9**	BODIPY-FL	1	0.125	0.107
3	**10**	Si-R	4	0.187	0.155
4	**S21**	Cy3	1	0.122	0.119

2. Synthesis and Characterization of the Probe Molecules

2.1. General Methods

All reagents and solvents were purchased from commercial suppliers and used as received unless otherwise specified. Purifications by flash chromatography were performed using the following equipment: Biotage® Isolera™ SP One with UV detection or Biotage® Selekt™ with UV/Vis detection or Combi*Flash*® NextGen 300+ (Teledyne ISCO) equipped with UV/Vis and ELSD detectors. For the stationary phase, CHROMABOND® Flash RS SiOH cartridges (Macherey-Nagel), or RediSep® Rf Silica cartridges (Teledyne ISCO). Reverse-phase high-performance liquid chromatography (RP-HPLC) was performed using a Gilson PLC-2050 system with UV/visible detection and the option of additional ELSD detection and a Nucleodur 100-7 C18ec (Macherey-Nagel). An ALPHA 2-4 LSC lyophilizer (Christ) was used for freeze-dry processes. Palladium on carbon was removed from reaction mixtures by filtration with Rotilabo syringe filters (Roth) and PTFE filters (pore size: 0.45 µm).

TLC was performed on 0.25 mm Silicagel 60 F_{254} aluminum-supported plates (Merck), with detection via UV light (254 nm) and/or Ceric Ammonium Molybdate (CAM) stain (4.0 g $Ce(NH_4)_4(SO_4)_4$ $2H_2O$; 10 g ammonium molybdate; 40 mL $H_2SO_{4(c)}$; 360 mL H_2O). For monitoring reactions by mass spectrometry, an LC-MS system from Agilent Technologies 6120 series Quadrupole MS linked to an Agilent Technologies HPLC 1290 Infinity series machine with a Thermo Accucore™ RP-MS column (pore size: 2.6 µm; dimension: 30 x 2.1 mm) was used. NMR spectra were obtained using Ultrashield™ 300 (Bruker) spectrometers at 300 MHz (^{1}H) and 75 MHz (^{13}C) or Ascend 600 (Bruker) at 600 MHz (^{1}H) and 150 MHz (^{13}C) or an Ascend 750 (Bruker) at 750 MHz (^{1}H) and 187 MHz (^{13}C). $CDCl_3$, CD_3OD, CD_3CN or D_2O were used as solvents and chemical shifts (δ) referenced to internal standards ($CDCl_3$: 7.26 ppm ^{1}H, 77.16 ppm ^{13}C; CD_3OD: 4.87 or 3.31 ppm ^{1}H, 49.0 ppm ^{13}C; CD_3CN 2.13 ppm ^{1}H. 118.3 ^{13}C, D_2O: 4.79 ppm ^{1}H) unless stated otherwise. Assignments were supported by COSY and HSQC experiments when necessary. All chemical shifts for experiments are reported in ppm (δ). Spin multiplicities were described as singlet (s), duplet (d), triplet (t), quartet (q), and mulitplet (m). All ^{13}C NMR spectra were recorded with 1H-broadband decoupling unless stated otherwise. HRMS measurements were performed on an Agilent Technologies 6530 Accurate Mass Q-ToF LC/MS linked to Agilent Technologies HPLC 1260 Infinity II system.

2.2. Main Text Compounds

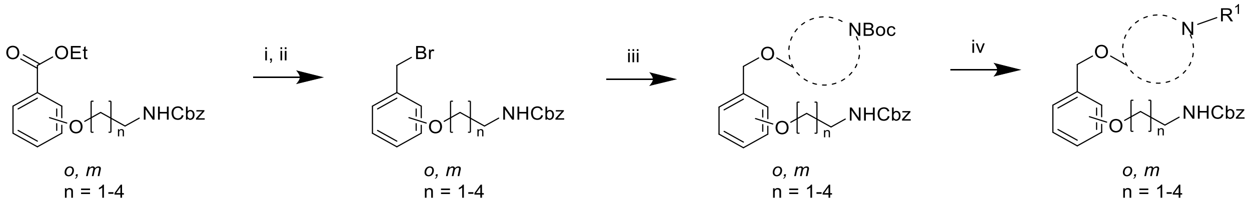

Scheme S1. General synthetic scheme for the synthesis of **3** and analogous compounds.

Benzyl (2-(2-(((1-((4a*R*,8a*S*)-3-oxooctahydro-2*H*-pyrido[4,3-b][1,4]oxazine-6-carbonyl)azetidin-3-yl)methoxy)methyl)phenoxy)ethyl)carbamate (3)

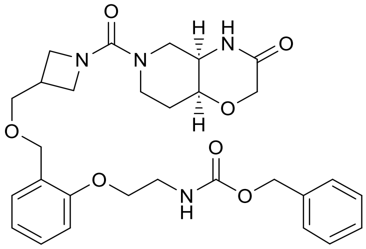

Compound **3** was synthesized according to the general synthetic Scheme S1.

Ethyl 2-(2-(((benzyloxy)carbonyl)amino)ethoxy)benzoate (S22)

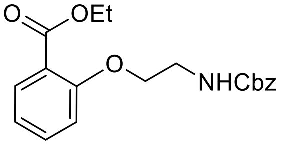

Ethyl salicylate (CAS RN 118-61-6, 2.26 g, 13.6 mmol) was dissolved in DMF (8 mL), Cs_2CO_3 (4.43 g, 13.6 mmol) was added, and the mixture was stirred at room temperature for five minutes. Next, 2-(((benzyloxy)carbonyl)amino)ethyl 4-methylbenzenesulfonate (**15a**, CAS RN 93407-96-6, 2.80 g, 8.00 mmol) was added, and the mixture was stirred at 55 °C for 18 h. The reaction was quenched by diluting with DCM (60 mL), extracted with aqueous NaOH solution (10% m/v) (2 x 40 mL), and washed with brine (50 mL). The organic layer was dried over $MgSO_4$, filtrated, and concentrated. The crude was purified by silica gel flash chromatography (0 to 50% AcOEt in cyclohexanes) to afford the title compound (2.54 g, 7.40 mmol, 92%). ^1H NMR (300 MHz, $CDCl_3$) δ 7.82 (dd, *J* = 7.8, 1.8 Hz, 1H), 7.45 (ddd, *J* = 8.2, 7.4, 1.8 Hz, 1H), 7.41 – 7.24 (m, 5H), 7.07 – 6.90 (m, 2H), 5.98 (s, 1H, N*H*Cbz), 5.12 (s, 2H, OC*H₂*), 4.35 (q, *J* = 7.1 Hz, 2H, OC$H_2$$CH_3$), 4.14 (t, *J* = 5.0 Hz, 2H, OCH_2), 3.64 (m, 2H, CH_2NHCbz), 1.36 (t, *J* = 7.1 Hz, 3H, OCH_2CH_3). ^{13}C NMR (75 MHz, $CDCl_3$) δ 166.2, 158.44, 156.73 (C=O CO_2Et, C=O Cbz, C_{Ar}-O) 136.8, 133.7, 131.9, 128.6, 128.11, 128.09, 121.1, 120.9, 114.3, 68.8 (OCH_2), 66.8 (OCH_2), 61.1 (OCH_2), 40.6 (CH_2NHCbz), 14.4 (CH_3). LC-HRMS (ESI) calc. for $C_{19}H_{21}NO_5Na$ $[M+Na]^+$: 366.1312; found: 366.1301.

Step i Benzyl (2-(2-(hydroxymethyl)phenoxy)ethyl)carbamate (S23)

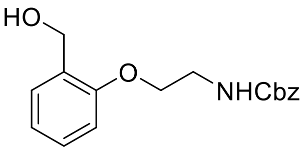

Ester ethyl 2-(2-(((benzyloxy)carbonyl)amino)ethoxy)benzoate (1.15 g, 3.35 mmol) was dissolved in toluene (11.2 mL) under N_2 atmosphere at 0 °C, and a 1M DIBAL in DCM solution (15.1 mL, 15.1 mmol) was added dropwise. The reaction mixture was stirred at 0 °C until completion (1.5 h, TLC monitoring). The reaction was quenched by diluting with DCM (20 mL) and dropwise addition of AcOH (0.86 mL, 15.1 mmol). The mixture was then extracted with 10% aq. sn. NaOH (2 x 40 mL) and washed with brine (40 mL). The organic layer was dried over $MgSO_4$, filtrated, and concentrated. The concentrated was purified by silica gel flash chromatography (0 to 65% AcOEt in cyclohexanes) to afford the title compound (817 mg, 2.71 mmol, 81%). ^{1}H NMR (300 MHz, $CDCl_3$) δ 7.39 – 7.20 (m, 4H), 6.95 (td, *J* = 7.5, 1.1 Hz, 1H), 6.85 (d, *J* = 8.1 Hz, 1H), 5.12 (s, 2H, OC*H₂*), 4.66 (s, 2H, OC*H₂*), 4.10 (t, *J* = 5.0 Hz, 2H, OC*H₂*), 3.63 (t, *J* = 5.0 Hz, 2H, C*H₂*NHCbz). ^{13}C NMR (75 MHz, $CDCl_3$) δ 156.9, 156.7 (C=O Cbz, C_{Ar}-O), 136.5, 129.5, 129.4, 129.3, 128.7, 128.3, 121.3, 111.6, 67.6 (O*C*H_2), 67.1 (O*C*H_2), 62.1 (O*C*H_2), 40.8 (*C*H_2-NHCbz). LC-HRMS (ESI) calc. for $C_{17}H_{20}NO_4$ $[M+H]^+$: 302.1386; found: 302.1365.

Step ii Benzyl (2-(2-(bromomethyl)phenoxy)ethyl)carbamate (S24)

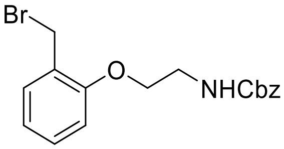

Alcohol *benzyl (2-(2-(hydroxymethyl)phenoxy)ethyl)carbamate* (795 mg, 2.64 mmol) was dissolved in DCM (20.3mL) and cooled to 0 °C. Next, CBr_4 (1.00 g, 3.04 mmol) was added, followed by PPh_3 (796 mg, 3.04 mmol, 1.5 M solution in DCM). The reaction mixture was stirred at r.t. until completion (10 min, TLC monitoring). The reaction was quenched by diluting with DCM (30 mL), extracted with aq. sat. $NaHCO_3$ (2 x 30 mL) and washed with brine (20 mL). The organic layer was dried over $MgSO_4$, filtrated, and concentrated. The concentrated was purified by silica gel flash chromatography (0 to 40% AcOEt in cyclohexanes) to afford the title compound (694 mg, 1.90 mmol, 72%). ^{1}H NMR (300 MHz, $CDCl_3$) δ 7.38 – 7.28 (m, 7H), 7.04 – 6.81 (m, 2H), 5.12 (s, 2H, OC*H₂* Cbz), 4.59 (d, *J* = 27.9 Hz, 2H, C*H₂*-Br), 4.14 (t, *J* = 4.9 Hz, 2H, OC*H₂*CH_2-NHCbz), 3.68 (q, *J* = 5.3 Hz, 2H, OCH_2C*H₂*-NHCbz). ^{13}C NMR (75 MHz, $CDCl_3$) δ 156.7, 136.7, 131.0, 130.6, 128.7, 128.1, 121.2, 111.8, 67.5 (-O*C*H_2-), 66.9 (-O*C*H_2-), 40.7 (-OCH_2*C*H_2-NHCbz), 29.7 (*C*H_2-Br). LC-MS (ESI): 386.0 $[M+Na]^+$.

Step iii tert-Butyl 3-(((2-(2-(((benzyloxy)carbonyl)amino)ethoxy)benzyl)oxy)methyl)azetidine-1-carboxylate (S25)

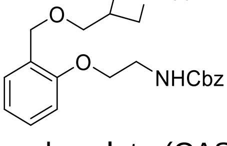

tert-Butyl 3-(hydroxymethyl)azetidine-1-carboxylate (CAS RN 142253-56-3, 196 mg, 1.05 mmol) was dissolved in anhydrous THF (7 mL) at 0 °C, KO^{t}Bu (118 mg, 1.05 mmol) was added, and the

mixture was stirred for 5 min. Next, bromine derivative *benzyl (2-(2-(bromomethyl)phenoxy)ethyl)carbamate* (254.9 mg, 0.70 mmol) was dissolved in anhydrous THF (1.5 mL) and added dropwise to the reaction mixture. The mixture was then allowed to warm up to r.t. and stirred for 4 h until completion (TLC monitoring). The reaction was quenched by diluting with DCM (10 mL) and dropwise addition of AcOH (0.1 mL, 1.05 mmol). The mixture was extracted with aq. sat. $NaHCO_3$ (2 x 20 mL) and washed with brine (20 mL). The organic layer was dried over $MgSO_4$, filtrated, and concentrated. The concentrated was purified by silica gel flash chromatography (0 to 40% AcOEt in cyclohexanes) to afford the title compound (186 mg, 0.40 mmol, 57%). ^{1}H NMR (300 MHz, $CDCl_3$) δ 7.39 – 7.23 (m, 7H), 6.96 (td, *J* = 7.4, 1.0 Hz, 1H), 6.85 (d, *J* = 8.2 Hz, 1H), 5.50 (s, 1H, N*H*Cbz), 5.10 (s, 2H, OC*H₂*), 4.54 (s, 2H, OC*H₂*), 4.14 – 4.03 (m, 2H, OC*H₂*), 3.86 (t, *J* = 8.4 Hz, 2H, OC*H₂*), 3.67 – 3.51 (m, 6H, C*H₂*NHCbz, 2 x N-CH_2 azetidine), 2.78 – 2.63 (m, 1H, C*H* azetidine), 1.43 (s, 9H, 3 x C*H₃* Boc). 13C NMR (75 MHz, CDCl3) δ 156.5, 136.5, 129.9, 129.4, 128.7, 128.44, 128.38, 126.7, 121.2, 112.1, 79.4 (*C*(CH_3) Boc), 72.7 (C*H* azetidine), 68.8 (O*C*H_2), 67.6 (O*C*H_2), 67.0 (O*C*H_2), 40.8 (*C*H_2-NHCbz), 28.5 (*C*H_3 Boc). LC-HRMS (ESI) calc. for $C_{26}H_{35}N_2O_6$ $[M+H]^+$: 471.2490; found: 471.2475.

Step iv Benzyl (2-(2-(((1-((4a*R*,8a*S*)-3-oxooctahydro-2H-pyrido[4,3-b][1,4]oxazine-6-carbonyl)azetidin-3-yl)methoxy)methyl)phenoxy)ethyl)carbamate (3)

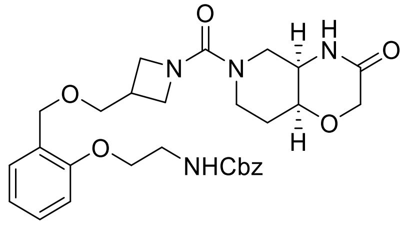

N-Boc protected derivative ***S25*** (93.0 mg, 0.20 mmol) was dissolved in DCM (1.3 mL) at 0 °C and TFA (152 µL, 2.0 mmol) was added. The reaction mixture was stirred at that temperature until Boc deprotection was complete (1 h, LC-MS monitoring). The reaction was diluted with toluene (3.00 mL) and co-evaporated with toluene (3.00 mL) under reduced pressure twice. The 2,2,2-trifluoroacetate salt of the free amine 3-(((2-(2-(((benzyloxy)carbonyl)amino)ethoxy)benzyl)oxy)methyl)azetidine was used in the next step without further purification.

(4a*R*,8a*S*)-3-Oxooctahydro-2*H*-pyrido[4,3-b][1,4]oxazin-6-ium (2*S*,3*S*)-3-carboxy-2,3-bis((4-methylbenzoyl)oxy)propanoate salt (CAS RN 2624363-49-9, 119 mg, 0.16 mmol) was dissolved in anhydrous acetonitrile (0.7 mL) and1,1′-carbonyl-di-(1,2,4-triazole) (CAS RN 41864-22-6, 26.3 mg, 0.16 mmol), followed by triethylamine (0.16 mL, 1.12 mmol) were added. The reaction mixture was stirred at room temperature for 2 h. Then, the amine nucleophile from step 1 2,2,2-trifluoroacetate salt of 3-(((2-(2-(((benzyloxy)carbonyl)amino)ethoxy)benzyl)oxy)methyl)azetidine was dissolved in ACN (0.5 mL) and added dropwise to the reaction mixture. The reaction mixture was heated to 50 °C and stirred at that temperature for 2.5 h. The reaction was quenched by diluting with DCM (5.00 mL), extracted with aq. sat. $NaHCO_3$ (2.00 mL) and washed with brine (2.00 mL). The organic layer was dried over MgSO4, filtrated, and concentrated. The concentrated was purified by HPLC (25 to 75% ACN in H2O with 0.1% TFA) to afford the title compound (70.0 mg, 0.13 mmol, 79%). ^{1}H NMR (300 MHz, MeOD) δ 7.37 – 7.16 (m, 7H, H-Ar), 6.96 – 6.84 (m, 2H, H-Ar), 5.05 (s, 2H, O-C*H₂*-), 4.51 (s, 2H, O-C*H₂*-), 4.25 – 4.06 (m, 2H), 4.06

– 3.88 (m, 4H), 3.82 – 3.62 (m, 3H), 3.58 – 3.45 (m, 4H), 3.32 – 3.20 (m, 3H), 2.99 – 2.83 (m, 2H), 2.80 – 2.66 (m, 1H, -C*H*- azetidine), 1.90 – 1.64 (m, 2H. –C*H₂*- HHPO). ^{13}C NMR (75 MHz, MeOD) δ 171.2, 163.7 (2 x C=O urea, amide), 158.9, 158.0 (C=O Cbz, C_{ipso} phenol), 138.3, 130.8, 130.3, 129.5, 129.0, 128.8, 127.8, 121.8, 112.9 (C-Ar), 73.0, 70.4, 69.18, 68.21, 68.1, 67.5, 55.2, 55.1, 50.4, 47.1, 41.5, 40.5, 30.4, 30.2. LC-HRMS (ESI) calc. for $C_{29}H_{37}N_4O_7^+$ [M+H]$^+$: 553.2657; found: 553.2665.

(4a*R*,8a*S*)-6-(3-((Benzyloxy)methyl)azetidine-1-carbonyl)hexahydro-2*H*-pyrido[4,3-b][1,4]oxazin-3(4H)-one (1)

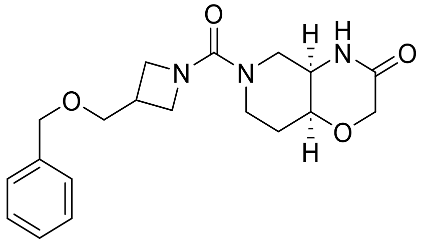

The synthetic procedure described for **3** was followed, starting from *tert-butyl 3-((benzyloxy)methyl)azetidine-1-carboxylate (CAS RN* 1373233-17-0, 28 mg, 0.1 mmol), to afford the title compound **1** (15.7 mg, 0.044 mmol, 44%). ^{1}H NMR (300 MHz, $CDCl_3$) δ 7.40 – 7.27 (m, 5H), 6.41 (s, 1H), 5.30 (s, 1H), 4.53 (s, 2H), 4.24 (dd, *J* = 18.3, 16.8 Hz, 2H), 4.08 (t, *J* = 8.4 Hz, 1H), 4.01 (t, *J* = 8.4 Hz, 1H), 3.98 – 3.93 (m, 1H), 3.88 (dd, *J* = 13.0, 4.9 Hz, 1H), 3.74 (ddd, *J* = 19.0, 8.3, 5.5 Hz, 2H), 3.59 (d, *J* = 6.7 Hz, 2H), 3.53 – 3.43 (m, 1H), 3.42 – 3.31 (m, 0H), 3.04 (dt, *J* = 13.0, 10.1 Hz, 2H), 2.93 – 2.73 (m, 1H), 1.97 – 1.69 (m, 2H). LC-HRMS (ESI) calc. for $C_{19}H_{25}N_3O_4$ [M+H]$^+$: 359.1874 ; found: 359.1845.

Benzyl (2-(3-(((1-((4a*R*,8a*S*)-3-oxooctahydro-2*H*-pyrido[4,3-b][1,4]oxazine-6-carbonyl)azetidin-3-yl)methoxy)methyl)phenoxy)ethyl)carbamate (2)

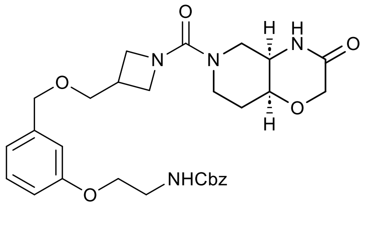

The synthetic procedure described for **3** was followed, starting from *N*-Boc protected derivative **BB10** (see below, building block synthesis), to afford target product **2** (5.0 mg, 9.0µmol, 12%). ^{1}H NMR (300 MHz, MeOD) δ 7.39 – 7.15 (m, 6H), 6.96 – 6.74 (m, 3H), 5.07 (s, 2H, OC*H₂*), 4.48 (s, 2H, OC*H₂*), 4.28 – 3.90 (m, 7H), 3.88 – 3.68 (m, 3H), 3.62 – 3.41 (m, 5H), 3.33 – 3.29 (m, 1H, C*H*NH(C=O) HHPO), 3.05 – 2.88 (m, 2H), 2.87 – 2.68 (m, 1H, C*H* azetidine), 1.94 – 1.64 (m, 2H, C*H₂* HHPO). ^{13}C NMR (75 MHz, MeOD) δ 171.2, 163.7, 160.4, 159.0 (C=O urea, C=O amide, C=O Cbz, C_{Ar}-O), 141.3, 138.4, 130.5, 129.5, 129.0, 128.8, 121.3, 114.93, 114.86, 74.0, 72.6, 70.5 (C*H*-O HHPO), 68.2, 67.8, 67.47, 55.1, 55.1, 50.4 *C*HNH(C=O) HHPO), 47.1, 41.5, 40.6,

30.4 (*C*H azetidine, *C*H_2 HHPO). LC-HRMS (ESI) calc. for $C_{29}H_{36}N_4O_7Na$ $[M+Na]^+$: 575.2476; found: 575.2507.

Benzyl (2-(2-(((1-(6-Oxo-7-oxa-2,5-diazaspiro[3.4]octane-2-carbonyl)azetidin-3-yl)methoxy)methyl)phenoxy)ethyl)carbamate (5)

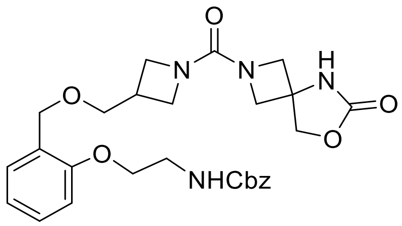

6-Oxo-7-oxa-2,5-diazaspiro[3.4]octan-2-ium 4-methylbenzenesulfonate salt (24.0 mg, 80 µmol) was suspended in acetonitrile (0.40 mL) and TEA (78.0 µL, 0.56 mmol) was added under stirring. After obtaining a clear solution, 1,1′-Carbonyl-di-(1,2,4-triazole) (CDT, 13.1 mg, 8.0 µmol) was added, and the mixture was stirred at room temperature for 2 h. **S25** was subjected to *N*-Boc deprotection as described above. The respective amine TFA salt (0.20 mL, 46.5 mg, 96 µmol), dissolved in ACN, was added to the mixture and heated to 50 °C for 2.5 h. The reaction was quenched by diluting with DCM (6.0 mL) and extracted with water (2.0 mL). The aqueous layer was reextracted with DCM (2 x 3.0 mL), and the organic layers were combined, dried over MgSO4, filtrated, and concentrated. The solvent was removed *in vacuo,* and the remaining oil was purified through RP-HPLC 15-85% ACN in H_2O with 0.1% TFA. The target product was obtained after solvent removal via lyophilization (15.0 mg, 2.9 µmol, 36%). ^{1}H NMR (300 MHz, MeOD) δ 7.36 – 7.18 (m, 7H), 6.96 – 6.84 (m, 2H), 5.07 (s, 2H, O-C*H₂*-), 4.52 (s, 2H, O-C*H₂*-), 4.49 (s, 2H, O-C*H₂*-), 4.08 – 3.98 (m, 6H), 3.95 – 3.85 (m, 2H), 3.65 (dd, *J* = 8.3, 5.4 Hz, 2H), 3.57 – 3.46 (m, 4H), 2.84 – 2.67 (m, 1H, C*H* azetidine). ^{13}C NMR (151 MHz, MeOD) δ 163.8, 160.5, 158.9, 158.1 (2 x C=O carbamate, C=O urea, C_{ipso} phenol), 138.4, 130.7, 130.3, 129.5, 129.0, 128.80, 127.9, 121.8, 112.9 (C-Ar), 75.7, 72.7, 69.2, 68.2, 67.5, 63.8, 55.9, 53.9, 41.56 (*C*H_2-NH-), 30.5 (*C*H azetidine). LC-HRMS (ESI) calc. for $C_{27}H_{33}N_4O_7$ $[M+H]^+$: 525.2344; found: 525.2373.

2-(3-(((2-(2-((7-Nitrobenzo[c][1,2,5]oxadiazol-4-yl)amino)ethoxy)benzyl)oxy)methyl)azetidine-1-carbonyl)-7-oxa-2,5-diazaspiro[3.4]octan-6-one (6)

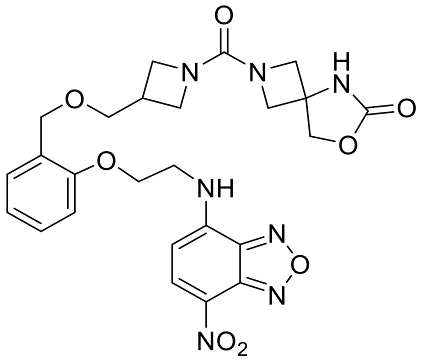

The starting material (12.0 mg, 0.03 mmol) was dissolved in MeOH (0.75 mL), and trimethylamine (13.0 µL, 0.09 mmol) was added. Next, a solution of NBD-fluoride (11.0 mg, 0.06 mmol) in MeOH (0.3 mL) was added dropwise. The reaction was stirred in the dark for 3.5 h. Next, the MeOH was removed in vacuo at 20 °C and the reaction was purified through RP-HPLC 15-85% ACN in H_2O with 0.1% TFA to afford the title product (1.3 mg ,13%). ^{1}H NMR (600 MHz, MeOD) δ 8.55 (t, J = 4.5 Hz, 1H), 7.26 (ddd, J = 9.6, 7.5, 1.8 Hz, 2H), 7.01 (d, J = 8.1 Hz, 1H), 6.93 (td, J = 7.4, 1.0 Hz, 1H), 6.57 (d, J = 8.9 Hz, 1H), 4.59 (s, 2H), 4.42 (s, 2H), 4.34 (t, J = 5.1 Hz, 2H), 4.09 (d, J = 9.2 Hz, 2H), 4.07 (d, J = 9.3 Hz, 2H), 3.89 (t, J = 8.3 Hz, 2H), 3.57 (dd, J = 8.3, 5.4 Hz, 2H), 3.40 (d, J = 6.3 Hz, 2H), 2.73 – 2.63 (m, 1H), 1.35 – 1.30 (m, 2H). LC-HRMS (ESI) calc. for $C_{25}H_{27}N_7O_8Na$ $[M+Na]^+$: 576.1813; found: 576.1832.

1,1,1,3,3,3-Hexafluoropropan-2-yl 3-(((2-(2-(((benzyloxy)carbonyl)amino)ethoxy)benzyl)oxy)methyl)azetidine-1-carboxylate (7)

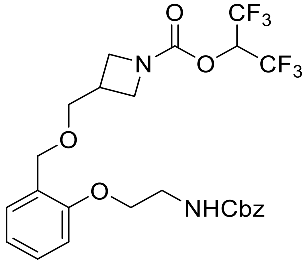

^{1}H NMR (300 MHz, $CDCl_3$) δ 7.39 – 7.27 (m, 7H), 6.97 (td, J = 7.5, 1.1 Hz, 1H), 6.86 (d, J = 8.6 Hz, 1H), 5.64 (hept, J = 6.2 Hz, 1H), 5.47 (s, 1H), 5.10 (s, 2H), 4.55 (s, 2H), 4.09 (t, J = 5.1 Hz, 2H), 3.95 (q, J = 8.9 Hz, 2H), 3.73 (d, J = 5.8 Hz, 2H), 3.62 (q, J = 5.1 Hz, 2H), 3.56 (d, J = 6.7 Hz, 2H), 2.86 – 2.72 (m, 1H). ^{13}C NMR (75 MHz, $CDCl_3$) δ 156.90, 156.87, 152.13, 136.73, 130.30, 129.91, 129.05, 128.90, 128.80, 126.66, 121.53, 112.37, 77.69, 72.03, 69.39, 68.42, 67.96, 67.86, 67.50, 53.11, 52.48, 41.05, 29.57. LC-HRMS (ESI) calc. for $C_{25}H_{27}F_6N_2O_6$ $[M+H]^+$: 565.1768; found: 565.1771.

Benzyl (2-(2-bromophenoxy)ethyl)carbamate (16a)

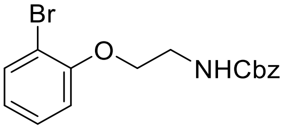

2-Bromophenol (5 g, 1.15 equiv.) was dissolved in acetone (100 mL), K_2CO_3 (6.95 g, 2 equiv.), and benzyltriethylammonium chloride (0.29 g, 5 mol%) were added, and the mixture was stirred at room temperature for five minutes. Next, 2-(((benzyloxy)carbonyl)amino)ethyl 4-methylbenzenesulfonate (8.78 g, 1.0 equiv.) was added, and the mixture was stirred at reflux for 18 h. After cooling, the mixture was carefully concentrated under reduced pressure and the residue was taken up in DCM (60 mL), extracted with aqueous NaOH solution (10% m/v) (2 x 40 mL), and washed with brine (50 mL). The organic layer was dried over $MgSO_4$, filtrated, and concentrated. The crude was purified by silica gel flash chromatography with gradient elution of 0% to 60% EtOAc in cyclohexane to give the title compound as a colorless oil (8.3 g, 94%). ^{1}H NMR (300 MHz, $CDCl_3$) δ 7.47 (dd, J = 7.9, 1.6 Hz, 1H), 7.33 – 7.13 (m, 6H), 6.87 – 6.73 (m, 2H), 5.31 (s, 1H, N*H*Cbz), 5.06 (s, 2H, OC*H₂*), 4.03 (t, J = 5.0 Hz, 2H, OC*H₂*), 3.59 (q, J = 5.4 Hz, 2H,

CH_2NHCbz). ^{13}C NMR (75 MHz, $CDCl_3$) δ 156.6, 154.9 (C=O Cbz, C_{Ar}-O), 136.5, 133.5, 128.7, 128.7, 128.29, 128.26, 122.6, 113.7, 112.5, 68.5 (OCH_2), 67.0 (OCH_2), 40.6 (CH$_2$NHCbz). LC-HRMS (ESI) Calc for $C_{16}H_{16}BrNO_3Na$ [M+Na]$^+$: 372.0206; found: 372.0209.

Benzyl (5-(2-bromophenoxy)pentyl)carbamate (16b)

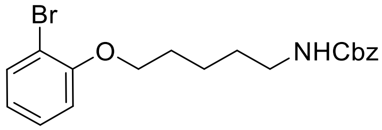

2-Bromophenol (8.5 g, 1.2 equiv.) was dissolved in acetone (200 mL), K_2CO_3 (11.3 g, 2 equiv.), and benzyltriethylammonium chloride (0.5 g, 5 mol%) were added, and the mixture was stirred at room temperature for five minutes. Next, 5-(((benzyloxy)carbonyl)amino)pentyl 4-methylbenzenesulfonate (16 g, 1.0 equiv.) was added, and the mixture was stirred at reflux for 18 h. After cooling the mixture was carefully concentrated under reduced pressure and the residue was taken up in DCM (60 mL), extracted with aqueous NaOH solution (10% m/v) (2 x 40 mL), and washed with brine (50 mL). The organic layer was dried over $MgSO_4$, filtrated, and concentrated. The crude was purified by silica gel flash chromatography with gradient elution 0% to 60% EtOAc in cyclcohexane to give the title compound as a colorless oil (13.14 g, 82%). ^{1}H NMR (300 MHz, $CDCl_3$) δ 7.52 (dd, J = 7.9, 1.6 Hz, 1H), 7.39 – 7.20 (m, 6H), 6.94 – 6.71 (m, 2H), 5.10 (s, 2H, OCH_2), 4.78 (s, 1H, NHCbz), 4.01 (t, J = 6.2 Hz, 2H, OCH_2), 3.24 (q, J = 6.3 Hz, 2H, CH_2NHCbz), 1.86 (t, J = 6.8 Hz, 2H, CH_2 linker), 1.76 – 1.48 (m, 4H, 2 x CH_2 linker). ^{13}C NMR (75 MHz, $CDCl_3$) δ 156.4, 155.3 (C=O Cbz, C_{Ar}-O), 136.6, 133.3, 128.5, 128.4, 128.1, 121.8, 113.2, 112.3, 68.8 (OCH_2), 66.7 (OCH_2), 41.0 (CH$_2$NHCbz), 29.7 (CH$_2$ linker), 28.7 (CH$_2$ linker), 23.3 (CH$_2$ linker). LC-HRMS (ESI) calc. for $C_{19}H_{23}BrNO_3$ [M+H]$^+$: 392.0856; found: 392.0860.

***tert*-Butyl 6-((4,4,5,5-tetramethyl-1,3,2-dioxaborolan-2-yl)methylene)-2-azaspiro[3.3]heptane-2-carboxylate (17)**

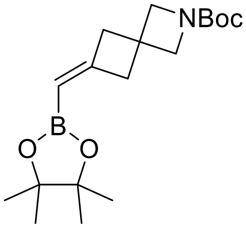

In an oven-dried flask, tetramethylpiperidine (1.25 mL, 7.40 mmol) was dissolved in anhydrous THF (37.0 mL) and cooled to -78 °C under N_2 atmosphere. A solution of nBuLi 2.5 M in THF (3.00 mL, 7.40 mmol) was added dropwise, and the reaction was stirred at the same temperature for 30 min. Next, a 0.84 M solution of bis((pinacolato)boryl)methane (CAS RN 78782-17-9) in THF (8.83 mL, 7.40 mmol) was added dropwise. The reaction was stirred for 5 min, and then a 0.20 M solution of ketone in anhydrous THF (18.5 mL, 3.70 mmol) was added dropwise over 5 min. The reaction mixture was slowly allowed to warm up to room temperature overnight. Upon completion, the reaction was opened to air and filtered through a silica plug eluting with Et_2O. The mixture was concentrated under reduced pressure and purified by silica gel chromatography 0 to 20% EtOAc in cyclohexane with ELSD detection to afford the title compound in quantitative yield. ^{1}H NMR (300 MHz, $CDCl_3$) δ 5.18 (p, J = 2.3 Hz, 1H), 4.00 – 3.87 (m, 4H), 3.10 – 3.05 (m, 2H), 2.93 (dq, J = 3.3, 1.9 Hz, 2H), 1.43 (s, 9H), 1.23 (s, 12H). ^{13}C NMR (75 MHz, $CDCl_3$) δ 161.76, 156.40,

83.15, 82.87, 79.43, 45.50, 45.16, 33.27, 28.54, 24.99, 24.86. LC-HRMS (ESI) calc. for $C_{18}H_{31}BNO_4$ $[M+H]^+$: 336.2340; found: 336.2342.

tert-Butyl 6-(2-(2-(((benzyloxy)carbonyl)amino)ethoxy)benzylidene)-2-azaspiro[3.3]heptane-2-carboxylate (18a)

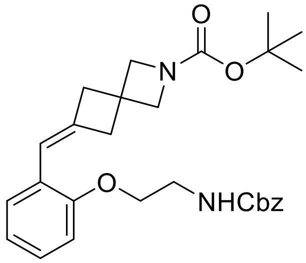

In a closed vial under a nitrogen atmosphere, *tert-butyl 6-((4,4,5,5-tetramethyl-1,3,2-dioxaborolan-2-yl)methylene)-2-azaspiro[3.3]heptane-2-carboxylate* (117 mg, 0.35 mmol), **16a** (123 mg, 0.35 mmol) and Na_2CO_3 (74.2 mg, 0.70 mmol) were suspended in a dioxane/water 3:1 mixture (2.30 mL) previously purged by bubbling N_2 with sonication. The mixture was purged with N_2 bubbling for 10 min. Then, Pd(dppf)Cl_2 (CAS RN 72287-26-4) was added, and the mixture was purged for an additional 15 min. The reaction was warmed up to 55 °C and stirred overnight at this temperature. The reaction was then diluted with DCM (15 mL) and extracted with aq. sat. $NaHCO_3$ (20 mL) and washed with brine (20 mL). The organic layer was dried over $MgSO_4$, concentrated under reduced pressure, and purified by silica gel chromatography 0 to 60% EtOAc in cyclohexane to afford the title compound (97.0 mg, 0.20 mmol, 58%). ^{1}H NMR (300 MHz, $CDCl_3$) δ 7.41 – 7.29 (m, 5H), 7.24 – 7.08 (m, 2H), 6.92 (t, *J* = 7.4 Hz, 1H), 6.82 (d, *J* = 8.2 Hz, 1H), 6.45 (t, *J* = 2.4 Hz, 1H), 5.27 – 5.17 (m, 1H), 5.12 (s, 2H), 4.05 (t, *J* = 5.1 Hz, 2H), 3.97 (s, 3H), 3.64 (q, *J* = 5.5 Hz, 2H), 3.15 (s, 2H), 3.04 (s, 2H), 1.44 (s, 9H). ^{13}C NMR (75 MHz, $CDCl_3$) δ 156.83, 156.71, 155.31, 136.94, 136.77, 129.05, 128.72, 128.69, 128.02, 127.83, 126.77, 121.42, 116.83, 112.32, 79.83, 77.69, 67.87, 67.39, 44.10, 43.91, 41.13, 35.14, 28.86, 27.37. LC-HRMS (ESI) calc. for $C_{28}H_{34}N_2O_5Na$ $[M+Na]^+$: 501.2360; found: 501.2388.

Benzyl (2-(2-((2-(6-oxo-7-oxa-2,5-diazaspiro[3.4]octane-2-carbonyl)-2-azaspiro[3.3]heptan-6-ylidene)methyl)phenoxy)ethyl)carbamate (19a)

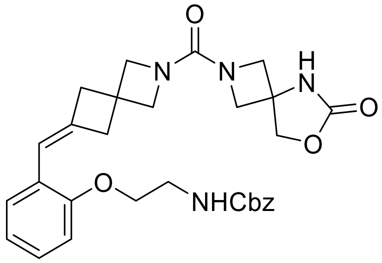

Following the synthetic procedure described for **3**, **18a** was subjected to Boc deprotection followed by carbamate headgroup coupling with 7-oxa-2,5-diazaspiro[3.4]octan-6-one as a tosylate salt (49.3 mg, 46%). ^{1}H NMR (300 MHz, CD_3CN) δ 7.39 – 7.27 (m, 5H), 7.21 (d, *J* = 7.7 Hz, 1H), 7.14 (t, *J* = 7.8 Hz, 1H), 6.91 (t, *J* = 7.2 Hz, 2H), 6.49 (d, *J* = 13.8 Hz, 2H), 5.88 (s, 1H), 5.07 (s, 2H), 4.46 (s, 2H), 4.07 – 3.95 (m, 6H), 3.93 (s, 4H), 3.49 (q, *J* = 5.6 Hz, 2H), 3.15 (s, 2H),

3.00 (s, 2H). ^{13}C NMR (75 MHz, CD_3CN) δ 163.18, 158.42, 157.47, 156.15, 138.36, 137.73, 129.45, 128.86, 128.67, 128.50, 128.17, 127.33, 121.62, 117.10, 113.07, 75.09, 68.14, 66.87, 63.54, 62.82, 55.20, 44.20, 44.00, 41.26, 36.10. LC-HRMS (ESI) calc. for $C_{29}H_{33}N_4O_6$ $[M+H]^+$: 533.2395; found: 533.2371.

***tert*-Butyl 6-(2-((5-(((benzyloxy)carbonyl)amino)pentyl)oxy)benzylidene)-2-azaspiro[3.3]heptane-2-carboxylate (18b)**

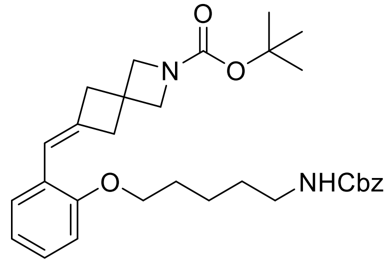

In analogy to the procedure described for **18a**, benzyl (5-(2-bromophenoxy)pentyl)carbamate **16b** was reacted with **17** to afford **18b** (800 mg, 77%).^{1}H NMR (300 MHz, $CDCl_3$) δ 7.40 – 7.28 (m, 5H), 7.22 – 7.08 (m, 2H), 6.94 – 6.85 (m, 1H), 6.83 (d, *J* = 8.2 Hz, 1H), 6.53 – 6.45 (m, 1H), 5.10 (s, 2H), 4.83 – 4.71 (m, 1H), 3.96 (d, *J* = 4.7 Hz, 6H), 3.23 (q, *J* = 6.5 Hz, 2H), 3.16 (s, 2H), 3.04 (s, 2H), 1.83 (p, *J* = 6.7 Hz, 2H), 1.66 – 1.47 (m, 5H), 1.44 (s, 9H). ^{13}C NMR (75 MHz, $CDCl_3$) δ 156.54, 156.40, 155.54, 136.71, 136.08, 128.66, 128.27, 127.58, 127.35, 126.42, 120.45, 116.76, 111.82, 79.49, 77.37, 68.06, 66.79, 61.46, 43.79, 43.63, 41.10, 34.81, 29.90, 29.06, 28.54, 23.49. LC-HRMS (ESI) calc. for $C_{31}H_{41}N_2O_5$ $[M+H]^+$: 521.3010; found: 521.3000.

Benzyl (5-(2-((2-(6-oxo-7-oxa-2,5-diazaspiro[3.4]octane-2-carbonyl)-2-azaspiro[3.3]heptan-6-ylidene)methyl)phenoxy)pentyl)carbamate (19b)

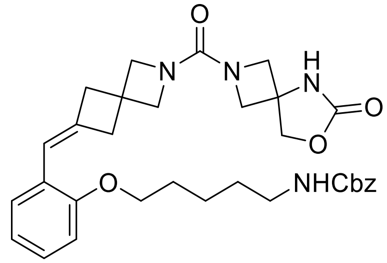

18b was subjected to *N*-Boc deprotection followed by carbamate headgroup coupling with 7-oxa-2,5-diazaspiro[3.4]octan-6-one as a tosylate salt (35.8 mg, 42%). ^{1}H NMR (300 MHz, $CDCl_3$) δ 7.39 – 7.28 (m, 5H), 7.14 (ddt, *J* = 6.1, 4.4, 1.9 Hz, 2H), 6.88 (t, *J* = 7.4 Hz, 1H), 6.82 (d, *J* = 8.7 Hz, 1H), 6.50 (t, *J* = 2.4 Hz, 1H), 5.09 (s, 2H), 4.83 (s, 1H), 4.49 (s, 2H), 4.12 (d, *J* = 9.3 Hz, 2H), 4.03 – 3.90 (m, 8H), 3.22 (q, *J* = 6.5 Hz, 2H), 3.15 (s, 2H), 3.04 (s, 2H), 2.26 (s, 2H), 1.82 (p, *J* = 6.1 Hz, 2H), 1.54 (ddt, *J* = 15.0, 10.5, 5.4 Hz, 4H). ^{13}C NMR (75 MHz, $CDCl_3$) δ 162.20, 157.73, 155.45, 134.81, 128.54, 128.17, 128.10, 127.65, 127.25, 126.05, 120.32, 117.13, 111.72, 77.20, 74.60, 67.92, 66.70, 62.86, 62.36, 54.46, 43.74, 43.55, 40.98, 35.46, 29.71, 28.88, 23.35. LC-HRMS (ESI) calc. for $C_{32}H_{39}N_4O_6$ $[M+H]^+$: 576.2896; found: 576.2889.

1,1,1,3,3,3-Hexafluoropropan-2-yl 6-(2-(2-(((benzyloxy)carbonyl)amino)ethoxy)benzylidene)-2-azaspiro[3.3]heptane-2-carboxylate (19c)

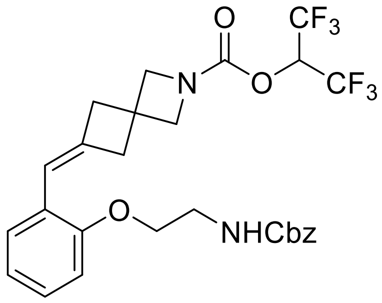

Building block **19a** was subjected to the standard *N*-Boc deprotection procedure (see above). The crude free amine, as TFA salt (148 mg, 0.30 mmol), was dissolved in anhydrous DCM (2 mL), and DIPEA (0.12 mL, 0.69 mmol, 1.2 equiv.) was added. The mixture was cooled to 0 °C before a 50% wt $(CF_3O)_2CO$ solution in Et_2O (prepared as previously described[1]) (239 mg, 0.33 mmol, 1.1 equiv.) was added. The mixture was allowed to warm to room temperature and stirred for 2 h. The mixture was diluted with DCM (10 mL) and washed with brine (5 mL). The organic layer was dried over $MgSO_4$ and purified by HPLC with 35 to 85 % ACN with 0.1% TFA. The solvents were removed via lyophilization to afford compound **20c** (60 mg, 0.105 mmol, 35%). ^{1}H NMR (600 MHz, $CDCl_3$) δ 7.40 – 7.30 (m, 5H), 7.16 (t, *J* = 7.5 Hz, 2H), 6.93 (t, *J* = 7.5 Hz, 1H), 6.84 (d, *J* = 8.4 Hz, 1H), 6.49 (t, *J* = 2.6 Hz, 1H, Ar-C*H*=C), 5.65 (hept, *J* = 6.2 Hz, 1H, OC*H*$(CF_3)_2$), 5.18 (s, 1H, N*H*Cbz), 5.12 (s, 2H, OC*H*$_2$), 4.15 (m, 4H, 2 x N-C*H*$_2$- spirocycle), 4.06 (t, *J* = 5.2 Hz, 2H, OC*H*$_2$), 3.64 (m, 2H, C*H*$_2$NHCbz), 3.21 (s, 2H, C-C*H*$_2$- spirocycle), 3.10 (s, 2H, C-C*H*$_2$- spirocycle). ^{13}C NMR (151 MHz, $CDCl_3$) δ 156.5, 155.1, 151.7 (2 x C=O carbamate, *C*$_{Ar}$-O), 136.5, 135.1, 128.8, 128.43, 128.38, 128.0, 127. 6, 126.3, 121.8, 120.81 (q, *J* = 283.0 Hz, *C*F_3), 117.2 (Ar-*C*H=C), 112.2, 68.49 – 67.24 (m, O*C*H-$(CF_c)_2$), 67.6 (O*C*H_2), 67.1 (O*C*H_2), 62.2 (N-*C*H_2- spirocycle), 61.5 (N-*C*H_2- spirocycle), 43.7 (C-*C*H_2- spirocycle), 43.5 (C-*C*H_2- spirocycle), 40.8 (C*H*$_2$NHCbz), 35.7 (-*C*- spirocycle). LC-HRMS (ESI) calc. for $C_{27}H_{27}F_6N_2O_5$ $[M+H]^+$: 573.1819; found: 573.1835

1,1,1,3,3,3-Hexafluoropropan-2-yl 6-(2-((5-(((benzyloxy)carbonyl)amino)pentyl)oxy)benzylidene)-2-azaspiro[3.3]heptane-2-carboxylate (19d)

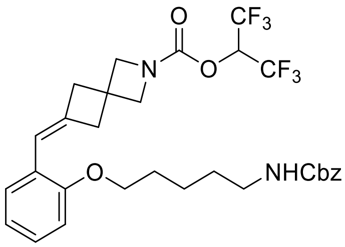

In analogy to the procedure described above, **19b** was subjected to *N*-Boc deprotection and reacted with 50% wt $(CF_3O)_2CO$ solution in Et_20 to afford compound **20d** (81 mg, 0.14 mmol, 47%). ^{1}H NMR (300 MHz, $CDCl_3$) δ 7.36 – 7.19 (m, 5H), 7.16 – 6.99 (m, 2H), 6.85 – 6.66 (m, 2H), 6.44 (t, *J* = 2.4 Hz, 1H, C*H* vinyl), 5.57 (p, *J* = 6.2 Hz, 1H, OC*H*$(CF_3)_2$), 5.01 (s, 2H, OC*H*$_2$), 4.10

– 3.97 (m, 4H), 3.87 (t, *J* = 6.4 Hz, 2H), 3.21 – 3.08 (m, 4H), 3.00 (d, *J* = 3.7 Hz, 2H), 1.74 (p, *J* = 6.6 Hz, 2H, C*H₂* alkane), 1.56 – 1.36 (m, 4H, C*H₂* alkane). ^{13}C NMR (75 MHz, $CDCl_3$) δ 156.54, 155.61, 151.65 (C=O carbamate, C=O Cbz, C_{Ar}-O), 136.7, 134.6, 128.7, 128.3, 127.8, 127.4, 126.1, 122.7-118,9 (d_a[1], $^{1}J_{C-F}$ = 283.0 Hz)120.5, 117.4 (*C*F_3), 111.8 (C-Ar), 68.40 – 67.09 (m, *C*H-(CF_3)$_2$) 68.1 (-O*C*H_2), 66.8 (-O*C*H_2), 62.2 (N-*C*H_2 spirocycle), 61.5 (N-*C*H_2 spirocycle), 43.7 (-C-*C*H_2-CH spirocycle), 41.1 (*C*H_2-NHCbz), 35.6 (-C-*C*H_2-CH spirocycle), 29.9, 29.0, 27.1, 23.5 (3 x -CH_2-*C*H_2-CH_2- linker, -*C*- spirocycle). ^{19}F NMR (282 MHz, $CDCl_3$) δ 3.88 (CF_3). LC-HRMS (ESI) calc. for $C_{30}H_{33}F_6N_2O_5$ $[M+H]^+$: 615.2288; found: 615.2259.

[1] For the quartet with intensities 1:3:3:1, signals of lower intensity are not noticeable in the ^{13}C NMR.

(*R*)-1,1,1-Trifluoro-3-((4-methoxybenzyl)oxy)propan-2-yl 6-(2-((5-(((benzyloxy)carbonyl)amino)pentyl)oxy)benzylidene)-2-azaspiro[3.3]heptane-2-carboxylate (19f)

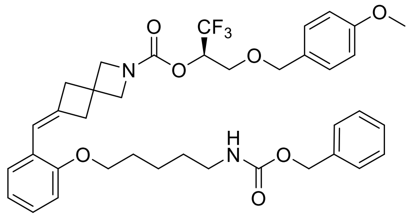

Building block **19b** (46 mg, 88 µmol) was subjected to the standard *N*-Boc deprotection procedure as described above. To a solution of **BB13** (29 mg, 132 µmol) in anhydrous DCM (5 mL), triethylamine (3 equiv.) and bis-(pentafluorphenyl)-carbonat (52 mg, 132 µmol) were added. The reaction mixture was stirred for 18 h at ambient temperature before a solution of the deprotected amine in DCM (5 mL) with TEA (2 equiv.) was added. The reaction mixture was stirred for a further 18 h at ambient temperature, whereupon it was concentrated in vacuo and purified via silica gel chromatography with a gradient elution of 0% to 50% EtOAc in cyclohexane. The title compound was obtained as a pale-yellow oil (21 mg, 34%). ^{1}H NMR (300 MHz, $CDCl_3$) δ 7.39 – 7.26 (m, 6H), 7.25 – 7.11 (m, 3H), 6.95 – 6.79 (m, 4H), 6.51 (t, *J* = 2.4 Hz, 1H), 5.40 (qd, *J* = 7.1, 4.0 Hz, 1H), 5.10 (s, 2H), 4.78 (t, *J* = 5.3 Hz, 1H), 4.50 (q, *J* = 11.7 Hz, 2H), 4.08 (s, 4H), 3.95 (t, *J* = 6.4 Hz, 2H), 3.80 (s, 3H), 3.70 (qd, *J* = 11.2, 5.5 Hz, 2H), 3.24 (q, *J* = 6.5 Hz, 2H), 3.19 (s, 2H), 3.06 (s, 2H), 1.83 (p, *J* = 6.6 Hz, 2H), 1.67 – 1.44 (m, 3H), 0.94 – 0.80 (m, 1H). ^{13}C NMR (75 MHz, $CDCl_3$) δ 159.51, 156.57, 155.57, 154.03, 136.66, 135.23, 129.51, 129.44, 128.66, 128.27, 127.72, 127.34, 126.24, 120.46, 117.10, 113.98, 111.82, 77.36, 73.03, 69.57 (q, *J* = 31.5 Hz), 68.05, 66.81, 66.19, 55.40, 43.71, 43.60, 41.08, 35.38, 29.88, 29.04, 23.47.

(*R*)-1,1,1-Trifluoro-3-hydroxypropan-2-yl 6-(2-((5-aminopentyl)oxy)benzyl)-2-azaspiro[3.3]heptane-2-carboxylate (20f)

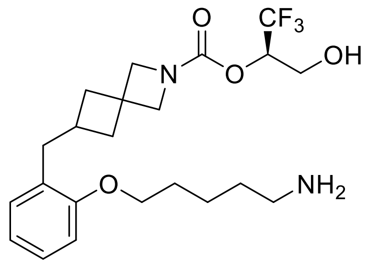

The hydrogenolysis was conducted as described above, using methanol as the reaction solvent. After RP-HPLC purification, the compound was obtained as the respective TFA salt as a colorless amorphous powder (14.5 mg, 86%). ^{1}H NMR (300 MHz, MeOD) δ 7.13 (td, *J* = 7.9, 1.8 Hz, 1H), 7.06 (dd, *J* = 7.5, 1.8 Hz, 1H), 6.92 – 6.77 (m, 2H), 5.20 (pd, *J* = 7.1, 3.8 Hz, 1H), 4.14 – 3.89 (m, 6H), 3.84 (dd, *J* = 12.4, 3.8 Hz, 1H), 3.72 (dd, *J* = 12.6, 7.3 Hz, 1H), 2.96 (t, *J* = 7.6 Hz, 2H), 2.68 (d, *J* = 7.4 Hz, 2H), 2.46 (p, *J* = 7.8 Hz, 1H), 2.31 – 2.17 (m, 2H), 1.99 – 1.81 (m, 4H), 1.81 – 1.69 (m, 2H), 1.67 – 1.55 (m, 2H). ^{13}C NMR (75 MHz, MeOD) δ 158.08, 155.85, 131.13, 129.82, 128.29, 121.36, 112.23, 72.78 (q, *J* = 30.6 Hz), 68.32, 59.72, 40.68, 39.42, 36.06, 30.99, 30.02, 28.33, 24.30.

2-(6-(2-(2-Aminoethoxy)benzyl)-2-azaspiro[3.3]heptane-2-carbonyl)-7-oxa-2,5-diazaspiro[3.4]octan-6-one (20a)

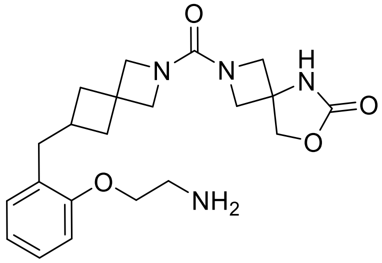

A mixture of 20% *t*BuOH in EtOAc (HPLC grade) was purged with N_2 for at least 15 min and kept under an N_2 atmosphere on a closed vial. In a pear-shaped flask, the starting material **19a** was dissolved in the previously prepared solvent mixture and purged with N_2 for 10 min. Next, Palladium on carbon (10 mol%) was added to the mixture and further purged with N_2 for an additional 15 min. Using an H_2-filled balloon, the reaction was then carried with constant bubbling of H_2 through the solution until completion (LC-MS monitoring). Solvent mixture levels were kept at 1-2 mL by adding small amounts of the previously prepared N_2-purged solvent mixture when needed. Palladium on carbon was removed from the reaction mixture by filtration with a PTFE syringe filter (pore size: 0.45 µm) using ACN and water to wash the filter. The crude was lyophilized, and the obtained intermediate **20a** was used in the next step without further purification.

3-(5,5-Difluoro-7,9-dimethyl-5H-5λ^4,6λ^4-dipyrrolo[1,2-c:2',1'-f][1,3,2]diazaborinin-3-yl)-N-(2-(2-((2-(6-oxo-7-oxa-2,5-diazaspiro[3.4]octane-2-carbonyl)-2-azaspiro[3.3]heptan-6-yl)methyl)phenoxy)ethyl)propanamide (9)

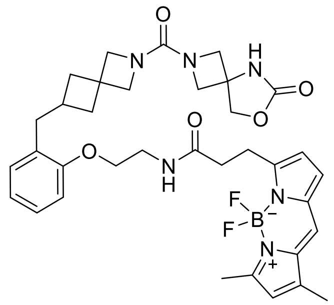

The crude **20a** (6.2 mg, 16 µmol, 1.2 equiv) was dissolved in ACN (1.3 mL) at 0 °C, then BODIPY-FL NHS ester (CAS RN 46616-66-2*,* 5.00 mg, 13 µmol) followed by DIPEA (9.0 µL, 7.0 µmol) were added. The reaction was stirred at room temperature in the dark for approximately 4 h (LC-MS monitoring of the reaction). Then, it is diluted with H_2O/ACN and freeze-dried. The crude was purified by RP-HPLC H_2O/ACN with 0.1% TFA 25 to 75% in 30 min to afford the target product (5.6 mg, 8.0 µmol, 64%). ^{1}H NMR (600 MHz, CD_3CN) δ 7.30 (s, 1H), 7.14 (td, *J* = 7.8, 1.8 Hz, 1H), 7.07 (dd, *J* = 7.6, 1.8 Hz, 1H), 6.92 – 6.83 (m, 3H), 6.67 (s, 1H, N*H*(C=O)), 6.48 (s, 1H), 6.30 (d, *J* = 4.1 Hz, 1H), 6.22 (s, 1H), 4.42 (s, 2H, OCH_2), 3.99 – 3.88 (m, 6H, 2 x N-CH_2 cyclic, OCH_2), 3.79 (s, 2H, N-CH_2 cyclic), 3.74 (s, 2H, N-CH_2 cyclic), 3.54 (q, *J* = 5.5 Hz, 2H, -NC$H_2$$CH_2$O-), 3.17 (t, *J* = 7.6 Hz, 2H, C_{sp2}-CH_2), 2.63 (d, *J* = 7.6 Hz, 2H, CH_2), 2.57 (t, *J* = 7.6 Hz, 2H, CH_2), 2.50 (s, 3H, CH_3 BODIPY), 2.38 (p, *J* = 7.8 Hz, 1H, 1H, C*H* spirocycle), 2.25 (s, 3H, CH_3 BODIPY), 2.14 – 2.12 (m, 2H, C-CH_2-CH spirocycle), 1.87 – 1.81 (m, 2H, C-CH_2-CH spirocycle).^{13}C NMR (151 MHz, CD_3CN) δ 172.5 (C=O amide), 163.2, 161.3, 158.6, 158.4, 157.5 (C_{Ar}-O, C=O carbamate, C=O urea, 2 x C_{Ar}-N_{Ar}+), 145.8, 136.0, 134.2, 131.0, 130.1, 129.4, 128.1, 125.7, 121.4, 112.3 (C-Ar), 75.1 (O*C*H_2), 67.4, 63.9, 63.38, 62.8 (4 x N-CH_2 cyclic, OCH_2), 55.1 (-*C*- carbamate spirocycle), 39.7,(-N*C*$H_2$$CH_2$O-), 39.3 (2 x C-*C*$H_2$-CH spirocycle), 36.9 (*C*H_2), 35.7 (-*C*- spirocycle), 35.4 (*C*H_2), 30.6 (C*H* spirocycle), 25.2 (C_{sp2}-*C*H_2), 15.1 (*C*H_3 BODIPY), 11.4 (*C*H_3 BODIPY). LC-HRMS calc. for $C_{35}H_{42}BF_2N_6O_5$ $[M+H]^+$: 675.3278; found: 675.3279.

2-(6-(2-(2-((7-Nitro-1l2,3l2-benzo[c][1,2,5]oxadiazol-4-yl)amino)ethoxy)benzyl)-2-azaspiro[3.3]heptane-2-carbonyl)-7-oxa-2,5-diazaspiro[3.4]octan-6-one (8)

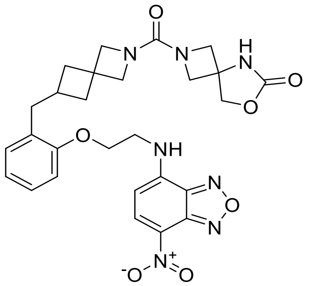

The crude **20a** starting material (12.0 mg, 0.03 mmol) was dissolved in MeOH (0.75 mL), and trimethylamine (13.0 µL, 0.09 mmol) was added. Next, a solution of NBD-fluoride (11.0 mg, 0.06 mmol) in MeOH (0.3 mL) was added dropwise. The reaction was stirred in the dark for 3.5 h. Next, the solvent was removed *in vacuo* at 20 °C and the reaction was purified through RP-HPLC 15-85% ACN in H_2O with 0.1% TFA to afford the title product (3.19 mg, 6.0 µmol, 19%).^{1}H NMR (600 MHz, CD_3CN) δ 8.53 (d, *J* = 8.8 Hz, 1H), 7.16 (td, *J* = 7.8, 1.7 Hz, 1H), 7.06 (dd, *J* = 7.4, 1.7 Hz, 1H), 6.92 (dd, *J* = 8.3, 1.1 Hz, 1H), 6.87 (td, *J* = 7.4, 1.1 Hz, 1H), 6.51 – 6.39 (m, 2H), 4.47 (s,

2H), 4.27 (t, *J* = 5.0 Hz, 2H), 4.05 – 3.83 (m, 6H), 3.67 (s, 2H), 3.60 (s, 2H), 2.55 (d, *J* = 7.5 Hz, 2H), 2.26 (p, *J* = 7.8 Hz, 1H), 2.03 – 1.96 (m, 2H), 1.75 – 1.68 (m, 2H). ^{13}C NMR (151 MHz, CD_3CN) δ 163.20, 158.45, 157.31, 145.82, 138.35, 131.23, 130.01, 128.26, 121.82, 112.38, 75.19, 66.82, 63.86, 63.44, 62.61, 55.22, 39.32, 37.11, 35.54, 30.57. LC-HRMS (ESI) calc. for $C_{27}H_{30}N_7O_7$ $[M+H]^+$: 564.2201; found: 564.2192.

3,7-Bis(dimethylamino)-5,5-dimethyl-3'-oxo-*N*-(5-(2-((2-(6-oxo-7-oxa-2,5-diazaspiro[3.4]octane-2-carbonyl)-2-azaspiro[3.3]heptan-6-yl)methyl)phenoxy)pentyl)-3'*H*,5*H*-spiro[dibenzo[b,e]siline-10,1'-isobenzofuran]-6'-carboxamide (10)

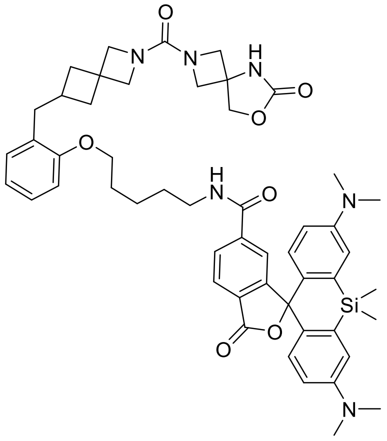

In analogy to the procedure described for **20a**, **19b** was treated with hydrogen and palladium on carbon to afford intermediate **20b**, which used in next step without further purification. LC-MS $[M+H]^+$: 442.9.The crude **20b** (8.96 mg, 0.02 mmol) was dissolved in ACN with 10% DMF (0.90 mL) at 0 °C, then the 2,5-dioxopyrrolidin-1-yl 3,7-bis(dimethylamino)-5,5-dimethyl-3'-oxo-3'*H*,5*H*-spiro[dibenzo[*b*,*e*]siline-10,1'-isobenzofuran]-6'-carboxylate (CAS RN 1426090-09-6, 5.00 mg, 9.0 µmol) followed by DIPEA (9.0 µL, 7.0 µmol) were added. The reaction was stirred at ambient temperature in the dark for approximately 4 h (LC-MS monitoring of the reaction). Then, it was diluted with H_2O/ACN and freeze-dried. The crude was purified by RP-HPLC H_2O/ACN with 0.1% TFA 25 to 75% in 30 min to afford the target product (6.0 mg, 7.0 µmol, 76%). ^{1}H NMR (600 MHz, MeOD) δ 8.33 (d, *J* = 8.2 Hz, 1H), 8.14 (dd, *J* = 8.2, 1.8 Hz, 1H), 7.74 (d, *J* = 1.7 Hz, 1H), 7.36 (d, *J* = 2.9 Hz, 2H), 7.13 (td, *J* = 7.8, 1.7 Hz, 1H), 7.04 (dd, *J* = 7.4, 1.7 Hz, 1H), 7.02 – 6.99 (m, 2H), 6.88 (dd, *J* = 8.2, 1.1 Hz, 1H), 6.83 (td, *J* = 7.3, 1.0 Hz, 1H), 6.77 (dd, *J* = 9.6, 2.9 Hz, 2H), 4.53 (s, 2H, OC*H₂*), 4.07 (s, 4H, OC*H₂*), 4.00 (t, *J* = 6.1 Hz, 2H), 3.93 (s, 2H), 3.84 (s, 2H), 3.47 (t, *J* = 7.1 Hz, 2H), 3.32 (s, 12H, 4 x N-CH_3), 2.64 (d, *J* = 7.4 Hz, 2H), 2.44 (p, *J* = 7.7 Hz, 1H, C*H* spirocycle), 2.22 – 2.16 (m, 2H, CH-CH_2-C spirocycle), 1.88 (ddd, *J* = 10.5, 7.9, 2.8 Hz, 4H, CH-CH_2-C spirocycle, -CH_2- alkyl), 1.74 (p, *J* = 7.2 Hz, 2H, -CH_2- alkyl), 1.67 – 1.59 (m, 2H, -CH_2- alkyl), 0.68 (s, 3H, Si-CH_3), 0.62 (s, 3H, Si-CH_3). ^{13}C NMR (151 MHz, MeOD) δ 166.6, 162.3, 159.0, 156.8 (C=O urea, C=O carbamate, *C*OO$^-$, C_{Ar}-O), 168.05, 163.72, 160.43, 158.21, 131.12, 129.87, 128.84, 128.28, 121.53, 121.24, 115.21, 112.27, 75.64 (O*C*H$_2$), 68.62 (O*C*H$_2$), 64.40, 63.80, 63.22, 55.81 (-*C*- carbamate spirocycle), 41.18, 40.96, 39.60, 37.43, 36.09 (-*C*- spirocycle), 31.11 (*C*H spirocycle), 30.26, 30.15 (-*C*H$_2$- alkyl), 24.93 (-*C*H$_2$- alkyl), -0.80 (Si-CH_3), -1.68 (Si-CH_3). LC-HRMS (ESI) calc. for $C_{51}H_{60}N_6O_7Si$ $[M+H]^+$: 897.4366; found: 897.4357.

1,1,1,3,3,3-Hexafluoropropan-2-yl 6-(2-(2-(3-(5,5-difluoro-7,9-dimethyl-5*H*-5λ[4],6 λ[4]-dipyrrolo[1,2-c:2',1'-f][1,3,2]diazaborinin-3-yl)propanamido)ethoxy)benzyl)-2-azaspiro[3.3]heptane-2-carboxylate (11)

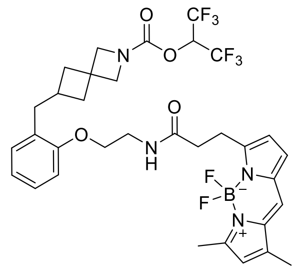

Intermediate **19c** (1.2 equiv.) was subjected to the general procedure for hydrogenolysis (see above) to afford free amine intermediate **20c**, which was used without further purification. BODIPY-FL carboxylic acid (CAS RN 165599-63-3, 4.4 mg, 0.015 mmol, 1.0 equiv.) was dissolved in THF (0.75 mL) and HATU (6.7 mg, 0.018 mmol, 1.2 equiv.), followed by 2,6-lutidine (3.2 mg, 0.03 mmol, 2.0 equiv.) were added. The mixture was stirred for 10 min, and then amine nucleophile **10** was added. The mixture was stirred overnight. The solvent was removed in vacuo at room temperature and purified via RP-HPLC 15-85% ACN in H_2O with 0.1% TFA to afford **11** (1.0 mg, 0.001 mmol, 9%). ^{1}H NMR (600 MHz, CD_3CN) δ 7.29 (s, 1H), 7.15 (td, *J* = 7.8, 1.8 Hz, 1H), 7.07 (dd, *J* = 7.5, 1.7 Hz, 1H), 6.92 – 6.82 (m, 3H), 6.63 (s, 1H, N-*H* amide), 6.29 (d, *J* = 4.0 Hz, 1H), 6.21 (s, 1H), 5.88 (hept, *J* = 6.4 Hz, 1H, OC*H*-$(CF_3)_2$), 4.05 – 3.89 (m, 6H, 2 x N-CH_2 spirocycle, OCH_2), 3.54 (m, 2H, CH_2NH(C=O)), 3.17 (m, 2H, C_{sp2}-CH_2), 2.63 (m, 2H, C_{sp2}-CH_2), 2.57 (t, *J* = 7.5 Hz, 2H, C_{sp2}-CH_2), 2.49 (s, 3H, Ar-CH_3), 2.41 – 2.38 (m, 1H, C*H* spirocycle), 2.25 (s, 3H, Ar-CH_3), 2.23 – 2.18 (m, 2H, C-CH_2-CH spirocycle), 1.92 – 1.87 (m, 2H, C-CH_2-CH spirocycle). ^{13}C NMR (151 MHz, CD_3CN) δ 172.51, 161.15, 158.66, 157.58, 152.33, 145.80, 136.01, 134.28, 130.99, 130.06, 129.50, 128.20, 125.78, 122.96, 121.46, 121.40, 121.38, 121.09, 117.78, 68.14 (dt, *J* = 68.6, 33.9 Hz), 67.53, 63.75, 63.07, 62.42, 61.82, 39.73, 39.15, 39.10, 37.06, 35.83, 35.45, 30.41, 25.25, 15.08, 11.42. ^{19}F NMR (564 MHz, CD_3CN) δ -74.50, -76.78 (2 x CF_3, 2 x BF). LC-HRMS (ESI) calc. for $C_{33}H_{36}BF_8N_4O_4$ $[M+H]^+$: 715.2696; found: 715.2701.

1,1,1,3,3,3-Hexafluoropropan-2-yl 6-(2-((5-(3,7-bis(dimethylamino)-5,5-dimethyl-3'-oxo-3'*H*,5*H*-spiro[dibenzo[*b*,*e*]siline-10,1'-isobenzofuran]-6'-carboxamido)pentyl)oxy)benzyl)-2-azaspiro[3.3]heptane-2-carboxylate (12)

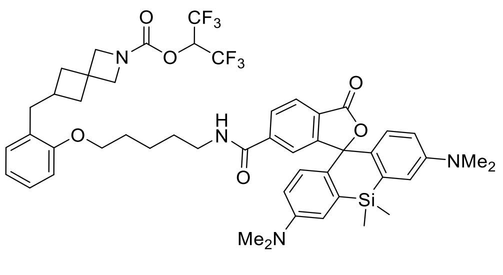

In analogy to the procedure described for **11**, **20d** was reacted with SiR-COOH (Spirochrome AG, Product N. SC004) to afford the target compound **12** (5.7 mg, 63%). ^{1}H NMR (600 MHz, CD_3CN) δ 8.13 (d, J = 8.1 Hz, 1H), 7.99 (dd, J = 8.1, 1.7 Hz, 1H), 7.63 (d, J = 1.6 Hz, 1H), 7.27 (t, J = 2.9 Hz, 2H), 7.25 (s, 1H), 7.12 (td, J = 7.8, 1.8 Hz, 1H), 7.03 (dd, J = 7.4, 1.7 Hz, 1H), 6.90 (dd, J =

9.4, 1.8 Hz, 2H), 6.85 (d, J = 8.2 Hz, 1H), 6.82 (td, J = 7.3, 1.1 Hz, 1H), 6.71 (dd, J = 9.3, 3.0 Hz, 2H), 5.87 (hept, J = 6.3 Hz, 1H), 3.95 (t, J = 46.8 Hz, 4H), 3.94 (t, J = 6.3 Hz, 2H), 3.36 (q, J = 6.6 Hz, 2H), 3.14 (s, 12H), 2.57 (d, J = 7.4 Hz, 2H), 2.37 (p, J = 7.4 Hz, 1H), 2.19 – 2.12 (m, 2H), 1.87 – 1.82 (m, 2H), 1.79 (p, J = 6.6 Hz, 2H), 1.63 (p, J = 7.1 Hz, 2H), 1.52 (qd, J = 9.0, 6.0 Hz, 2H), 0.64 (s, 3H), 0.56 (s, 3H). ^{13}C NMR (151 MHz, CD_3CN) δ 168.11, 166.44, 160.37, 160.13, 157.89, 152.59, 152.35, 140.38, 131.28, 130.98, 129.87, 128.73, 128.22, 127.22, 122.95, 121.13, 120.95, 116.07, 115.59, 112.39, 68.53, 68.15 (p, J = 34.0 Hz), 63.71, 63.01, 62.45, 61.80, 41.47, 40.60, 39.03, 37.06, 35.83, 30.57, 29.81, 29.71, 24.34, -0.39, -1.38. LC-HRMS (ESI) calc. for $C_{49}H_{55}F_6N_4O_6Si$ $[M+H]^+$: 937.3790; found: 937.3787.

1,1,1,3,3,3-Hexafluoropropan-2-yl 6-(2-(2-(hex-5-ynamido)ethoxy)benzyl)-2-azaspiro[3.3]heptane-2-carboxylate (13)

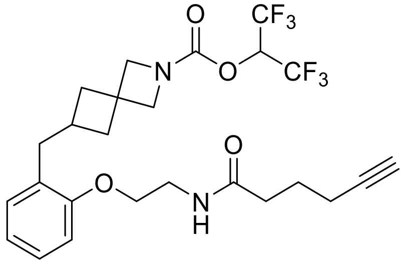

In analogy to the procedures described for **11**, **19c** (10 mg, 17.5 µmol) was subjected to hydrogenolysis and then reacted with 5-hexynoic acid (3.9 mg, 35 µmol) to afford the target compound (5 mg, 54%). ^{1}H NMR (300 MHz, $CDCl_3$) δ 7.18 (ddd, *J* = 8.1, 7.4, 1.8 Hz, 1H), 7.05 (dd, *J* = 7.4, 1.8 Hz, 1H), 6.90 (td, *J* = 7.4, 1.1 Hz, 1H), 6.82 (dd, *J* = 8.2, 1.1 Hz, 1H), 5.87 (s, 1H), 5.63 (hept, *J* = 6.2 Hz, 1H), 4.08 (d, *J* = 11.9 Hz, 2H), 4.04 (d, *J* = 5.3 Hz, 1H), 3.99 (d, *J* = 12.3 Hz, 2H), 3.69 (q, *J* = 5.4 Hz, 2H), 2.82 (s, 1H), 2.68 (d, *J* = 7.2 Hz, 2H), 2.51 (t, *J* = 7.4 Hz, 1H), 2.36 (t, *J* = 7.4 Hz, 1H), 2.28 (qd, *J* = 6.7, 2.7 Hz, 4H), 1.97 (dt, *J* = 4.5, 2.6 Hz, 2H), 1.88 (dtd, *J* = 11.8, 7.8, 4.3 Hz, 4H). ^{13}C NMR (75 MHz, $CDCl_3$) δ 177.17, 172.57, 156.25, 151.64, 130.17, 128.56, 127.59, 121.11, 111.46, 83.53, 83.25, 77.36, 69.45, 69.39, 68.28 – 66.99 (m), 66.83, 62.11 (q, *J* = 56.7 Hz), 39.29, 38.87, 36.51, 35.13, 32.43, 29.80, 29.73, 24.16, 23.48, 17.94, 17.89. LC-HRMS (ESI) calc. for $C_{25}H_{28}F_6N_2O_4$ $[M]^+$: 534.1953; found: 534.1987.

(*R*)-1,1,1-Trifluoro-3-hydroxypropan-2-yl 6-(2-((5-(((((*R,E*)-cyclooct-4-en-1-yl)oxy)carbonyl)amino)pentyl)oxy)benzyl)-2-azaspiro[3.3]heptane-2-carboxylate (14)

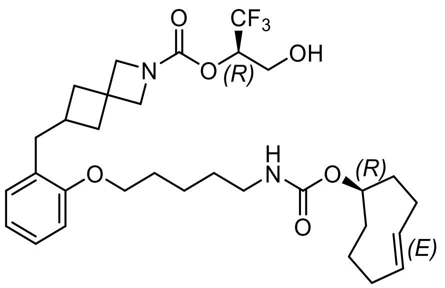

The TFA salt of **20f** (12.8 mg, 23 µmol) and (1R-4E-pS)-cyclooct-4-en-1-yl (4-nitrophenyl) carbonate (CAS 2389122-16-9, AmBeed, 8 mg, 27.6 µmol) were dissolved in anhydrous DMF

(0.5 mL) and TEA (9.2 µL, 3 equiv.) was added and stirred at ambient temperature for 3h. The reaction was quenched by the addition of a few drops of ethylamine in THF (2M) and continued stirring for 30 min. The mixture was separated between H_2O and EtOAc, and the organic phase was concentrated under reduced pressure and purified via RP-HPLC with gradient elution of 15%-99% ACN in H_2O (+0.1% TFA). The title compound was obtained as a colorless amorphous powder (6 mg, 44%). ^{1}H NMR (600 MHz, $CDCl_3$) δ 7.15 (td, *J* = 7.9, 1.7 Hz, 1H), 7.04 (dd, *J* = 7.4, 1.6 Hz, 1H), 6.86 (t, *J* = 7.4 Hz, 1H), 6.81 (d, *J* = 8.1 Hz, 1H), 5.77 – 5.45 (m, 2H), 5.18 (pd, *J* = 7.0, 3.4 Hz, 1H), 4.71 (s, 2H), 4.07 – 3.96 (m, 4H), 3.98 – 3.90 (m, 3H), 3.84 (dd, *J* = 12.5, 7.0 Hz, 1H), 3.18 (s, 2H), 2.67 (dd, *J* = 7.6, 4.2 Hz, 2H), 2.44 (p, *J* = 7.6 Hz, 1H), 2.35 (qt, *J* = 10.1, 5.0 Hz, 2H), 2.26 (qd, *J* = 8.4, 5.3 Hz, 2H), 2.20 – 2.07 (m, 1H), 2.06 – 1.68 (m, 10H), 1.67 – 1.45 (m, 6H). ^{13}C NMR (151 MHz, $CDCl_3$) δ 156.89, 154.48, 135.01, 133.18, 130.09, 129.92, 129.73, 128.84, 127.36, 123.29 (q, *J* = 282.4 Hz), 120.41, 111.22, 80.95, 72.35 (q, *J* = 30.6 Hz), 67.63, 60.33, 41.29, 41.03, 38.82, 38.78, 36.39, 35.12, 34.40, 32.62, 31.05, 30.03, 29.89, 29.20, 25.69, 24.87, 23.62. LC-HRMS (ESI) calc. for $C_{31}H_{43}F_3N_2O_6$ $[M]^+$: 596.3073; found: 596.3082.

3,3-Dimethyl-1-(6-oxo-6-((2-(2-((2-(6-oxo-7-oxa-2,5-diazaspiro[3.4]octane-2-carbonyl)-2-azaspiro[3.3]heptan-6-yl)methyl)phenoxy)ethyl)amino)hexyl)-2-((E)-3-((Z)-1,3,3-trimethylindolin-2-ylidene)prop-1-en-1-yl)-3H-indol-1-ium (S21)

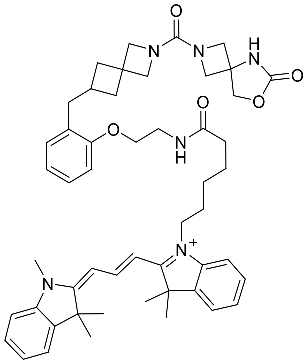

Fluorescent probe **S21** was synthesized by reacting **20a** and 1-(6-((2,5-dioxopyrrolidin-1-yl)oxy)-6-oxohexyl)-3,3-dimethyl-2-((*E*)-3-((*Z*)-1,3,3-trimethylindolin-2-ylidene)prop-1-en-1-yl)-3*H*-indol-1-ium tetrafluoroborate (CAS RN 2632339-91-2), in analogy to the procedure described for **9**. Yield: 12% (1.0 mg, 1.0 µmol). LC-HRMS (ESI) calc. for $C_{51}H_{63}N_6O_5$ M^+: 839.4854; found: 839.4846. ^{1}H NMR (300 MHz, CD_3CN) δ 8.44 (t, *J* = 13.5 Hz, 1H), 7.51 (dd, *J* = 7.4, 2.2 Hz, 2H), 7.43 (dtd, *J* = 9.2, 7.7, 1.2 Hz, 2H), 7.36 – 7.22 (m, 4H), 7.16 – 7.10 (m, 1H), 7.10 – 7.04 (m, 1H), 6.90 – 6.79 (m, 2H), 6.63 (s, 1H), 6.53 (s, 1H), 6.34 (d, *J* = 4.1 Hz, 1H), 6.29 (d, *J* = 4.0 Hz, 1H), 4.44 (s, 2H), 4.05 – 3.89 (m, 8H), 3.82 (s, 2H), 3.78 (s, 2H), 3.57 (s, 3H), 3.55 – 3.46 (m, 2H), 2.64 (d, *J* = 7.5 Hz, 2H), 2.23 – 2.11 (m, 5H), 1.89 – 1.73 (m, 4H), 1.73 – 1.63 (m, 3H), 1.71-1.69 (m, 12H), 1.46 (q, *J* = 7.8 Hz, 2H).

2.3. Further Supporting Information Compounds

Benzyl (2-(2-(((1-((4a*R*,8a*S*)-3-oxooctahydro-2*H*-pyrido[4,3-b][1,4]oxazine-6-carbonyl)azetidin-3-yl)oxy)methyl)phenoxy)ethyl)carbamate (S1)

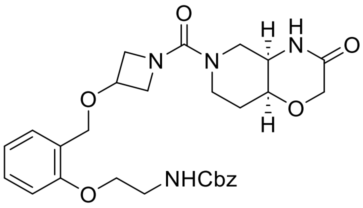

The synthetic procedure described for **3** was followed, starting from *N*-Boc protected derivative **BB1** (see below, building block synthesis), to afford target product **S1** (19.0 mg, 3.51 µmol, 40%). ^{1}H NMR (300 MHz, $CDCl_3$) δ 7.40 – 7.22 (m, 7H), 6.96 (td, *J* = 7.4, 1.0 Hz, 1H), 6.87 (d, *J* = 8.6 Hz, 1H), 6.35 (s, 1H, N*H*), 5.70 (s, 1H, N*H*), 5.11 (s, 2H, OC*H₂*), 4.45 (s, 2H, OC*H₂*), 4.36 – 3.86 (m, 10H), 3.78 (dd, *J* = 12.7, 4.8 Hz, 1H), 3.63 (q, *J* = 5.3 Hz, 2H), 3.43 (d, *J* = 13.6 Hz, 1H), 3.27 (td, *J* = 7.0, 3.6 Hz, 1H), 3.08 – 2.86 (m, 2H), 1.87 – 1.70 (m, 2H, C*H₂* HHPO). ^{13}C NMR (75 MHz, $CDCl_3$) δ 168.4, 162.3, 156.8 (C=O urea, C=O amide, C=O Cbz, C_{Ar}-O), 136.6, 130.4, 129.9, 128.7, 128.3, 128.2, 125.6, 121.2, 111.9, 69.5, 67.9, 67.8, 67.5, 66.9, 66.2, 58.5, 49.6, 46.3, 40.9, 39.7, 29.6 (*C*H₂ HHPO). LC-HRMS (ESI) calc. for $C_{28}H_{35}N_4O_7$ [M+H]$^+$: 538.2427; found: 539.2508

Benzyl (2-(2-(((2-((4a*R*,8a*S*)-3-oxooctahydro-2*H*-pyrido[4,3-b][1,4]oxazine-6-carbonyl)-2-azaspiro[3.3]heptan-6-yl)oxy)methyl)phenoxy)ethyl)carbamate (S3)

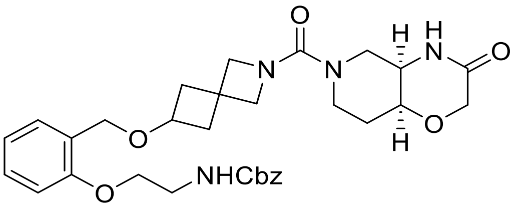

The synthetic procedure described for **3** was followed, starting from *N*-Boc protected derivative **BB3** (see below, building block synthesis), to afford target product **S3** (23.2 mg, 0.04 mmol, 57%). ^{1}H NMR (300 MHz, MeOD) δ 7.35 – 7.13 (m, 7H), 6.97 – 6.82 (m, 2H), 5.05 (s, 2H, OC*H₂*), 4.38 (s, 2H, OC*H₂*), 4.23 – 3.98 (m, 4H), 3.94 – 3.71 (m, 6H), 3.60 – 3.43 (m, 3H), 3.34 – 3.19 (m, 2H), 3.01 – 2.76 (m, 2H, C*H₂*-NHCbz), 2.46 – 2.28 (m, 2H), 2.15 – 1.94 (m, 2H), 1.91 – 1.65 (m, 2H). ^{13}C NMR (75 MHz, MeOD) δ 171.1, 163.5, 158.9, 158.0 (C=O urea, C=O amide, C=O Cbz, C_{Ar}-O), 138.3, 130.9, 130.3, 129.5, 129.0, 128.8, 127.8, 121.8, 112.8 (C-Ar), 70.5, 69.3, 68.2, 67.5, 66.2, 64.9, 64.0, 50.4, 47.1, 42.1, 41.6, 40.6, 31.8 (*C*-$(CH_2)_4$ spiroazetidine), 30.4 (*C*H₂ HHPO). LC-HRMS (ESI) calc. for $C_{31}H_{39}N_4O_7$ [M+H]$^+$: 579.2813; found: 579.2826.

Benzyl (2-(2-(((2-((4a*R*,8a*S*)-3-oxooctahydro-2*H*-pyrido[4,3-b][1,4]oxazine-6-carbonyl)-2-azaspiro[3.5]nonan-7-yl)oxy)methyl)phenoxy)ethyl)carbamate (S4)

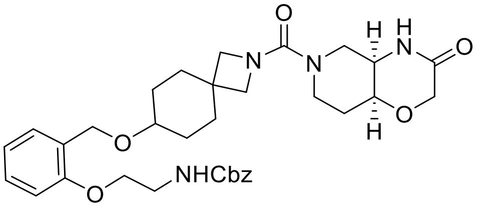

The synthetic procedure described for **3** was followed, starting from *N*-Boc protected derivative **BB4** (see below, building block synthesis), to afford target product **S4** (1.21 mg, 1.76 µmol, 8%). ^{1}H NMR (300 MHz, MeOD) δ 7.69 (d, *J* = 8.1 Hz, 1H), 7.37 – 7.17 (m, 7H, 6 x H_{Ar}, 1 x N*H*), 6.91 (t, *J* = 7.6 Hz, 2H), 5.08 (s, 2H, OC*H₂*), 4.54 (s, 2H, OC*H₂*), 4.26 – 4.10 (m, 2H), 4.05 (t, *J* = 5.4 Hz, 2H), 4.00 – 3.94 (m, 1H), 3.83 (dd, *J* = 13.0, 5.1 Hz, 1H), 3.74 – 3.58 (m, 4H), 3.52 (t, *J* = 5.4 Hz, 2H), 3.40 – 3.32 (m, 2H), 3.08 – 2.90 (m, 2H), 1.92 – 1.73 (m, 7H), 1.45 (q, *J* = 13.0 Hz, 4H). ^{13}C NMR (151 MHz, MeOD) δ 171.2, 163.9, 158.0 (C=O urea, C=O amide, C=O Cbz, C_{Ar}-O), 130.6, 130.0, 129.8, 129.5, 129.0, 128.8, 127.0, 121.8, 112.8, 70.5, 68.2, 68.1, 67.5, 66.1, 62.9, 62.5, 50.5, 47.2, 41.6, 40.6, 35.9, 34.1, 30.4, 29.2. LC-HRMS (ESI) calc. for $C_{33}H_{43}N_4O_7$ $[M+H]^+$: 607.3126; found: 607.3142.

Benzyl (2-(2-(((7-((4a*R*,8a*S*)-3-oxooctahydro-2*H*-pyrido[4,3-b][1,4]oxazine-6-carbonyl)-7-azaspiro[3.5]nonan-2-yl)oxy)methyl)phenoxy)ethyl)carbamate (S5)

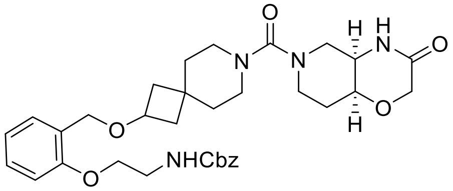

***N*-Boc deprotection.** *N*-Boc protected derivative **BB5** (52.5 mg, 0.10 mmol, see below building block synthesis) was dissolved in DCM (0.7 mL) at 0 °C and TFA (0.11 mL, 1.40 mmol) was added. The reaction mixture was stirred at that temperature until Boc deprotection was complete (30 min, LC-MS monitoring). The reaction was diluted with toluene (3.00 mL), and coevaporated with toluene (3.00 mL) under reduced pressure twice. The trifluoroacetate salt of the free amine was used in the next step without further purification.

Headgroup coupling with triphosgene. Triphosgene (CAS RN 32315-10-9, 53.8 mg, 0.10 mmol) and $NaHCO_3$ (33.6 mg, 0.08 mmol) were suspended in DCM (1.0 mL) at 0 °C. The deprotected amine derivative of **BB5** was dissolved in anhydrous DCM (1.5 mL) and added dropwise to the triphosgene mixture. The mixture was allowed to warm to r.t. and stirred overnight. The filtrate of this solution was then added dropwise at 0 °C to a solution of (4aR,8aS)-hexahydro-2H-pyrido[4,3-b][1,4]oxazin-3(4H)-one (CAS RN 2377107-31-6, 15.6 mg, 0.10 mmol) and DIPEA (70.0 µL, 0.40 mmol) in DCM (1.0 mL). The mixture was allowed to warm to r.t. and stirred for an additional 3 h. The mixture was concentrated in vacuo and purified by RP-HPLC (25 to 75% ACN in H2O with 0.1% TFA) to afford **S5** (23.6 mg, 39.0 µmol, 39%). ^{1}H NMR (300 MHz, MeOD) δ 7.37 – 7.19 (m, 7H), 6.91 (td, *J* = 8.3, 1.4 Hz, 2H), 5.08 (s, 2H, OC*H₂*), 4.42 (s, 2H, OC*H₂*), 4.24 – 4.00 (m, 5H), 3.96 (d, *J* = 3.0 Hz, 1H), 3.59 (dd, *J* = 12.7, 5.1 Hz, 1H), 3.52 (t, *J* = 5.4 Hz, 2H),

3.44 – 3.32 (m, 2H), 3.17 – 2.89 (m, 6H), 2.21 – 2.09 (m, 2H), 1.92 – 1.82 (m, 2H), 1.77 – 1.64 (m, 2H), 1.56 – 1.41 (m, 4H). ^{13}C NMR (75 MHz, $CDCl_3$) δ 171.2, 165.5, 158.9, 158.1 (C=O urea, C=O amide, C=O Cbz, C_{Ar}-O), 138.4, 130.9, 130.3, 129.5, 129.0, 128.7, 127.9, 121.8, 112.8, 70.6, 70.3, 68.2, 67.5, 66.2, 50.4, 45.2, 45.1, 42.9, 41.6, 40.6, 40.4, 37.7, 32.3, 30.3. LC-HRMS (ESI) calc. for $C_{33}H_{42}N_4O_7Na$ $[M+Na]^+$: 629.2946; found: 629.2961.

Benzyl (2-(2-((((1*R*,5*S*,6*R*)-3-((4a*R*,8a*S*)-3-oxooctahydro-2*H*-pyrido[4,3-b][1,4]oxazine-6-carbonyl)-3-azabicyclo[3.1.0]hexan-6-yl)methoxy)methyl)phenoxy)ethyl)carbamate (S6)

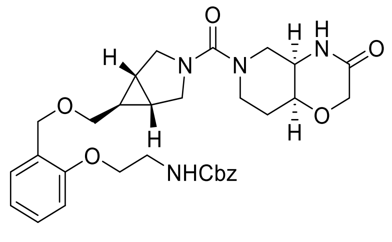

The synthetic procedure described for **S5** was followed, starting from *N*-Boc protected derivative **BB6** (see below, building block synthesis), to afford target product **S6** (4.14 mg, 7.0µmol, 12%). ^{1}H NMR (300 MHz, MeOD) δ 7.35 – 7.17 (m, 7H), 7.00 – 6.85 (m, 2H), 5.08 (s, 2H, OC*H2*), 4.51 (s, 2H, OC*H2*), 4.24 – 4.00 (m, 4H), 3.95 (d, *J* = 3.0 Hz, 1H), 3.64 (dd, *J* = 21.7, 10.7 Hz, 3H), 3.51 (t, *J* = 5.4 Hz, 2H), 3.44 – 3.33 (m, 4H), 3.28 – 3.21 (m, 2H), 3.02 – 2.85 (m, 2H), 1.83 (d, *J* = 11.1 Hz, 2H), 1.38 (s, 2H), 0.68 (dt, *J* = 7.0, 3.7 Hz, 1H). ^{13}C NMR (75 MHz, $CDCl_3$) δ 173.7, 167.1, 160.6 (C=O urea, C=O amide, C=O Cbz, C_{Ar}-O), 140.9, 133.4, 132.8, 132.1, 131.6, 131.3, 130.6, 124.3, 115.4, 75.0, 73.1, 71.1, 70.8, 70.0, 53.5, 53.1, 53.0, 44.8, 44.1, 32.9, 24.6, 24.5, 24.3. LC-HRMS (ESI) calc. for $C_{31}H_{38}N_4O_7Na$ $[M+Na]^+$: 601.2633; found: 601.2648.

Benzyl (3-(2-(((1-((4a*R*,8a*S*)-3-oxooctahydro-2*H*-pyrido[4,3-b][1,4]oxazine-6-carbonyl)azetidin-3-yl)methoxy)methyl)phenoxy)propyl)carbamate (S7)

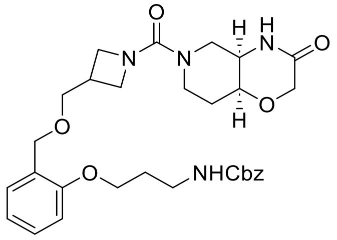

The synthetic procedure described for **3** was followed, starting from *N*-Boc protected derivative **BB7** (see below, building block synthesis), to afford target product **S7** (167 mg, 0.29 mmol, 87%).^{1}H NMR (300 MHz, $CDCl_3$) δ 7.37 – 7.27 (m, 7H), 7.07 (s, 1H, N*H*), 6.95 (td, *J* = 7.5, 1.0 Hz, 1H), 6.86 (d, *J* = 8.4 Hz, 1H), 5.59 (s, 1H, N*H*), 5.11 (s, 2H, OC*H2*), 4.56 (s, 2H, OC*H2*), 4.41 – 4.13 (m, 2H), 4.11 – 3.90 (m, 5H), 3.85 – 3.66 (m, 3H), 3.59 – 3.26 (m, 6H), 3.01 (t, *J* = 12.1 Hz, 2H), 2.85 – 2.68 (m, 1H), 2.11 – 1.63 (m, 4H). ^{13}C NMR (75 MHz, $CDCl_3$) δ 169.8, 162.0, 156.72, 156.63 (C=O urea, C=O amide, C=O Cbz, C_{Ar}-O), 136.6, 129.9, 129.4, 128.5, 128.3, 128.1, 126.0, 120.7, 111.2, 71.7, 69.3, 68.8, 67.3, 66.7, 66.4, 54.3, 54.0, 49.5, 46.0, 39.5, 39.1, 29.4, 29.1. LC-HRMS (ESI) calc. for $C_{30}H_{39}N_4O_7$ $[M+H]^+$: 567.2813.2633; found: 567.2855.

Benzyl (5-(2-(((1-((4a*R*,8a*S*)-3-oxooctahydro-2*H*-pyrido[4,3-b][1,4]oxazine-6-carbonyl)azetidin-3-yl)methoxy)methyl)phenoxy)pentyl)carbamate (S8)

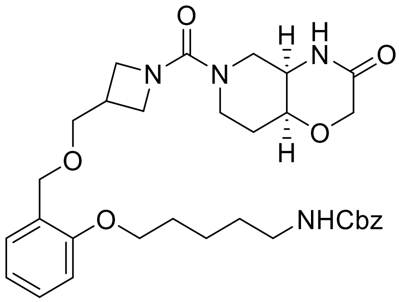

The synthetic procedure described for **3** was followed, starting from *N*-Boc protected derivative **BB8** (see below, building block synthesis), to afford target product **S8** (17 mg, 0.029 mmol, 29%). ^{1}H NMR (600 MHz, MeOD) δ 7.36 – 7.22 (m, 5H), 6.96 – 6.88 (m, 2H), 5.07 (s, 2H), 4.56 (s, 2H), 4.23 – 4.12 (m, 2H), 4.05 (dt, *J* = 16.5, 8.4 Hz, 2H), 4.00 (t, *J* = 6.2 Hz, 2H), 3.96 (q, *J* = 2.9 Hz, 1H), 3.84 – 3.81 (m, 1H), 3.78 (ddd, *J* = 18.6, 8.3, 5.5 Hz, 2H), 3.63 (d, *J* = 6.4 Hz, 2H), 3.57 (ddt, *J* = 13.5, 5.0, 2.1 Hz, 1H), 3.34 – 3.28 (m, 3H), 3.16 (t, *J* = 6.7 Hz, 2H), 3.01 – 2.95 (m, 2H), 2.81 (qd, *J* = 8.5, 5.7 Hz, 1H), 1.88 (dq, *J* = 14.5, 2.9 Hz, 1H), 1.85 – 1.76 (m, 3H), 1.56 (dq, *J* = 24.0, 7.9 Hz, 4H). ^{13}C NMR (151 MHz, MeOD) δ 171.14, 163.73, 158.23, 130.39, 130.09, 129.46, 128.94, 128.74, 127.71, 121.30, 112.49, 73.11, 70.51, 69.21, 68.97, 68.22, 67.31, 55.20, 55.17, 50.45, 47.14, 41.72, 40.59, 30.65, 30.46, 30.44, 30.10, 24.52. LC-HRMS (ESI) calc. for $C_{32}H_{42}N_4O_7Na$ $[M+Na]^+$: 617.2946; found: 617.2985.

Benzyl (2-(3-(((1-((4a*R*,8a*S*)-3-oxooctahydro-2*H*-pyrido[4,3-b][1,4]oxazine-6-carbonyl)azetidin-3-yl)oxy)methyl)phenoxy)ethyl)carbamate (S9)

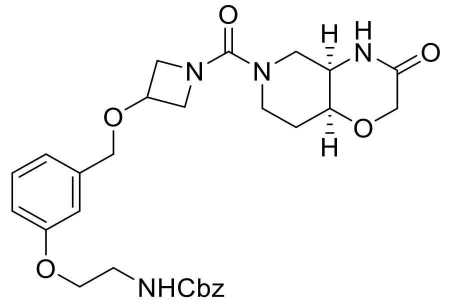

The synthetic procedure described for **3** was followed, starting from *N*-Boc protected derivative **BB9** (see below, building block synthesis), to afford target product **S9** (3.30 mg, 0.006 mmol, 10%). ^{1}H NMR (750 MHz, MeOD) δ 7.41 – 7.24 (m, 6H), 6.99 – 6.92 (m, 2H), 6.89 (dd, *J* = 8.2, 2.6 Hz, 1H), 5.11 (s, 2H, , O-C*H$_2$*-), 4.48 (s, 2H, , O-C*H$_2$*-), 4.38 (td, *J* = 6.7, 3.3 Hz, 1H), 4.26 – 4.14 (m, 5H), 4.06 (t, *J* = 5.6 Hz, 2H), 4.00 (q, *J* = 3.1 Hz, 1H), 3.93 – 3.87 (m, 2H), 3.84 (dd, *J* = 13.0, 4.7 Hz, 1H), 3.63 – 3.58 (m, 1H), 3.52 (t_a, *J* = 5.6 Hz, 2H, C*H$_2$*-NHCbz), 3.37 – 3.34 (m, 2H), 3.09 – 2.94 (m, 2H), 1.94 – 1.89 (m, 1H, –CH*H'*- HHPO), 1.87 – 1.80 (m, 1H, –C*H*H'- HHPO). ^{13}C NMR (189 MHz, MeOD) δ 171.2, 163.7, 160.4, 159.0 (C=O urea, C=O HHPO, C=O Cbz, C_{Ar}-O), 140.6, 138.4, 131.0, 130.6, 130.4, 129.5, 129.0, 128.8, 121.6, 115.2, 115.2 (C-Ar), 71.9, 70.5, 69.0, 68.2, 67.9, 67.5, 59.90, 59.86, 50.4, 47.2, 41.5, 40.7 (*C*H$_2$-NHCbz), 30.4 (-*C*H$_2$- HHPO). LC-HRMS (ESI) calc. for $C_{28}H_{35}N_4O_7$ [M+H]$^+$: 539.2500; found: 539.2502.

Benzyl (2-(3-(((2-((4a*R*,8a*S*)-3-oxooctahydro-2*H*-pyrido[4,3-b][1,4]oxazine-6-carbonyl)-2-azaspiro[3.3]heptan-6-yl)oxy)methyl)phenoxy)ethyl)carbamate (S11)

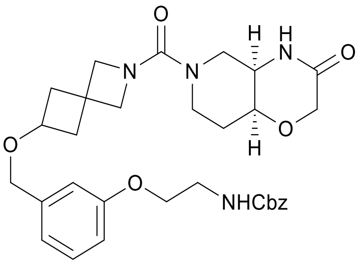

The synthetic procedure described for **3** was followed, starting from *N*-Boc protected derivative **BB11** (see below, building block synthesis), to afford target product **S11** (5.0 mg, 9.0 µmol, 27%). ^{1}H NMR (600 MHz, MeOD) δ 7.44 – 7.24 (m, 6H), 6.99 – 6.84 (m, 3H), 5.13 (s, 2H, OC*H$_2$*), 4.41 (s, 2H, OC*H$_2$*), 4.30 – 4.13 (m, 2H), 4.11 – 3.94 (m, 8H), 3.89 – 3.82 (m, 1H), 3.68 – 3.58 (m, 1H), 3.54 (t, *J* = 5.6 Hz, 2H), 3.40 – 3.36 (m, 1H), 3.07 – 2.95 (m, 2H, C*H$_2$*-NHCbz), 2.56 – 2.45 (m, 2H), 2.20 – 2.12 (m, 2H), 1.97 – 1.80 (m, 2H, C*H$_2$* HHPO). ^{13}C NMR (151 MHz, MeOD) δ 171.1, 163.6, 160.4, 159.0 (C=O urea, C=O amide, C=O Cbz, *C*$_{Ar}$-O), 141.2, 138.4, 130. 5, 129.5, 128.9, 128.7, 121.4, 115.2, 114.9 (C-Ar), 71.1, 70.5, 69.3, 68.2, 67.8, 67.5, 64.9, 64.0, 50.5, 47.1, 42.1, 41.5, 40.6 (*C*H$_2$-NHCbz), 32.0 (*C*-(CH$_2$)$_4$ spiroazetidine), 30.4 (*C*H$_2$ HHPO). LC-HRMS (ESI) calc. for C31H39N4O7 [M+H]$^+$: 579.2813; found: 579.2840.

Fluorescent probes **S12-S17** were synthesized following the hydrogenolysis followed by dye attachment procedures described above (for details for each probe, see Table S5).

Table S5. Method and yields for fluorescent probes **S12-S17**.

Compd.	Fluorophore reactant	Ligand reactant	Method	Yield	HRMS
**S12 (4)**	NBD-F	**3**	Analogous to **9**	72% (8.2 mg, 0.014 mmol)	calc for $C_{27}H_{32}N_7O_8$ $[M+H]^+$: 582.2307; found: 582.2331
**S13**	BODIPY-FL, NHS	**3**	Analogous to **10**	64% (2.3 mg, 3.0 µmol)	calc. for $C_{35}H_{43}BF_2N_6O_6Na$ $[M+Na]^+$: 715.3204; found: 715.3222.
**S14**	Cy3, NHS	**3**	Analogous to **11**	27% (2.0 mg, 2.0 µmol)	calc. for $C_{51}H_{65}N_6O_6$ $[M]^+$: 857.4960; found: 857.4997.
**S15**	5(6)-TAMRA, NHS	**3**	Analogous to **10**	15% (1.16 mg, 1.0 µmol)	calc. for $C_{46}H_{51}N_6O_9$ $[M]^+$: 831.3712; found: 831.3743.
**S16**	5(6)-TAMRA, NHS	**S7**	Analogous to **10**	43% (11.2 mg, 0.013 mmol)	calc. for $C_{47}H_{53}N_6O_{10}$ $[M+H]^+$: 861.3818; found: 861.3827.
**S17**	5(6)-TAMRA, NHS	**S8**	Analogous to **10**	23% (5.20 mg, 6.0 µmol)	calc for $C_{49}H_{58}N_6O_9$ $[M+2H]^{2+}$: 437.2127; found: 473.2149.

Benzyl (2-(2-(((1-(3-oxo-3,4-dihydro-2*H*-benzo[b][1,4]oxazine-6-carbonyl)azetidin-3-yl)methoxy)methyl)phenoxy)ethyl)carbamate (S18)

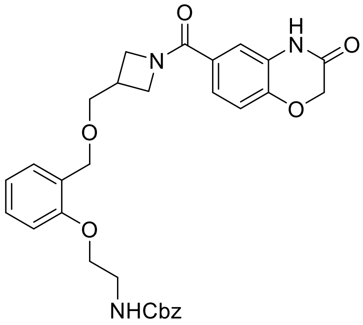

In analogy to the amide coupling procedure for **S19** (see next), compound **S24** was reacted with 3-oxo-3,4-dihydro-2H-benzo[b][1,4]oxazine-6-carboxylic acid (CAS RN: 134997-87-8) to afford the title compound (22.3 mg, 0.041 mmol, 50%). ^{1}H NMR (300 MHz, C*D*Cl$_3$) δ 8.64 (s, 1H), 7.41 – 7.24 (m, 8H), 7.16 (d, J = 8.3 Hz, 1H), 7.01 – 6.89 (m, 2H), 6.85 (d, J = 8.5 Hz, 1H), 5.54 (s, 1H), 5.11 (s, 2H), 4.64 (s, 2H), 4.53 (s, 2H), 4.16 (s, 2H), 4.09 (t, J = 5.1 Hz, 2H), 3.98 (s, 1H), 3.85 (s, 1H), 3.62 (dd, J = 10.2, 4.7 Hz, 4H), 2.84 (s, 1H).LC-HRMS (ESI) calc. for $C_{30}H_{32}N_3O_7$ [M+H]$^+$: 546.2235; found: 546.2239.

Benzyl (*R*)-(2-(2-(((1-(3-(2-oxooxazolidin-4-yl)propanoyl)azetidin-3-yl)methoxy)methyl)phenoxy)ethyl)carbamate (S19)

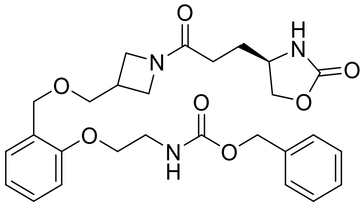

(*R*)-3-(2-Oxooxazolidin-4-yl)propanoic acid (12.7 mg, 0.08 mmol, **BB12**) was dissolved in THF/ACN 1:1 (1.60 mL), and then DIPEA (42.0 µL, 0.24 mmol) followed by HATU (36.5 mg, 96.0 µmol) were added. After stirring for 10 min, the deprotected amine derivative of **S24** (38.7 mg, 0.08 mmol, prepared as described for the Boc deprotection procedure) was added. The mixture was stirred at r.t. overnight, and then the mixture was directly diluted with ACN/H_2O and purified by RP-HPLC (15-85% ACN in H_2O with 0.1% TFA) to afford **S19** (16.3 mg, 0.032 mmol, 40%). ^{1}H NMR (600 MHz, CDCl$_3$) δ 7.37 (d, *J* = 4.2 Hz, 4H), 7.34 (p, *J* = 4.0 Hz, 1H), 7.32 – 7.28 (m, 2H), 6.99 (t, *J* = 7.4 Hz, 1H), 6.89 (d, *J* = 8.3 Hz, 1H), 6.38 (bs, 1H), 5.60 (bs, 1H), 5.14 (s, 2H), 4.58 (s, 2H), 4.47 (t, *J* = 8.6 Hz, 1H), 4.12 (t, *J* = 5.3 Hz, 2H), 4.01 (dd, *J* = 8.8, 5.9 Hz, 2H), 3.94 (p, *J* = 6.6 Hz, 1H), 3.80 (bs, 2H), 3.64 (d, *J* = 5.5 Hz, 2H), 3.60 (d, *J* = 6.4 Hz, 2H), 2.82 (s, 1H), 2.34 – 2.10 (m, 2H), 1.94 (s, 1H), 1.78 (dq, *J* = 13.4, 6.7 Hz, 1H). ^{13}C NMR (151 MHz, CDCl$_3$) δ 172.28, 160.15, 156.70, 136.53, 130.14, 129.62, 128.71, 128.39, 126.37, 121.19, 112.10, 71.47, 70.16, 68.97, 67.45, 67.09, 53.26, 52.03, 50.78, 40.82, 29.81, 28.43, 26.48. LC-HRMS (ESI) calc. for $C_{27}H_{34}N_3O_7$ [M+H]$^+$: 512.2389; found: 512.2391.

2.4. Building Blocks

***tert*-Butyl 3-((2-(2-(((benzyloxy)carbonyl)amino)ethoxy)benzyl)oxy)azetidine-1-carboxylate (BB1)**

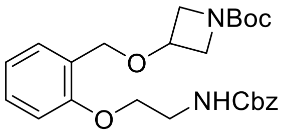

In analogy to the procedure described for **S24**, *tert*-butyl 3-hydroxy-azetidine-1-carboxylate (CAS RN 141699-55-0, 125 mg, 0.72 mmol) was reacted with benzyl (2-(2-(bromomethyl)phenoxy)ethyl)carbamate (**S23**, 131 mg, 0.36 mmol) to afford **BB1** (101 mg, 0.22 mmol, 61%). ^{1}H NMR (300 MHz, $CDCl_3$) δ 7.41 – 7.27 (m, 7H), 6.97 (t, *J* = 7.4 Hz, 1H), 6.86 (d, *J* = 8.1 Hz, 1H), 5.43 (s, 1H, N*H*-Cbz), 5.11 (s, 2H, OC*H₂*-Ar), 4.46 (s, 2H, OC*H₂* Cbz), 4.31 (dq, *J* = 10.8, 5.1 Hz, 1H, C*H* azetidine), 4.10 (t, *J* = 5.0 Hz, 2H), 4.07 – 3.97 (m, 2H), 3.92 – 3.81 (m, 2H), 3.62 (q, *J* = 5.4 Hz, 2H, OCH_2CH_2-NHCbz), 1.42 (d, *J* = 1.0 Hz, 9H, CH_3 Boc). ^{13}C NMR (75 MHz, $CDCl_3$) δ 156.6, 156.5 (C=O Cbz, C=O urea), 136.6, 130.1, 129.7, 128.7, 128.4, 128.3, 121.3, 112.0 (C-Ar), 79.7 (*C*H Boc), 67.6, 67.0, 66.5 (3 x OCH_2), 56.65, 40.8 (*C*H_2-NHCbz), 28.5 (*C*H_3 Boc). LC-HRMS (ESI) calc. for $C_{25}H_{33}N_2O_6$ $[M+H]^+$: 457.2333; found: 457.2320.

***tert*-Butyl 6-((2-(2-(((benzyloxy)carbonyl)amino)ethoxy)benzyl)oxy)-2-azaspiro[3.3]heptane-2-carboxylate (BB3)**

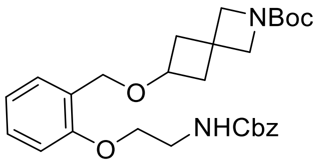

In analogy to the procedure described for **S24**, benzyl (2-(2-(bromomethyl)phenoxy)ethyl)carbamate **S23** was reacted with *tert*-butyl 6-hydroxy-2-azaspiro[3.3]heptane-2-carboxylate (CAS RN 1147557-97-8), to afford **BB3** (48.5 mg, 0.098 mmol, 65%). ^{1}H NMR (300 MHz, $CDCl_3$) δ 7.39 – 7.21 (m, 7H), 6.95 (td, *J* = 7.5, 1.0 Hz, 1H), 6.84 (d, *J* = 8.5 Hz, 1H), 5.54 (s, 1H, N*H*Cbz), 5.10 (s, 2H, OC*H₂*), 4.40 (s, 2H, OC*H₂*), 4.10 (t, *J* = 5.0 Hz, 2H), 3.93 – 3.72 (m, 5H, C*H* spirocycle, 2 x NC*H₂* spirocycle), 3.61 (q, *J* = 5.3 Hz, 2H, C*H₂*NHCbz), 2.41 – 2.28 (m, 2H, C-C*H₂*-CH spirocycle), 2.14 – 1.95 (m, 2H, C-C*H₂*-CH spirocycle), 1.43 (s, 9H, 3 x C*H₃* Boc). ^{13}C NMR (75 MHz, $CDCl_3$) δ 156.7, 156.6, 156.2 (C=O Cbz, C=O Boc, C_{Ar}-O), 136.5, 130.1, 129.5, 128.7, 128.5, 128.4, 126.6, 121.2, 112.1, 79.4 (*C*$(CH_3)_3$ Boc), 68.1, 67.6, 67.0, 65.8, 61.8, 60.5, 41.2, 40.7 (*C*H_2NHCz, 2 x C-*C*H_2-CH spirocycle), 30.1 (-*C*- spirocycle), 28.5 (3 x *C*H_3 Boc). LC-HRMS (ESI) calc. for $C_{28}H_{36}N_2O_6Na$ $[M+Na]^+$: 519.2466; found: 519.2450.

tert-Butyl 7-((2-(2-(((benzyloxy)carbonyl)amino)ethoxy)benzyl)oxy)-2-azaspiro[3.5]nonane-2-carboxylate (BB4)

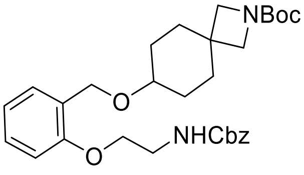

In analogy to the procedure described for **S24**, benzyl (2-(2-(bromomethyl)phenoxy)ethyl)carbamate **S23** was reacted with *tert*-butyl 7-hydroxy-2-azaspiro[3.5]nonane-2-carboxylate (CAS RN 1363383-18-9), to afford **BB4** (18.0 mg, 0.05 mmol, 26%). ^{1}H NMR (300 MHz, $CDCl_3$) δ 7.35 (s, 7H), 7.02 – 6.77 (m, 2H), 5.41 (s, 1H, N*H*Cbz), 5.10 (s, 2H, OC*H₂*), 4.53 (s, 2H, OC*H₂*), 4.08 (t, *J* = 5.0 Hz, 2H), 4.04 – 3.93 (m, 1H), 3.69 – 3.41 (m, 7H), 1.94 – 1.72 (m, 4H), 1.54 – 1.32 (m, 12H). ^{13}C NMR (75 MHz, $CDCl_3$) δ 156.6, 156.5, 156.2 (C=O Cbz, C=O Boc, C_{Ar}-O), 136.4, 129.5, 128.9, 128.6, 128.5, 128.2, 128.1, 127.9, 127.4, 121.1, 111.7, 79.2 ($C(CH_3)_3$ Boc), 67.3, 66.9, 65.2, 59.0, 40.7 (CH_2NHCbz), 34.5, 33.0, 28.5, 28.2 (3 x CH_3 Boc). LC-HRMS (ESI) calc. for $C_{30}H_{40}N_2O_6Na$ $[M+Na]^+$: 547.2779; found: 547.2774.

tert-Butyl 2-((2-(2-(((benzyloxy)carbonyl)amino)ethoxy)benzyl)oxy)-7-azaspiro[3.5]nonane-7-carboxylate (BB5)

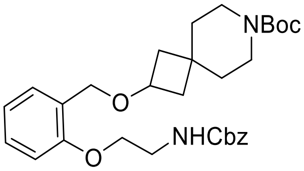

In analogy to the procedure described for **S24**, benzyl (2-(2-(bromomethyl)phenoxy)ethyl)carbamate **S23** was reacted with *tert*-butyl 6-hydroxy-2-azaspiro[3.3]heptane-2-carboxylate (CAS RN 240401-28-9), to afford **BB5** (59.6 mg, 0.114 mmol, 76%). ^{1}H NMR (300 MHz, $CDCl_3$) δ 7.38 – 7.26 (m, 7H), 6.95 (td, *J* = 7.5, 1.0 Hz, 1H), 6.85 (d, *J* = 8.2 Hz, 1H), 5.57 (s, 1H, N*H*Cbz), 5.10 (s, 2H, OC*H₂*), 4.41 (s, 2H, OC*H₂*), 4.16 – 3.95 (m, 3H), 3.61 (q, *J* = 5.3 Hz, 2H), 3.23 (dt, *J* = 20.9, 5.7 Hz, 4H), 2.12 (t, *J* = 10.0 Hz, 2H), 1.80 – 1.57 (m, 3H), 1.45 (m, 12H). ^{13}C NMR (75 MHz, $CDCl_3$) δ 156.7, 155.1 (C=O Cbz, C=O Boc, C_{Ar}-O), 136.6, 130.1, 129.4, 128.7, 128.4, 128.3, 126.9, 121.2, 112.2, 79.4 ($C(CH_3)_3$ Boc), 69.1, 67.7, 67.0, 65.7, 41.0, 40.9, 40.8, 39.6, 39.4, 36.7, 31.0, 28.6 (3 x CH_3 Boc), 27.1. LC-HRMS (ESI) calc. for $C_{30}H_{40}N_2O_6Na$ $[M+Na]^+$: 547.2779; found: 547.2799.

tert-Butyl (1_R_,5_S_)-6-(((2-(2-(((benzyloxy)carbonyl)amino)ethoxy)benzyl)oxy)methyl)-3-azabicyclo[3.1.0]hexane-3-carboxylate (BB6)

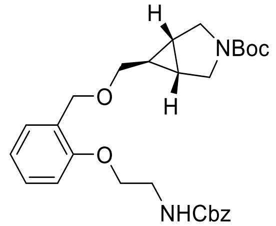

In analogy to the procedure described for **S24**, benzyl (2-(2-(bromomethyl)phenoxy)ethyl)carbamate **S23** was reacted with *tert*-butyl (1*R*,5*S*,6*R*)-6-(hydroxymethyl)-3-azabicyclo[3.1.0]hexane-3-carboxylate (CAS RN 827599-21-3) to afford **BB6** (32.3 mg, 0.069 mmol, 57%). ^{1}H NMR (300 MHz, $CDCl_3$) δ 7.40 – 7.20 (m, 7H), 6.96 (td, *J* = 7.5, 1.1 Hz, 1H), 6.85 (d, *J* = 8.3 Hz, 1H), 5.09 (s, 2H, -OC*H*$_2$-), 4.52 (s, 2H, -OC*H*$_2$-), 4.10 (t, *J* = 5.1 Hz, 2H), 3.65 – 3.39 (m, 5H), 3.36 – 3.19 (m, 3H), 1.49 – 1.32 (m, 11H, 3 x C*H*$_3$ Boc, 2 x C*H* fused cycle), 0.98 – 0.87 (m, 1H, C*H* propyl cycle). ^{13}C NMR (75 MHz, $CDCl_3$) δ 156.57, 156.53, 155.0 (C=O Cbz, C=O Boc, C_{Ar}-O), 136.6, 129.8, 129.2, 128.6, 128.30, 128.25, 127.0, 121.2, 112.2 (C-Ar), 79.4, 71.7, 68.0, 67.6, 66.9, 48.1 (*C*H cyclopropyl), 40.7 (*C*H$_2$-NHCbz), 28.6 (3 x *C*H$_3$ Boc), 27.0, 22.4 (*C*H fused cycle). LC-HRMS (ESI) calc. for $C_{28}H_{37}N_2O_6$ $[M+H]^+$: 497.2646; found: 497.2658.

tert-Butyl 3-(((2-(3-(((benzyloxy)carbonyl)amino)propoxy)benzyl)oxy)methyl)azetidine-1-carboxylate (BB7)

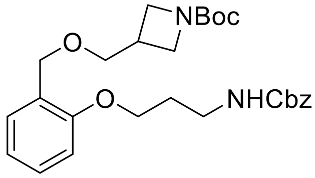

The title compound **BB7** was obtained following Scheme S1 in analogy to **S24.**

Ethyl 2-(3-(((benzyloxy)carbonyl)amino)propoxy)benzoate (S25)

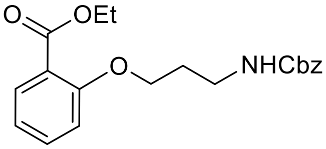

In analogy to the procedure described for **S22**, ethyl salicylate (CAS RN 118-61-6) was reacted with 3-(((benzyloxy)carbonyl)amino)propyl 4-methylbenzenesulfonate (CAS RN 68076-37-9) to afford the title compound **S25** (3.98 g, 11.2 mmol, 82%). ^{1}H NMR (300 MHz, $CDCl_3$) δ 7.85 (dd, *J* = 7.8, 1.8 Hz, 1H), 7.56 – 7.20 (m, 6H), 7.06 – 6.87 (m, 2H), 6.54 (s, 1H, N*H*Cbz), 5.11 (s, 2H, OC*H*$_2$), 4.24 (q, *J* = 7.1 Hz, 2H, OC*H*$_2$CH$_3$), 4.13 (t, *J* = 5.5 Hz, 2H, OC*H*$_2$), 3.57 – 3.40 (m, 2H, C*H*$_2$NHCbz), 2.06 (p, *J* = 5.6 Hz, 2H, -C*H*$_2$-), 1.29 (t, *J* = 7.1 Hz, 3H, OCH$_2$C*H*$_3$). ^{13}C NMR (75 MHz, $CDCl_3$) δ 166.0, 158.7, 157.0 (C=O CO_2Et, C=O Cbz, C_{Ar}-O), 137.1, 133.9, 132.0, 128.4, 128.0, 127.9, 120.4, 119.7, 112.7, 68.2 (O*C*H$_2$), 66.5 (O*C*H$_2$), 61.0 (O*C*H$_2$), 39.9 (*CH*$_2$NHCbz), 29.1 (-*C*H$_2$-), 14.4 (*C*H$_3$). LC-HRMS (ESI) calc. for $C_{20}H_{23}NO_5Na$ [M+Na]+: 380.1468; found: 380.1474.

Step i Benzyl (3-(2-(hydroxymethyl)phenoxy)propyl)carbamate (S26)

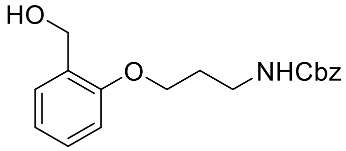

In analogy to the procedure described for **S22**, ethyl 2-(3-(((benzyloxy)carbonyl)amino)propoxy)benzoate was reacted to afford the title compound **S26** (2.35 g, 7.45 mmol, 98%). ^{1}H NMR (300 MHz, $CDCl_3$) δ 7.39 – 7.19 (m, 7H), 6.94 (td, *J* = 7.4, 1.1 Hz, 1H), 6.83 (d, *J* = 8.1 Hz, 1H), 5.08 (s, 2H, -OC*H₂*-), 4.66 (s, 2H, -OC*H₂*-), 4.03 (td, *J* = 5.9, 1.7 Hz, 2H, -OCH_2CH$_2$CH$_2$NHCbz), 3.40 (t, *J* = 6.3 Hz, 2H, -OCH$_2$CH$_2$CH_2NHCbz), 1.98 (p, *J* = 5.9 Hz, 2H, -OCH$_2$CH$_2$CH_2NHCbz). ^{13}C NMR (75 MHz, $CDCl_3$) δ 156.7, 156.6 (C=O Cbz, C_{Ar}-O), 136.6, 129.3, 129.0, 128.5, 128.09, 128.04, 120.7, 111.0 (C-Ar), 66.7 (O*C*H$_2$), 65.7 (O*C*H$_2$), 61.5 (O*C*H$_2$), 38.6 (*C*H$_2$-NHCbz), 29.4 (-*C*H$_2$- linker). LC-HRMS (ESI) calc. for $C_{18}H_{21}NO_4Na$ [M+Na]$^+$: 338.1363; found: 338.1353.

Step ii Benzyl (3-(2-(bromomethyl)phenoxy)propyl)carbamate (S27)

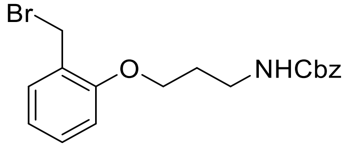

In analogy to the procedure described for **S23**, benzyl (3-(2-(hydroxymethyl)phenoxy)propyl)carbamate was reacted to afford the title compound **S27** (2.00 g, 5.29 mmol, 80%). ^{1}H NMR (300 MHz, $CDCl_3$) δ 7.45 – 7.18 (m, 7H), 6.98 – 6.79 (m, 2H), 5.11 (s, 2H, OCH_2), 4.54 (s, 2H, CH_2-Br), 4.10 (t, *J* = 5.8 Hz, 2H, , OCH_2 linker), 3.49 (q, *J* = 6.4 Hz, 2H, CH_2-NHCbz), 2.18 – 1.96 (m, 2H , -CH_2- linker). ^{13}C NMR (75 MHz, $CDCl_3$) δ 156.8, 156.7 (C=O Cbz, C_{Ar}-O), 136.7, 131.0, 130.8, 130.5, 130.4, 128.7, 128.2, 126.2, 125.9, 121.0, 111.77, 111.71 (C-Ar), 66.8 (-O*C*H$_2$-), 65.85 (-O*C*H$_2$-), 42.13 (-*C*H$_2$-NHCbz), 38.6 (*C*H$_2$-Br), 29.53 (-CH_2- linker). LC-HRMS (ESI) calc. for $C_{18}H_{21}BrNO_3$ [M+H]$^+$: 378.0699; found: 378.0700

Step iii *tert*-Butyl 3-(((2-(3-(((benzyloxy)carbonyl)amino)propoxy)benzyl)oxy)methyl)azetidine-1-carboxylate (BB7)

In analogy to the procedure described for **S24**, benzyl (3-(2-(bromomethyl)phenoxy)propyl)carbamate **S27** was reacted with *tert*-butyl 3-(hydroxymethyl)azetidine-1-carboxylate (CAS RN 142253-56-3) to afford the title compound **BB7** (401 mg, 0.83 mmol, 59%). ^{1}H NMR (300 MHz, $CDCl_3$) δ 7.41 – 7.28 (m, 7H), 6.95 (td, *J* = 7.5, 1.1 Hz, 1H), 6.85 (d, *J* = 8.2 Hz, 1H), 5.46 (s, 1H, N*H*Cbz), 5.10 (s, 2H, OCH_2), 4.54 (s, 2H, OCH_2), 4.05 (t, *J* = 5.8 Hz, 2H), 3.92 (t, *J* = 8.4 Hz, 2H), 3.67 – 3.51 (m, 4H), 3.43 (q, *J* = 6.1 Hz, 2H, CH_2NHCbz), 2.81 – 2.63 (m, 1H, C*H* azetidine), 2.02 (p, *J* = 6.0 Hz, 2H, CH_2 linker), 1.42 (s, 9H, 3 x CH_3 Boc). ^{13}C NMR (75 MHz, $CDCl_3$) δ 156.7, 156.6, 156.5 (C=O Cbz, C=O Boc, C_{Ar}-O), 136.8, 129.6, 129.2, 128.6, 128.2, 126.4, 120.8, 111.4, 79.4 (*C*(CH$_3$)$_3$ Boc), 72.4 (O*C*H$_2$), 68.7 (O*C*H$_2$), 66.7 (O*C*H$_2$), 66.4 (O*C*H$_2$), 52.0 (2 x N-*C*H$_2$- azetidine), 39.1 (*C*H$_2$NHCbz), 29.6(*C*H azetidine), 28.5 (3 x *C*H$_3$ Boc), 27.1 (-CH_2- linker). LC-HRMS (ESI) calc. for $C_{27}H_{37}N_2O_6$ [M+H]$^+$: 485.2646; found: 485.2646.

BB8 *tert*-Butyl 3-(((2-((5-(((benzyloxy)carbonyl)amino)pentyl)oxy)benzyl)oxy)methyl)azetidine-1-carboxylate

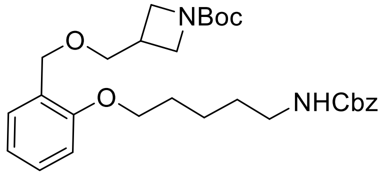

The title compound **BB8** was obtained following Scheme S1 in analogy to **S24.**

Ethyl 2-((5-(((benzyloxy)carbonyl)amino)pentyl)oxy)benzoate (S28)

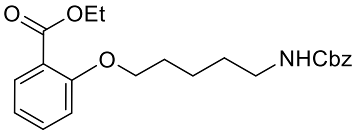

In analogy to the procedure described for **S22**, ethyl salicylate (CAS RN 118-61-6) was reacted with 5-(((benzyloxy)carbonyl)amino)pentyl 4-methylbenzenesulfonate (**15b**, CAS RN 93066-51-4) to afford the title compound **S28** (1.0 g, 2.59 mmol, 89%). ^{1}H NMR (300 MHz, $CDCl_3$) δ 7.77 (dd, *J* = 7.7, 1.8 Hz, 1H), 7.52 – 7.28 (m, 6H), 7.04 – 6.84 (m, 2H), 5.09 (s, 2H, O-CH_2), 4.31 (q, *J* = 7.1 Hz, 2H, O-CH_2-CH_3), 4.02 (t, *J* = 6.2 Hz, 2H, O-CH_2), 3.23 (m, 2H, -CH_2-NHCbz), 1.84 (p, *J* = 6.5 Hz, 2H, -CH_2- alkane), 1.64 – 1.53 (m, 4H, 2 x -CH_2- alkane), 1.33 (t, *J* = 7.1 Hz, 3H, O-CH_2-CH_3). ^{13}C NMR (75 MHz, $CDCl_3$) δ 166.6, 158.6, 156.6 (C=O CO_2Et, C=O Cbz, C_{Ar}-O), 136.8, 133.4, 131.66, 128.6, 128.22, 128.19, 120.8, 120.3, 113.2 (*C*-Ar), 68.5, 66.7, 60.9 (3 x O-CH_2), 41.0 (-CH_2-NHCbz), 31.1, 28.8, 23.2 (3 x CH_2 alkane), 14.44 (O-CH_2CH_3). LC-HRMS (ESI) calc. for $C_{22}H_{27}NO_5Na$ $[M+Na]^+$: 408.1781; found: 408.1798.

Step i Benzyl (5-(2-(hydroxymethyl)phenoxy)pentyl)carbamate (S29)

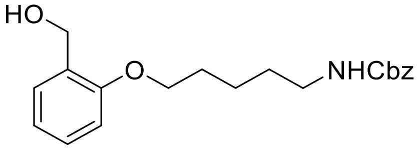

In analogy to the procedure described for **S22**, ethyl 2-((5-(((benzyloxy)carbonyl)amino)pentyl)oxy)benzoate was reacted to afford the title compound (737 mg, 2.15 mmol, 86%). ^{1}H NMR (300 MHz, $CDCl_3$) δ 7.41 – 7.19 (m, 7H), 6.93 (td, *J* = 7.4, 1.0 Hz, 1H), 5.10 (s, 2H, OCH_2), 4.68 (s, 2H, OCH_2), 4.01 (t, *J* = 6.3 Hz, 2H, OCH_2), 3.23 (t, *J* = 6.6 Hz, 2H CH_2NHCbz), 1.84 (p, *J* = 6.5 Hz, 2H, -CH_2- alkane), 1.68 – 1.46 (m, 4H, 2 x -CH_2- alkane). ^{13}C NMR (75 MHz, $CDCl_3$) δ 157.0, 156.6 (C=O Cbz, C_{Ar}-O), 136.7, 129.3, 129.1, 128.9, 128.7, 128.3, 128.3, 120.8, 111.2, 67.7 (OCH_2), 66.8 (OCH_2), 62.3 (CH_2OH), 41.0 (CH_2NHCbz), 29.9 (-CH_2- alkane), 27.1 (-CH_2- alkane), 23.5 (-CH_2- alkane). LC-HRMS (ESI) calc. for $C_{20}H_{25}NO_4Na$ $[M+Na]^+$: 366.1676; found: 366.1691.

Step ii Benzyl (5-(2-(bromomethyl)phenoxy)pentyl)carbamate (S30)

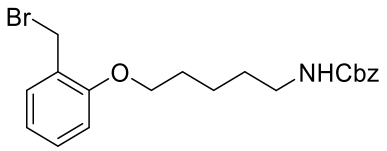

Benzyl (5-(2-(hydroxymethyl)phenoxy)pentyl)carbamate (714 mg, 2.08 mmol) was reacted following the synthetic procedure described for **S23** to afford the title compound **S30** (590 mg, 1.45 mmol, 70%). ^{1}H NMR (300 MHz, $CDCl_3$) δ 7.40 – 7.22 (m, 7H), 6.97 – 6.79 (m, 2H), 5.10 (s, 2H, O*CH₂*), 4.80 (d, *J* = 7.2 Hz, 1H, N*H*Cbz), 4.56 (s, 2H, C*H₂*-Br), 4.03 (t, *J* = 6.1 Hz, 2H, OC*H₂*), 3.30 – 3.17 (m, 2H, C*H₂*NHCbz), 1.90 – 1.80 (m, 2H, -C*H₂*- linker), 1.62 – 1.58 (m, 4H, 2 x -C*H₂*- linker). ^{13}C NMR (75 MHz, $CDCl_3$) δ 157.0, 156.6 (C=O Cbz, C_{Ar}-O), 136.8, 131.0, 130.7, 130.3, 128.7, 128.3, 128.2, 126.3, 120.7, 111.8, 111.7, 67.9 (O*C*H_2), 66.8 (O*C*H_2), 41.1 (*C*H_2NHCbz), 29.8, 29.4, 29.0 (2 x *C*H_2 linker, *C*H_2Br), 23.5 (-*C*H_2- linker). LC-HRMS (ESI) calc. for $C_{20}H_{24}BrNO_3Na$ $[M+Na]^+$: 430.0814; found: 430.0820.

Step iii *tert*-Butyl 3-(((2-((5-(((benzyloxy)carbonyl)amino)pentyl)oxy)benzyl)oxy)methyl)azetidine-1-carboxylate

In analogy to the procedure described for **S24**, benzyl (5-(2-(hydroxymethyl)phenoxy)pentyl)carbamate **S30** was reacted with *tert*-butyl 3-(hydroxymethyl)azetidine-1-carboxylate (CAS RN 142253-56-3) to afford **BB8** 67.6 mg, 0.132 mmol, 53%) was obtained. ^{1}H NMR (600 MHz, $CDCl_3$) δ 7.38 – 7.28 (m, 5H), 7.27 – 7.19 (m, 2H), 6.94 (t, J = 7.4 Hz, 1H), 6.84 (d, J = 8.3 Hz, 1H), 5.10 (s, 2H, OC*H₂*), 4.97 (s, 1H, N*H*Cbz), 4.56 (s, 2H, OC*H₂*), 4.00 – 3.93 (m, 4H), 3.70 – 3.64 (m, 2H), 3.64 – 3.59 (m, 2H), 3.23 (q, J = 6.7 Hz, 2H, C*H₂*NHCbz), 2.82 – 2.73 (m, 1H, C*H* azetidine), 1.85 – 1.77 (m, 2H, C*H₂* linker), 1.63 – 1.48 (m, 4H, C*H₂* linker), 1.43 (s, 9H, 3 x C*H₃* Boc). ^{13}C NMR (151 MHz, $CDCl_3$) δ 156.63, 156.58, 156.55 (C=O Boc, C=O Cbz, C_{Ar}-O), 136.8, 129.1, 128.9, 128.6, 128.21, 128.17, 126.7, 120.5, 111.2, 79.4 (*C*$(CH_3)_3$ Boc), 72.6, 68.2, 67.8, 66.7 (4 x O*C*H_2), 52.0 (2 x N*C*H_2 azetidine), 41.1 (*C*H_2NHCbz), 29.8, 29.0, 28.8 (C*H* azetidine, 2 x *C*H_2 linker), 28.5 (3 x CH_3 Boc), 23.5 (*C*H_2 linker). LC-HRMS (ESI) Calc for $C_{29}H_{40}N_2O_6Na$ $[M+Na]^+$: 535.2779; found: 537.2784.

***tert*-Butyl 3-((3-(2-(((benzyloxy)carbonyl)amino)ethoxy)benzyl)oxy)azetidine-1-carboxylate (BB9)**

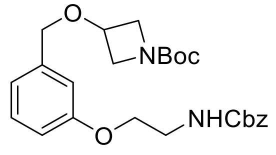

The title compound **BB9** was obtained following Scheme S1 in analogy to **S24.**

Ethyl 3-(2-(((benzyloxy)carbonyl)amino)ethoxy)benzoate (S31)

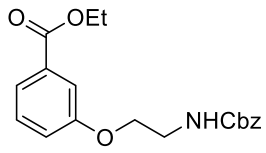

Starting from ethyl 3-hydroxy benzoate (1.25 g, 7.5 mmol) and benzyl (2-bromoethyl)carbamate (1.29 g, 5.0 mmol), and in analogy to the procedure described for **S22**, the title compound was obtained (1.05 g, 3.05 mmol, 61%). ^{1}H NMR (300 MHz, $CDCl_3$) δ 7.66 (dt, *J* = 7.7, 1.3 Hz, 1H), 7.54 (dd, *J* = 2.7, 1.5 Hz, 1H), 7.40 – 7.27 (m, 6H), 7.12 – 7.02 (m, 1H), 5.22 (s, 1H, N*H*-Cbz), 5.12 (s, 2H, $OC\textit{H}_2CH_3$), 4.37 (q, *J* = 7.1 Hz, 2H, $OC\textit{H}_2$), 4.09 (t, *J* = 5.1 Hz, 2H, $OC\textit{H}_2$), 3.63 (q, *J* = 5.4 Hz, 2H, $C\textit{H}_2$NHCbz), 1.39 (t, *J* = 7.1 Hz, 3H, $OCH_2C\textit{H}_3$). ^{13}C NMR (75 MHz, $CDCl_3$) δ 166.5, 158.5, 156.5 (C=O CO_2Et, C=O Cbz, $\textit{C}_{Ar}$-O), 136.5, 132.1, 129.6, 128.7, 128.4, 128.3, 122.6, 119.7, 114.9, 67.3 ($O\textit{C}H_2$), 67.1 ($O\textit{C}H_2$), 61.3 ($O\textit{C}H_2$), 40.7 ($C\textit{H}_2$NHCbz), 14.5 ($\textit{C}H_3$). LC-HRMS (ESI) calc. for $C_{19}H_{22}NO_5$ $[M+H]^+$: 366.1492; found: 366.1508.

Step i Benzyl (2-(3-(hydroxymethyl)phenoxy)ethyl)carbamate (S32)

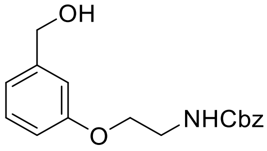

Starting from ethyl 3-(2-(((benzyloxy)carbonyl)amino)ethoxy)benzoate (199 mg, 0.58 mmol) and following the synthetic procedure described for **S22**, the title compound (115 mg, 0.38 mmol, 66%) was obtained. ^{1}H NMR (300 MHz, $CDCl_3$) δ 7.43 – 7.16 (m, 6H), 7.00 – 6.85 (m, 2H), 6.87 – 6.70 (m, 1H), 5.11 (s, 2H, O-$C\textit{H}_2$-), 4.66 (s, 2H, O-$C\textit{H}_2$-), 4.04 (t, *J* = 5.1 Hz, 2H, -$OC\textit{H}_2CH_2$NHCbz), 3.60 (d, *J* = 5.3 Hz, 2H, -$OCH_2C\textit{H}_2$NHCbz). ^{13}C NMR (75 MHz, $CDCl_3$) δ 158.8, 156.6 (C=O Cbz, $\textit{C}_{Ar}$-O), 142.8, 136.5, 129.8, 128.7, 128.32, 128.28, 119.7, 113.8, 112.9 (C-Ar), 67.01 (O-$\textit{C}H_2$-), 66.96 (O-$\textit{C}H_2$-), 65.2 (O-$\textit{C}H_2$-), 40.7 (-$\textit{C}H_2$-NHCbz). LC-HRMS (ESI) calc. for $C_{17}H_{20}NO_4$ $[M+H]^+$: 302.1387; found: 302.1373.

Step ii Benzyl (2-(3-(bromomethyl)phenoxy)ethyl)carbamate (S33)

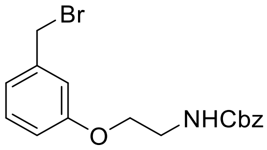

Starting from alcohol benzyl (2-(3-(hydroxymethyl)phenoxy)ethyl)carbamate (663 mg, 2.20 mmol) and following the synthetic procedure described for **S23**, the title compound (503 mg, 1.38 mmol, 63%) was obtained. ^{1}H NMR (300 MHz, $CDCl_3$) δ 7.42 – 7.22 (m, 6H), 7.03 – 6.97 (m, 1H), 6.95 – 6.90 (m, 1H), 6.88 – 6.79 (m, 1H), 5.24 (s, 1H, N*H*Cbz), 5.13 (s, 2H, $OC\textit{H}_2$), 4.46 (s, 2H, $C\textit{H}_2$Br), 4.06 (t, *J* = 5.1 Hz, 2H, $OC\textit{H}_2$), 3.63 (q, *J* = 5.4 Hz, 2H, $C\textit{H}_2$NHCbz).^{13}C NMR (75 MHz, $CDCl_3$) δ 158.8, 156.5 (C=O Cbz, $\textit{C}_{Ar}$-O), 139.4, 139.2, 136.5, 130.1, 128.7, 128.33, 128.29, 121.9, 121.4, 115.2, 114.7, 114.7, 67.0 (2 x $OC\textit{H}_2$), 40.7 ($\textit{C}H_2$NHCbz), 33.4 (CH_2Br). LC-HRMS (ESI) calc. for $C_{17}H_{19}BrNO_3$ $[M+H]^+$: 366.0524; found: 366.0551.

Step iii *tert*-Butyl 3-((3-(2-(((benzyloxy)carbonyl)amino)ethoxy)benzyl)oxy)azetidine-1-carboxylate (BB9)

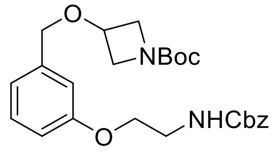

Starting from benzyl (2-(3-(bromomethyl)phenoxy)ethyl)carbamate **S33** (80.1 mg, 0.22 mmol) and *tert*-butyl 3-hydroxyazetidine-1-carboxylate (57.2 mg, 0.33 mmol), and following the synthetic procedure described for **S24**, target product **BB9** (55.1 mg, 0.12 mmol, 55%) was obtained. ^{1}H NMR (300 MHz, $CDCl_3$) δ 7.32 – 7.09 (m, 5H), 6.86 – 6.68 (m, 3H), 5.28 – 5.13 (m, 1H), 5.02 (s, 1H), 4.51 – 4.40 (m, 1H), 4.04 (ddd, *J* = 9.5, 6.7, 1.1 Hz, 2H), 3.96 (dd, *J* = 9.8, 6.0 Hz, 3H), 3.77 (dd, *J* = 9.3, 4.2 Hz, 2H), 3.70 (ddd, *J* = 9.4, 4.4, 1.1 Hz, 2H), 1.34 (d, *J* = 1.3 Hz, 12H). ^{13}C NMR (75 MHz, $CDCl_3$) δ 158.77, 156.52, 156.49, 138.97, 136.44, 129.80, 128.66, 128.30, 128.26, 120.65, 114.08, 113.98, 79.79, 79.71, 70.89, 67.39, 66.99, 66.95, 61.54, 59.08, 56.62, 40.67, 28.48. LC-HRMS (ESI) calc. for $C_{25}H_{33}N_2O_6$ $[M+H]^+$: 457.2333; found: 457.2328.

***tert*-Butyl 3-(((3-(2-(((benzyloxy)carbonyl)amino)ethoxy)benzyl)oxy)methyl)azetidine-1-carboxylate (BB10)**

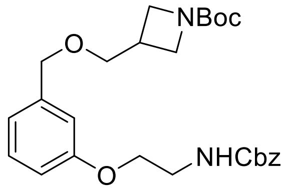

Benzyl (2-(3-(bromomethyl)phenoxy)ethyl)carbamate **S33** (120.2 mg, 0.33 mmol) was reacted with *tert*-butyl 3-(hydroxymethyl)azetidine-1-carboxylate (92.7 mg, 0.50 mmol), in analogy to the procedure described for **S24** to afford **BB10** (85.0 mg, 0.18 mmol, 55%). ^{1}H NMR (300 MHz, $CDCl_3$) δ 7.40 – 7.20 (m, 6H), 7.01 – 6.74 (m, 3H), 5.31 – 5.06 (m, 3H, N*H*Cbz, OC*H*$_2$), 4.70 – 4.42 (m, 3H), 4.07 – 3.91 (m, 4H), 3.74 – 3.51 (m, 5H), 2.88 – 2.69 (m, 1H, C*H* azetidine), 1.43 (d, *J* = 1.2 Hz, 9H, 3 x CH_3 Boc). LC-HRMS (ESI) calc. for $C_{26}H_{34}N_2O_6Na$ $[M+Na]^+$: 493.2309; found: 493.2312

***tert*-Butyl 6-((3-(2-(((benzyloxy)carbonyl)amino)ethoxy)benzyl)oxy)-2-azaspiro[3.3]heptane-2-carboxylate (BB11)**

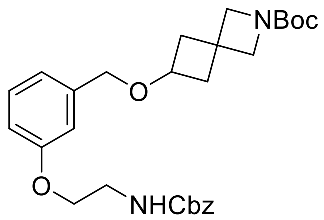

Benzyl (2-(3-(bromomethyl)phenoxy)ethyl)carbamate **S33** (54.6 mg, 0.15 mmol) was reacted with *tert*-butyl 6-hydroxy-2-azaspiro[3.3]heptane-2-carboxylate (48.0 mg, 0.23 mmol), in analogy to the procedure described for **S24** to afford **BB11** (21.0 mg, 0.042 mmol, 28%). ^{1}H NMR (300 MHz,

$CDCl_3$) δ 7.36 (d, *J* = 4.9 Hz, 5H), 7.22 (d, *J* = 7.8 Hz, 1H), 6.93 – 6.74 (m, 3H), 5.11 (s, 2H, OC*H₂*), 4.35 (s, 2H, OC*H₂*), 4.04 (t, *J* = 5.1 Hz, 2H, OC*H₂*), 4.01 – 3.78 (m, 5H, N-C*H₂*-C spirocycle, C*H* spirocycle), 3.61 (q, *J* = 5.4 Hz, 2H, C*H₂*NHCbz), 2.47 (m, 2H, C-C*H₂*-CH spirocycle), 2.14 (m, 2H, C-C*H₂*-CH spirocycle), 1.42 (s, 9H, 3 x C*H₃* Boc). ^{13}C NMR (75 MHz, $CDCl_3$) δ 158.7, 156.5, 156.3 (C=O Cbz, C=O Boc, C_{Ar}-O), 139.9, 136.5, 129.7, 128.7, 128.34, 128.30, 120.6, 113.8, 79.5 (*C*(CH_3)$_3$ Boc), 70.1, 68.1, 67.0, 61.9, 60.6, 41.2, 40.7, 30.3 (3 x *C*H_3 Boc), 28.5 (-*C*- spirocycle). LC-HRMS (ESI) calc. for $C_{28}H_{37}N_2O_6$ [M+H]$^+$: 497.2646; found: 497.2636.

3-[(4*R*)-2-Oxooxazolidin-4-yl]propanoic acid (CAS 2763643-93-0) (BB12)

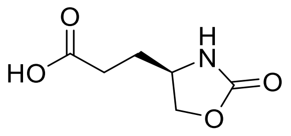

The compound was synthesized as first described in the patent WO 2022/049134.

(*R*)-1,1,1-Trifluoro-3-((4-methoxybenzyl)oxy)propan-2-ol (BB13)

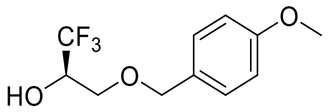

(4-Methoxyphenyl)methanol (777 mg, 5.625 mmol, 1.5 equiv.) was slowly added to a 0 °C solution of NaH (60 wt% in mineral oil, 450 mg, 11.25 mmol, 3 equiv.) in tetrahydrofuran (10 mL). After the reaction mixture had stirred at 0 °C for 60 minutes, (2R)-2-(trifluoromethyl)oxirane (prepared by hydrolytic kinetic resolution from racemate[63], 420 mg, 3.75 mmol) in 2 mL THF was added and allowed to warm to ambient temperature and stirred for 40 h. The reaction mixture was then cooled to 0 °C and quenched via the addition of water. The solution was separated between water and EtOAc. The aqueous phase was extracted with EtOAc two more times, and the combined organic phase was dried over sodium sulfate anhydrous, filtrated, and concentrated under reduced pressure. The title compound was purified via flash chromatography with gradient elution of 0-20% EtOAc in cyclohexane. The product was obtained as a pale-yellow oil (635 mg, 68%). Enantiomeric purity was confirmed via chiral HPLC. ^{1}H NMR (300 MHz, $CDCl_3$) δ 7.30 – 7.21 (m, 2H), 6.95 – 6.85 (m, 2H), 4.53 (d, *J* = 1.2 Hz, 2H), 4.12 (d, *J* = 5.7 Hz, 1H), 3.82 (s, 3H), 3.76 – 3.57 (m, 2H), 2.88 (s, 1H). ^{13}C NMR (75 MHz, $CDCl_3$) δ 159.58, 129.55, 129.05, 124.29 (q, *J* = 281.9 Hz) 114.00, 73.46, 69.56 (q, *J* = 31.0 Hz), 67.07, 67.04, 55.32. Spectral data are in accordance with the literature. [2]

3. Supporting Experiments

3.1. Mass-Spectroscopic Proof of Covalency

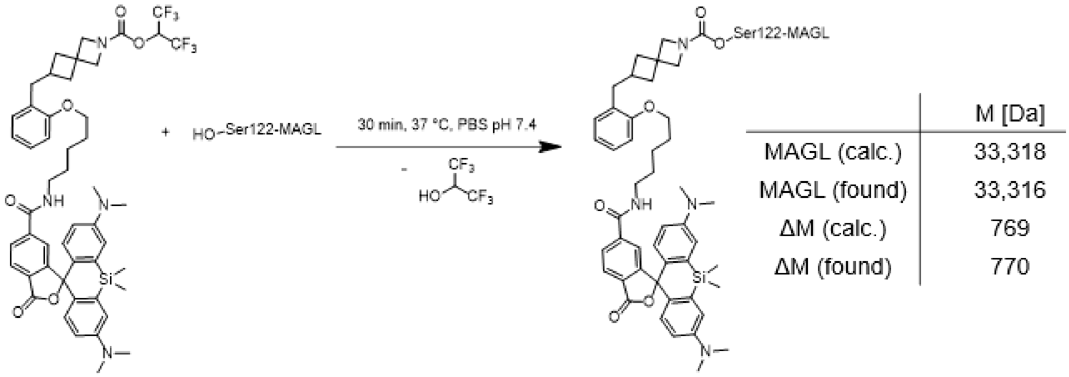

	M [Da]
MAGL (calc.)	33,318
MAGL (found)	33,316
ΔM (calc.)	769
ΔM (found)	770

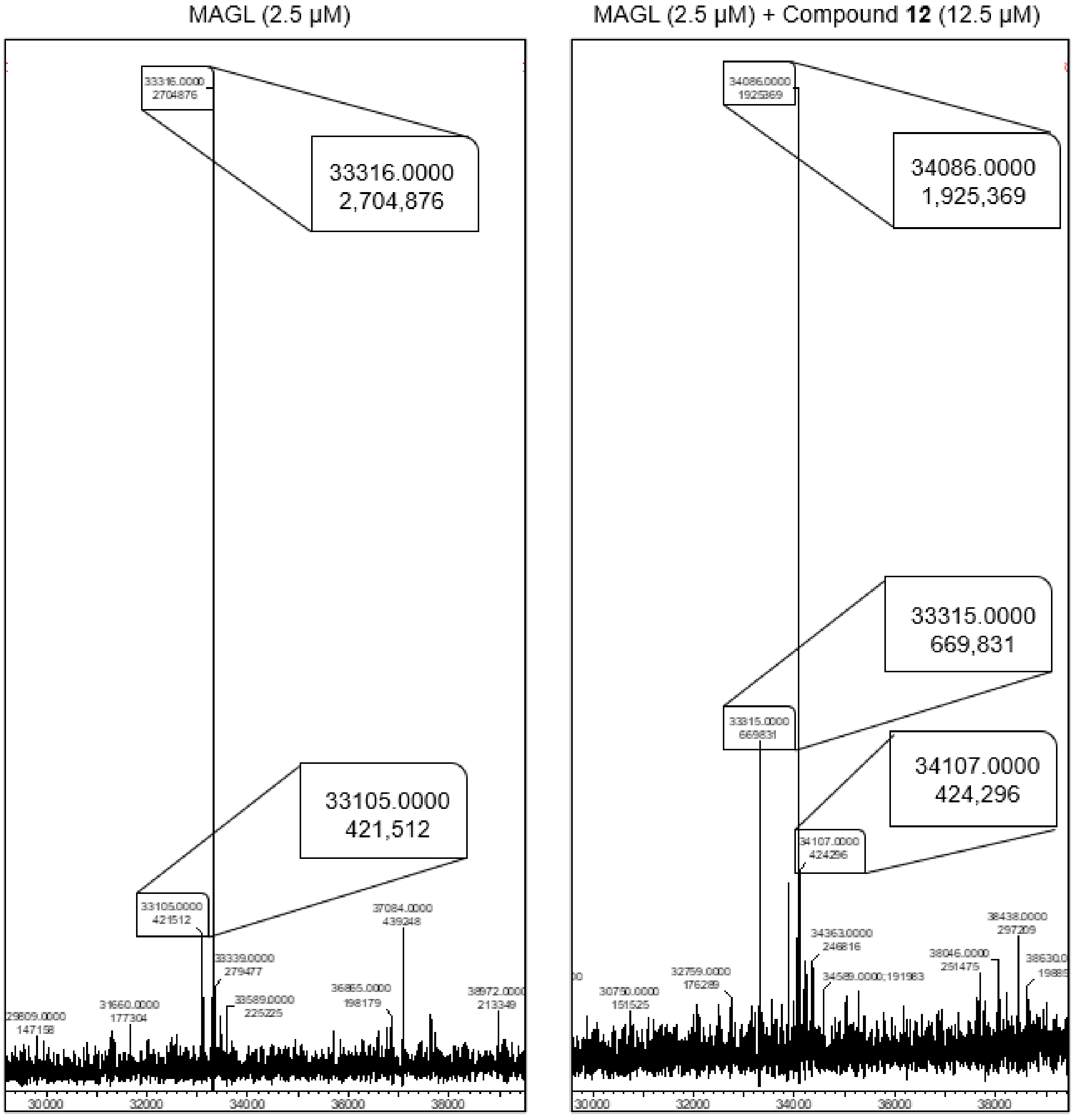

Figure S3. Mass-spectroscopic proof of covalent labeling of MAGL by **12.**

3.2. Confocal Fluorescence Imaging of Additional Cells

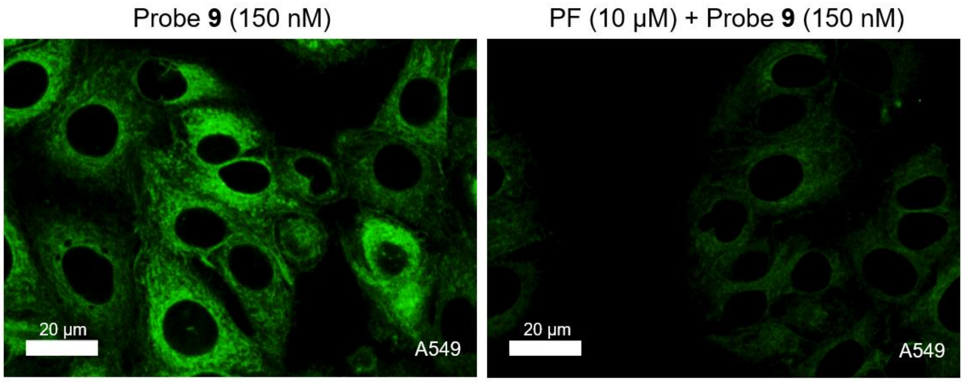

Figure S4. Confocal microscopy of A549 cells with Probe **9** (150 nM) shows intracellular staining. Blocking experiment with the MAGL-selective inhibitor PF-06795071 (PF) decreases fluorescence intensity without additional washing steps.

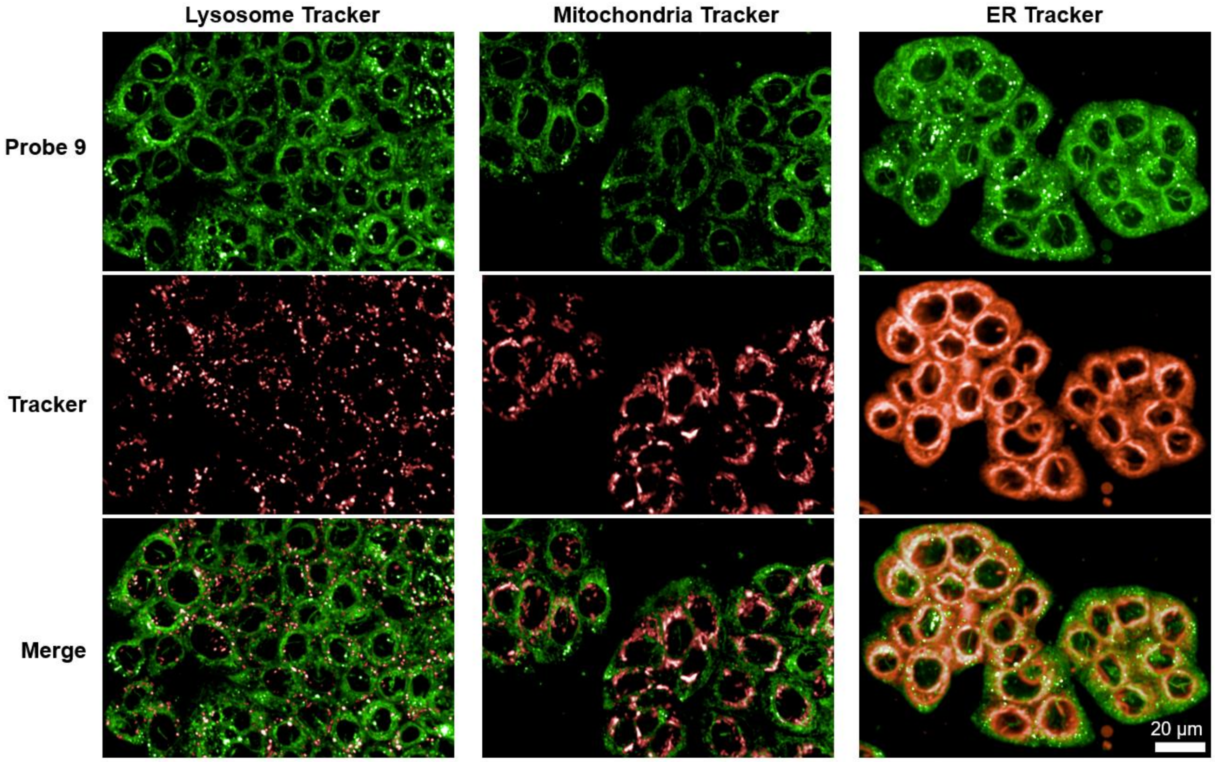

Figure S5. Confocal microscopy of HT-29 cells with Probe **9** (150 nM) shows no correlation with Lysosome Tracker (r = -0.57), weak correlation with Mitochondria Tracker (r = 0.41) and strong correlation with ER Tracker (r = 0.81). All trackers were obtained from Invitrogen.

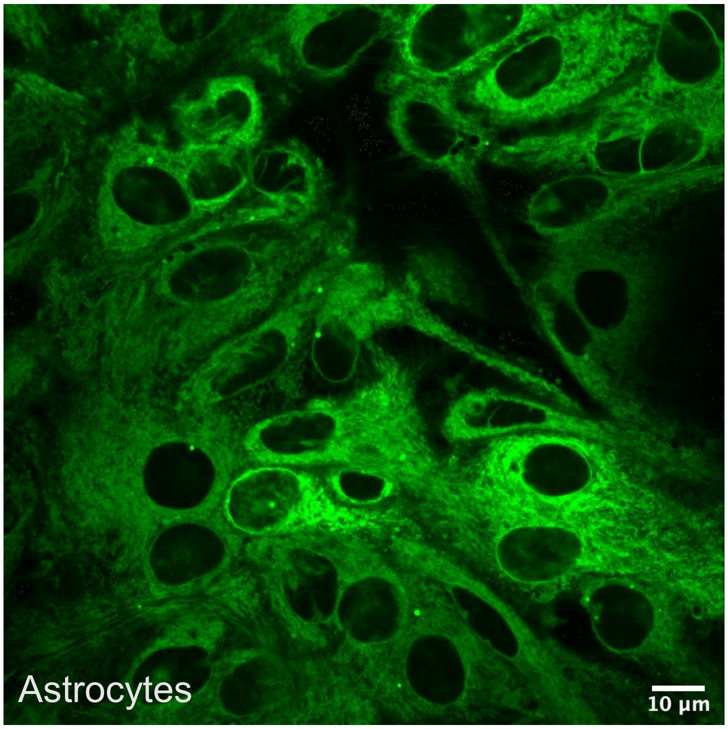

Figure S6. Primary astrocytes in culture are intensely stained with probe **12** (0.5 µM).

3.3. Photophysical Characterization

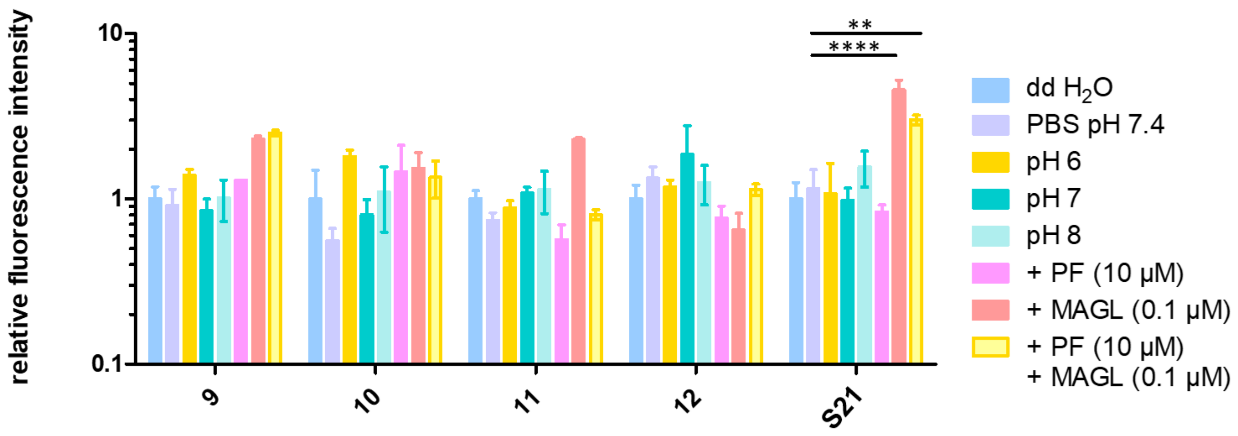

Figure S7. Influence of several conditions and analytes on fluorescence intensity of 9–12 and S21. The fluorescence intensity of probes **9–12** and **S21**, at 100 nM concentration each, in the respective channels was measured in triplicate and normalized to fluorescence in double distilled (dd) H_2O in at different ion strength, pH in the physiological range, in presence of MAGL enzyme and a prototypical MAGL inhibitor PF-06795071 (PF). Represented as mean ± standard deviation. Two-way ANOVA with Bonferroni post-test was performed. ** indicates $p < 0.01$ and **** indicates $p < 0.0001$. For probes **9-12**, no significant changes were observed.

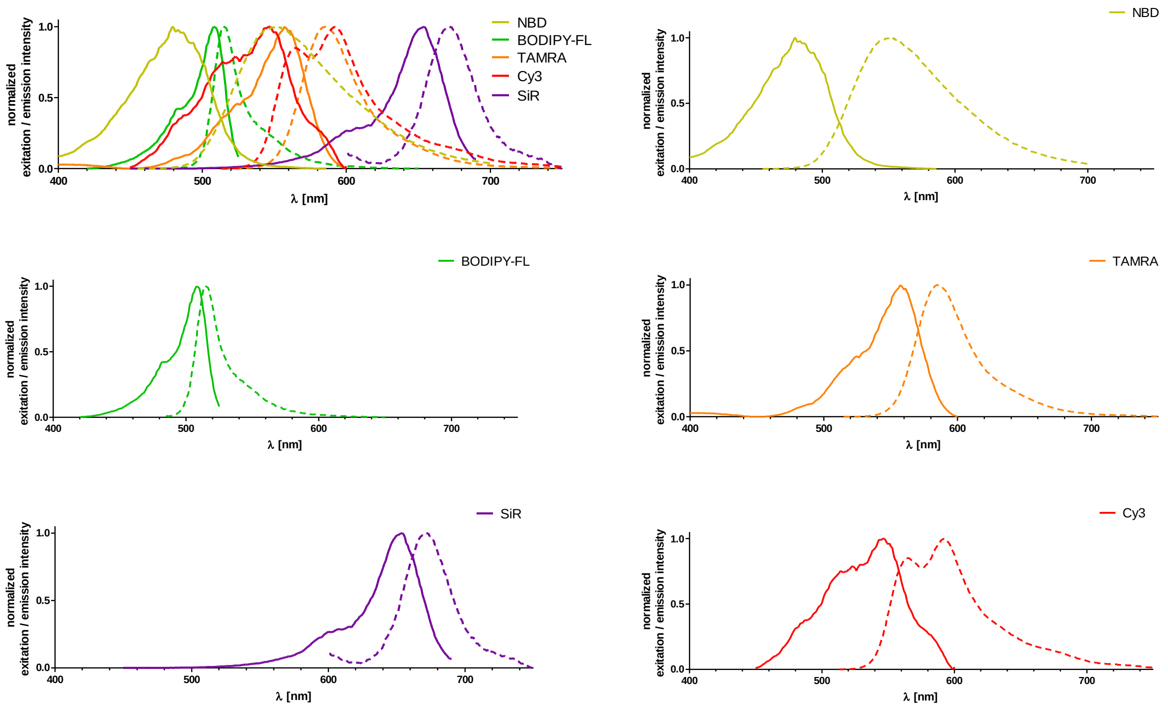

Figure S8. Fluorescence excitation and emission spectra of the utilized fluorescent reporters in PBS pH 7.4. Spectral differences between probes carrying the same fluorophore were negligible.

Table S6. Fluorescence excitation, emission maxima, and Stokes-shift in PBS pH 7.4 for the used fluorophores, respectively. differences between probes with the same fluorophore were negligible.

	λ_{max} Excitation [nm]	***λ_{max} Emission [nm]***	***Stokes-shift [nm]***
**NBD**	479	554	75
**BODIPY-FL**	508	514	6
**TAMRA**	557	585	28
**Cy3**	547	592	45
**SiR**	654	673	19

4. Experimental Details of Biochemical Assays and Crystal Structure

In Vitro MAGL Inhibition Assay

Compounds were profiled for MAGL inhibitory activity by determining the enzymatic activity by following the hydrolysis of the natural substrate 2-arachidonoylglycerol (2-AG), resulting in arachidonic acid, which can be followed by mass spectrometry. The 2-AG assay was carried out in 384 well assay plates (PP, Greiner Cat# 784201) in a total volume of 20 µL. Compound dilutions were made in 100% DMSO (VWR Chemicals 23500.297) in a polypropylene plate in 3-fold dilution steps to give a final concentration range in the assay from 12.5 µM to 0.8 pM. 0.25µL compound dilutions (100% DMSO) were added to 9 µL MAGL in assay buffer (50 mM TRIS (GIBCO, 15567-027), 1 mM EDTA (Fluka, 03690-100 mL), 0.01% (v/v) Tween. After shaking, the plate was incubated for 15 min at RT. To start the reaction, 10 µL 2-arachidonoylglycerol in assay buffer was added. The final concentrations in the assay were 50 pM MAGL and 8 µM 2-arachidonoylglyerol. After shaking and 30 min incubation at RT, the reaction was quenched by the addition of 40µL of ACN containing 4 µM of d8-arachidonic acid. The amount of arachidonic acid was traced by an online SPE system (Agilent Rapidfire) coupled to a triple quadrupole mass spectrometer (Agilent 6460). A C18 SPE cartridge (G9205A) was used in an ACN/water liquid setup. The mass spectrometer was operated in negative electrospray mode following the mass transitions 303.1 → 259.1 for arachidonic acid and 311.1 → 267.0 for d8-arachidonic acid. The activity of the compounds was calculated based on the ratio of intensities [arachidonic acid / d8-arachidonic acid].

NanoBRET Assay

This assay was performed as first described before.[39]
Compounds were profiled by a novel cellular MAGL NanoBRET™ Target Engagement assay, which measures the apparent affinity of test compounds by competitive displacement of a Roche-developed Tracer (WO2021058443, Example 21)[21], reversibly bound to a NanoLuc® luciferase MAGL fusion protein in cells.

A HEK293A stable cell line expressing NanoLuc_MAGL was established at Roche and assigned as HEK293A_NanoLuc_MAGL clone #43. A fixed concentration of tracer is added to cells expressing the NanoLuc®_MAGL fusion protein to generate a BRET reporter complex. The introduction of competing compounds results in a dose-dependent decrease in NanoBRET™ energy transfer, which allows quantification of the intracellular affinity of the target protein for the test compound.

HEK293A_NanoLuc_MAGL clone #43 cells were cultured in DMEM (Gibco 31966) with 10% FBS and 800 ug/ml G418. The NanoBRET assay was performed by seeding 5000 cells/well in Costar low-volume white 384-well plate in assay buffer (1% FBS in HBSS), followed by adding 50 nM final concentration of tracer and serial dilutions of test compounds. After 2 hours of incubation, Nanoluciferase substrates and extracellular inhibitors were added following the manufacturer's protocol (Promega, #N2520), and the NanoBRET signaling were quantified with the Paradigm (Molecular Devices).

Crystal Structure

Crystallization, data collection and structure determination of the human MAGL in complex with probe 9

Human MAGL protein with mutations introduced at positions Lys36Ala, Leu169Ser and Leu176Ser[73] was produced by Cepter Biopartners LLC (Nutley, USA) and purified as described in the literature[74].

For crystallization the protein was concentrated to 9.4 mg/mL. Crystallization trials were performed in sitting drop vapor diffusion setups at 21 °C. Crystals appeared within 2 days out of 0.1M MES pH 6.5, 8 % (v/v) 1-Propan, 12 % (w/v) PEG MME 5K. The structure of hMAGL (K36A, L169S, L176S) with probe 9 was obtained by soaking crystals for 110 h in crystallization solution supplemented with 10 mM inhibitor dissolved in DMSO. Long soaking times were required to obtain full occupancy of the compound. In the structure side chain of Cys208 appears to be modified based on the electron density map but identity of the modification could not be clarified.

For data collection crystals were flash cooled at 100 K with 20 % ethylene glycol added as cryo-protectant. X-ray diffraction data were collected at a wavelength of 0.88560 Å using an Eiger2X 16M detector at the beamline ID23-1 of the ESRF (Grenoble, France). Data have been processed with XDS[75] and scaled with SADABS (BRUKER). The crystals belong to space group $C222_1$ with cell axes of a= 88.89 Å, b= 127.24 Å, c= 62.20 Å and diffract to a resolution of 1.41Å. The structure was determined by molecular replacement with PHASER[76] using the coordinates of PDB entry 3pe6 as search model. Difference electron density was used to place the inhibitors. The structure was refined with programs from the CCP4 suite[77] and PHENIX[78]. Manual rebuilding was done with COOT.[79] The coordinates of the structure were deposited in the PDB under the accession code 9G4M. Data collection and refinement statistics are summarized in Table S7.

Table S7. Data collection and refinement statistics. Values in parentheses are for highest-resolution shell.

	***h*MAGL compound probe 9**
**Data collection**	
Space group	$C222_1$
Cell dimensions	
a, *b*, *c* (Å)	88.89, 127.24, 62.20
α, β, γ (°)	90, 90, 90
Resolution (Å)	1.41 (1.50-1.41)
Total reflections<br>Total unique reflections<br>R_{sym}	689285<br>68084<br>0.093 (0.99)
I / σI	8.97 (0.90)
CC(1/2)	0.997 (0.552)
Completeness<br>Redundancy	99.9 (99.6)<br>10.36 (10.11)
**Refinement**	
Resolution (Å)	36.16 – 1.41
No. reflections	67897
R_{work} / R_{free}	15.9/18.5
No. atoms	
Protein	2305
Water<br>Ligand	210<br>49
B-factors	
Protein	26.78
Water<br>Ligand	43.48<br>25.68
R.m.s. deviations	
Bond lengths (Å)<br>Bond angles (°)	0.005<br>0.838
PDB code	9G4M

ABPP and in gel staining

Mouse brain sample preparation. Mouse tissues were isolated according to guidelines approved by the ethical committee of Leiden University. Male mouse brain (27 weeks old) was homogenized with glass beads (2x 1 min, bullet blender, speed 8) using cold lysis buffer (20 mM Hepes pH 7.2, 1 mM $MgCl_2$, 2 U/mL Benzonase). The membrane and cytosol were separated by centrifugation. The membrane fraction was resuspended in Hepes/DTT buffer. Protein concentration was determined with Bradford Assay, and samples were diluted to a final concentration of 2.0 mg/mL. Samples were snap-frozen in liquid nitrogen and stored at -80 °C until further use.

Competitive ABPP. Lysate (19 µL, 2 µg/µL) was thawed on ice. For comparative ABPP (irreversible probes), 1 µL of probe (20x stock in DMSO) or pure DMSO was added to the sample, vortexed briefly, and incubated for 30 minutes at RT. For competitive ABPP (reversible probes), 0.5 µL of the reversible probe (40x stock in DMSO) or pure DMSO (as vehicle) was added to the sample, vortexed briefly, and incubated for 30 minutes at RT. Subsequently, 0.5 µL MB064 (40x stock in DMSO) was added to the proteome sample, vortexed briefly, and incubated for 10 minutes at RT, after which 0.5 µL FP-Bodipy-FL (40x stock in DMSO) was added, vortexed briefly and incubated for 10 minutes at RT. The final volume was 20 - 20.5 µL (5 - 7.5% DMSO). The reaction was quenched by the addition of 7.5 µL of 4*Laemmli-buffer (final concentrations: 60 mM Tris (pH 6.8), 2% (w/v) SDS, 10% (v/v) glycerol, 1.25% (v/v) β-mercaptoethanol, 0.01% (v/v) bromophenol blue). 10 µL (14 µg protein) of the quenched reaction mixture was resolved on 10% acrylamide SDS-PAGE (180 V, 75 min). Fluorescence was measured using a Biorad ChemiDoc MP system (fluorescence channels Cy2, Cy3, Cy5). Labeling of the respective proteins via MB064 and FP-BODIPY was visualized in two separate channels. Gels were then stained using coomassie staining and imaged for protein loading control.
Final concentrations

- MBP = 14 µg
- MB064 = 250 nM
- FP-Bodipy = 100 nM

PBMC preparations. Peripheral blood mononuclear cells (PBMCs) were prepared from buffy coats obtained from the Blutspendedienst Zentralschweiz SRK after written informed consent from the donors according to the Swiss Guidelines (Blutspende SRK Schweiz Generalkonsent 2019/3_ Version 1.0). Briefly, PBMCs were isolated by Ficoll-Paque (1.077 g/mL density) (17–1440–02; GE Healthcare Bio-sciences) density gradient centrifugation at 400 × g at 18°C for 35 min. Cells were washed several times using 1× PBS and frozen in CryoStor® (C2874; Sigma) until further use.

Activity-based protein profiling (ABPP). PBMCs were thawed and washed with 1 x PBS, and equal amounts of cells were exposed to DMSO (D2650; Sigma-Aldrich) or MAGL inhibitor PF-06795071 in DMSO for 30 min at room temperature with concentrations ranging from 1 nM to 1000 nM. Cells were washed with 1 x PBS and lysed in ice-cold lysis buffer (ab156035; Abcam), lysates were centrifuged at 14,000 x g for 15 minutes at 4°C and supernatants were collected. For ABPP experiments, cell lysates containing equal amounts of protein were incubated with the appropriate concentration of activity-based probe for 30 min at room temperature. The reaction was quenched for 30 min by adding SDS-PAGE loading buffer (NP0007, Thermo). Samples were then separated by SDS-PAGE and bands were detected ChemiDoc Imager (Bio-Rad Laboratories). Following detection, gels were stained with SimplyBlue™ SafeStain (LC6060, Thermo) for 1 hour with gentle agitation and excess stain was removed by destaining in ddH2O for 1 h with gentle agitation. Gels were imaged and protein bands were quantified using Image Lab software (Bio-Rad Laboratories).

HT-29 Cellular Imaging

Fluorescence confocal microscopy. HT-29 cells were cultured in DMEM medium (Gibco) supplemented with 10% heat-inactivated FBS in a humidified atmosphere at 37°C and 5% CO_2. The imaging experiments were conducted in a 384-well microplate format (PhenoPlate, Revvity). Fluorescent probes were stored as 10mM stocks in DMSO and pre-diluted before applying to cells. Experiments were performed in at least two independent experiments. Cells were dispensed at a density of 2500 cells/well (40 µL) onto a microplate. After incubation for 48 h, the medium was replaced with serum-free conditions without Phenol red (20 µL). Fluorescent probes were added (10 µL) during the imaging process after image capturing of starting conditions and tested at different concentrations (150 nM, 250 nM, 500 nM). In the case of blocking experiments, cells were incubated with an inhibitor (PF-06795071, 10 µM) for 90 min before probe administration.

Localization experiments. Cells were plated onto a microplate at a density of 2500 cells/well (40 µl) and incubated for 48 h. After the replacement of medium to serum-free conditions without Phenol red (20 µL), different cell compartments were stained using ER-TrackerTM Red (Invitrogen) or Mito-TrackerTM Deep Red (Invitrogen) for 30 min, followed by a washing step with medium. Cells were incubated for 30 min with fluorescent probes (10 µL, 150 nM) and washed three times with medium (50µl) before live cell confocal imaging. Samples without fluorescent probe but with ER-tracker, fluorescent probe plus or without ER-tracker served as controls.

Confocal live cell image acquisition. Confocal live cell imaging was performed using the Opera Phenix™ High Content Screening System (Revvity). The probe fluorescence was monitored by kinetic measurements of 10 min with a break for probe administration. The fluorescence of one image per sample was captured at each time point. Probe detection was realized using the appropriate laser for excitation and filter for fluorescence emission. Image acquisition parameters, including laser power, offset, and gain settings, were kept constant. All cell imaging measurements were done using water immersion objectives (63xWater-NA 1.15, 40xWater-NA 1.1).

Hippocampal Neurons Staining

Preparation of primary neuron cultures. For neuron culture, glass coverslips (19 mm, #1.5 Epredia Deckgläser, CB00190RAC20MNZ0) were coated with PLL (Sigma-Aldrich, P4707; 1:12 dilution) in phosphate-buffered saline (PBS) for 2h at 37 °C. Hippocampal neurons were then prepared from P0 wild-type mice as previously described [15] and plated at a density of 200k cells per coverslip in neuronal medium (penicillin [200 U/mL]/streptomycin [200 µg/mL[[Gibco, 15140-130], 2 mM GlutaMAX [Gibco, 35050-038], 2 % B27 [Gibco life technologies 17504-044] in Neurobasal-A), and grown for 2-3 weeks in an incubator at 37 °C and 5% CO_2. The medium was replaced once the day after plating.

Fluorescent labeling and image acquisition. Fluorescent dyes were added to the cell culture media (final concentrations as indicated) and incubated for 15 min at 37 °C and 5% CO_2. As a negative control, coverslips were treated with 10 µM PF-06795071 for 2h at 37 °C and 5% CO_2 prior to fluorescent labeling. Neurons were subsequently washed in PBS twice, mounted on a grooved microscope slides in PBS, and imaged live on a Zeiss LSM 710 confocal laser scanning microscope for a maximum of 30 min. Images were then analyzed using FIJI.

MAGL Protein Mass Spectrometry

Intact MAGL protein was incubated with the indicated probe concentrations at 37 °C in PBS pH 7.4 for 30 min. 30 µL test samples were combined with 10 µL MeCN and put on ice until further analysis. The protein was analyzed using a Waters H-class instrument equipped with a quaternary solvent manager, a Waters sample manager-FTN, a Waters PDA detector and a Waters column manager with an Acquity UPLC protein BEH C4 column (300 Å, 1.7 µm, 2.1 mm x 50 mm). Proteins were eluted with a flow rate of 0.3 mL/min with 80°C column temperature. The following gradient was used: A: 0.01% FA in H2O; B: 0.01% FA in MeCN. 5-95% B 0-6 min. Mass analysis was conducted with a Waters XEVO G2-XS QTof analyzer. Raw data was deconvoluted with MaxEnt 1.

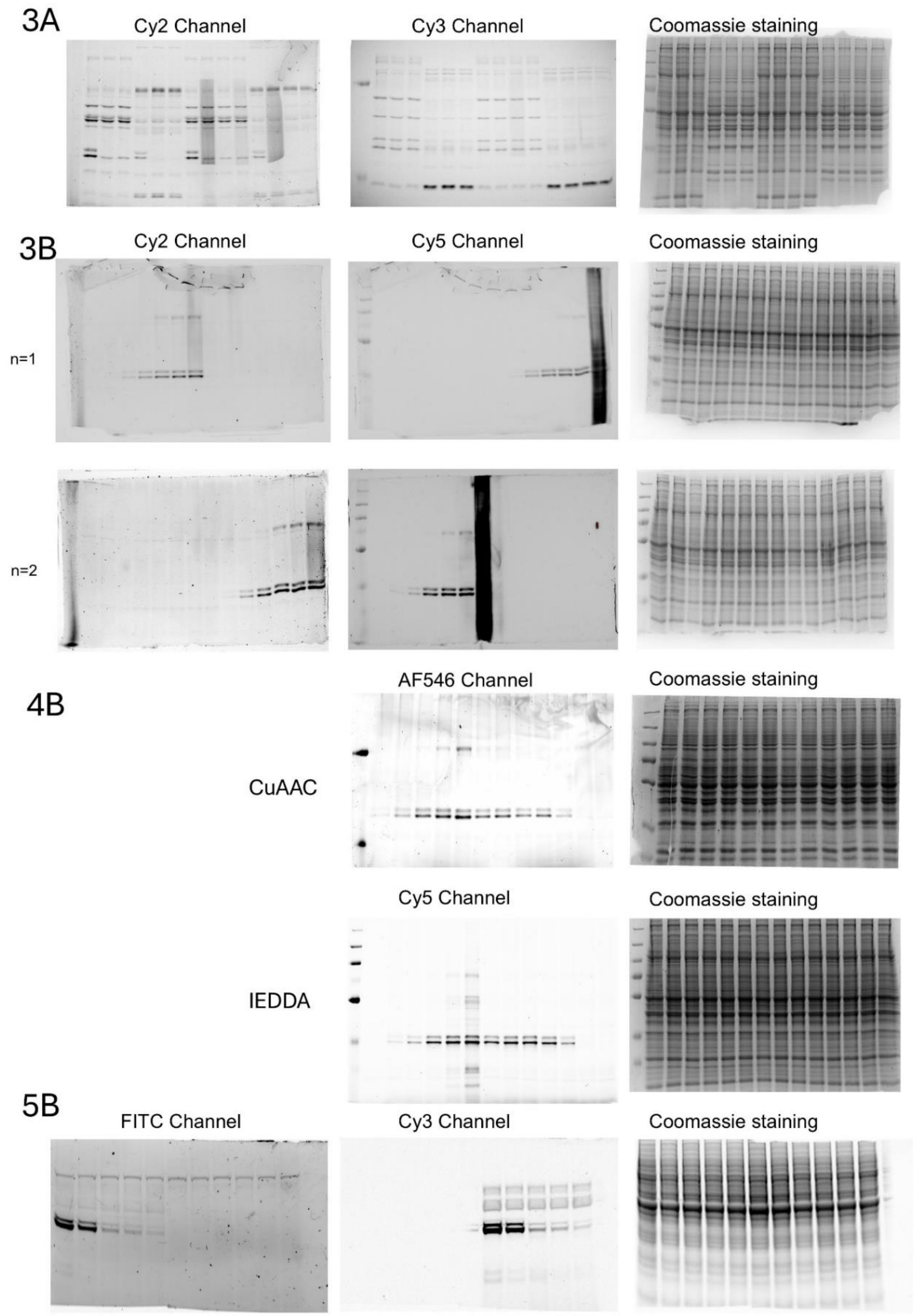

Figure S9. Unprocessed and uncut SDS-PAGE scans of the respective figures 3, 4 and 5 in the manuscript. The used channel settings (BioRad) for fluorescent image acquisition are indicated. One row represents one SDS-PAGE gel.

5. NMR Spectra of Key Probes 8–14

Probe 8

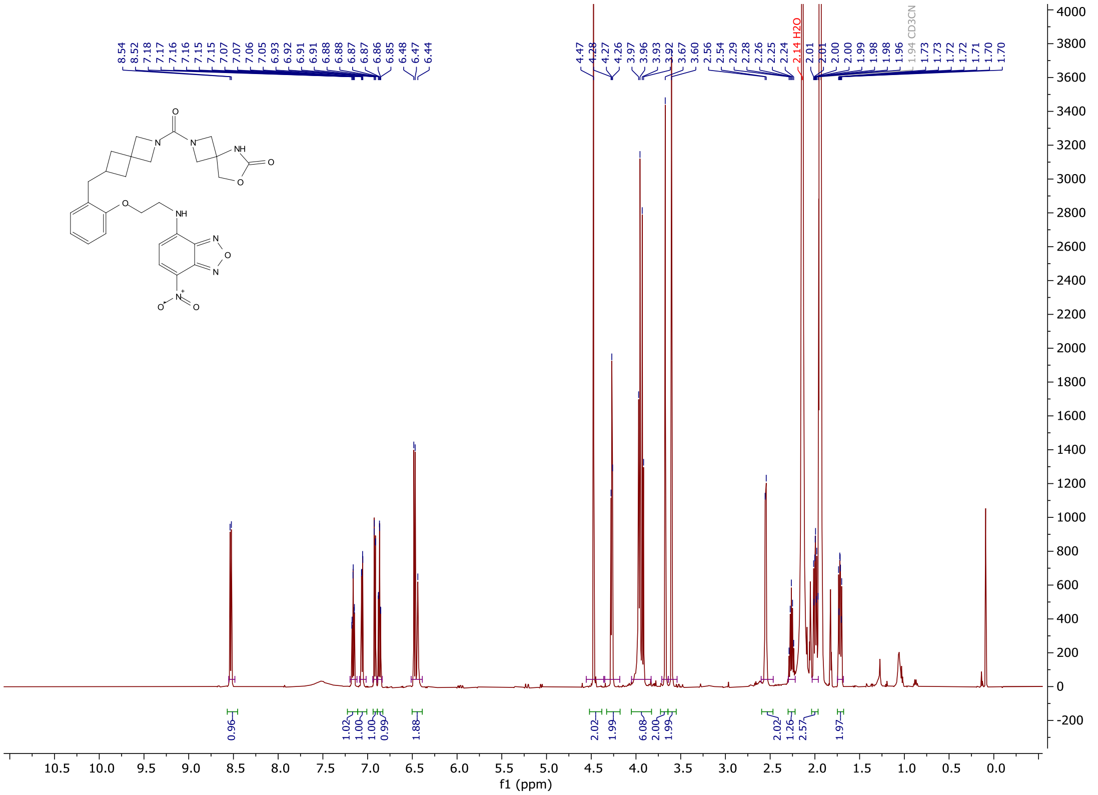

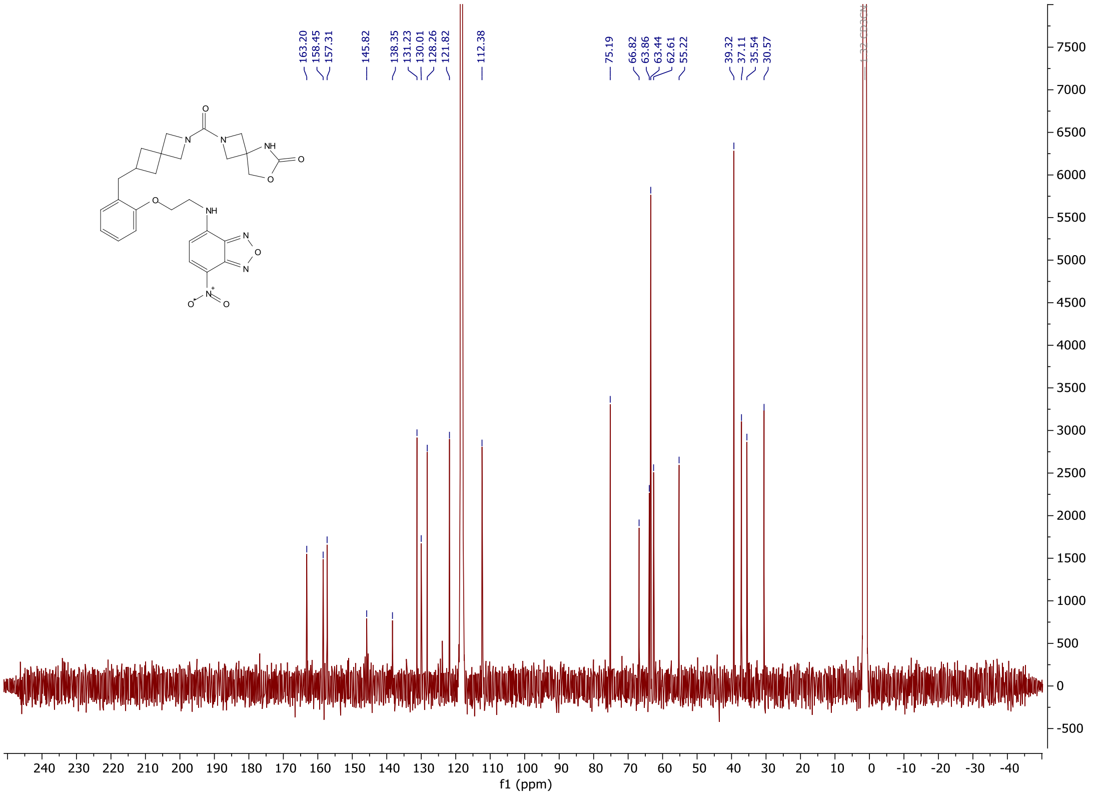

Probe 9

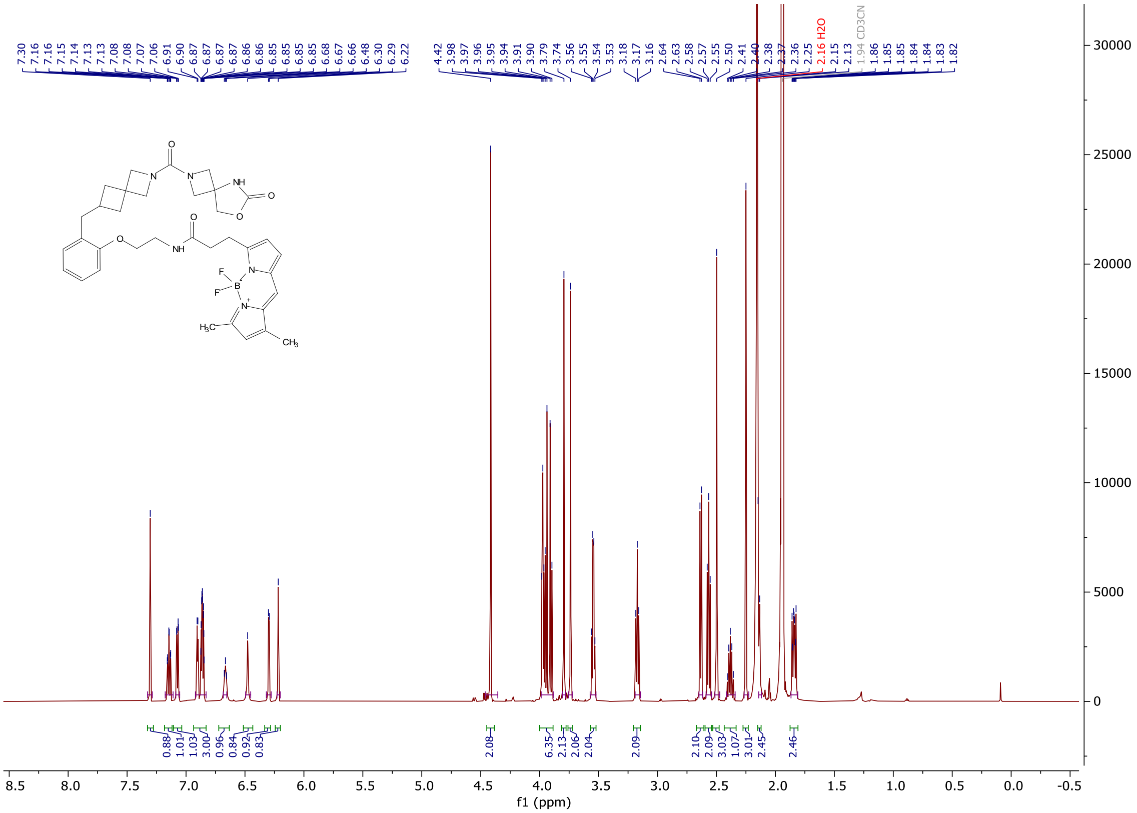

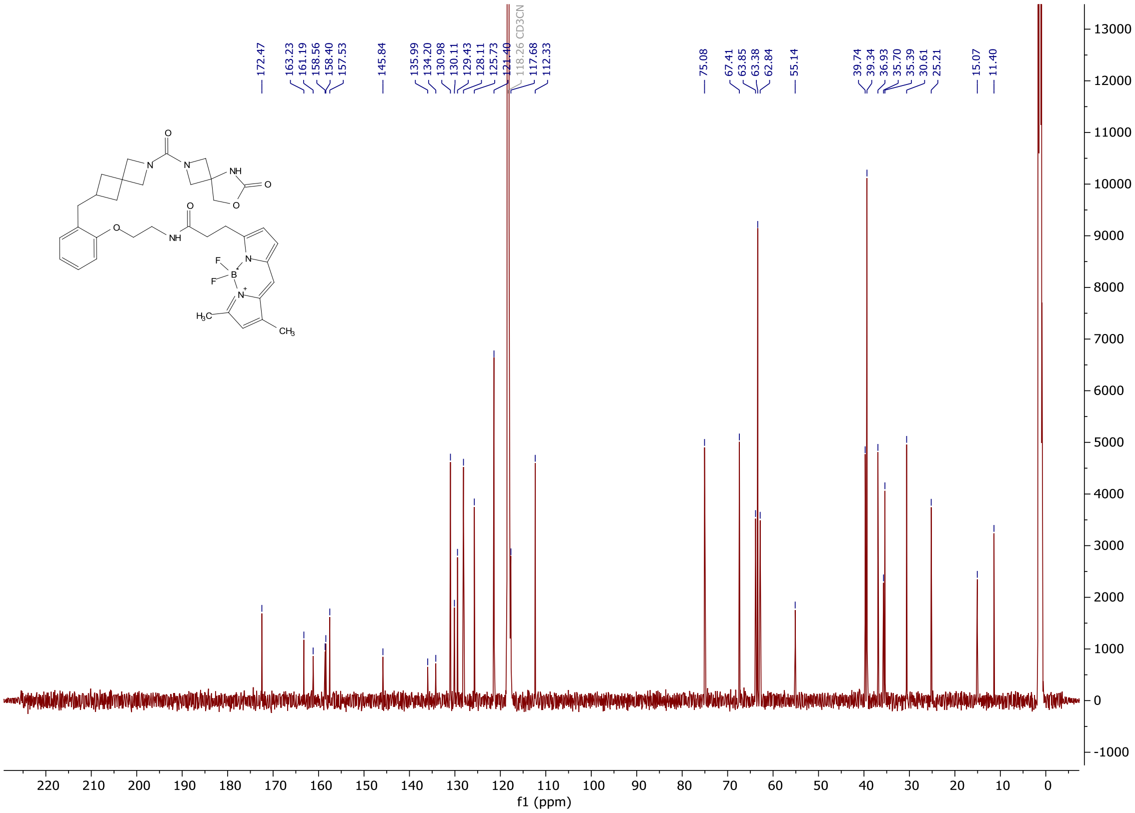

Probe 10

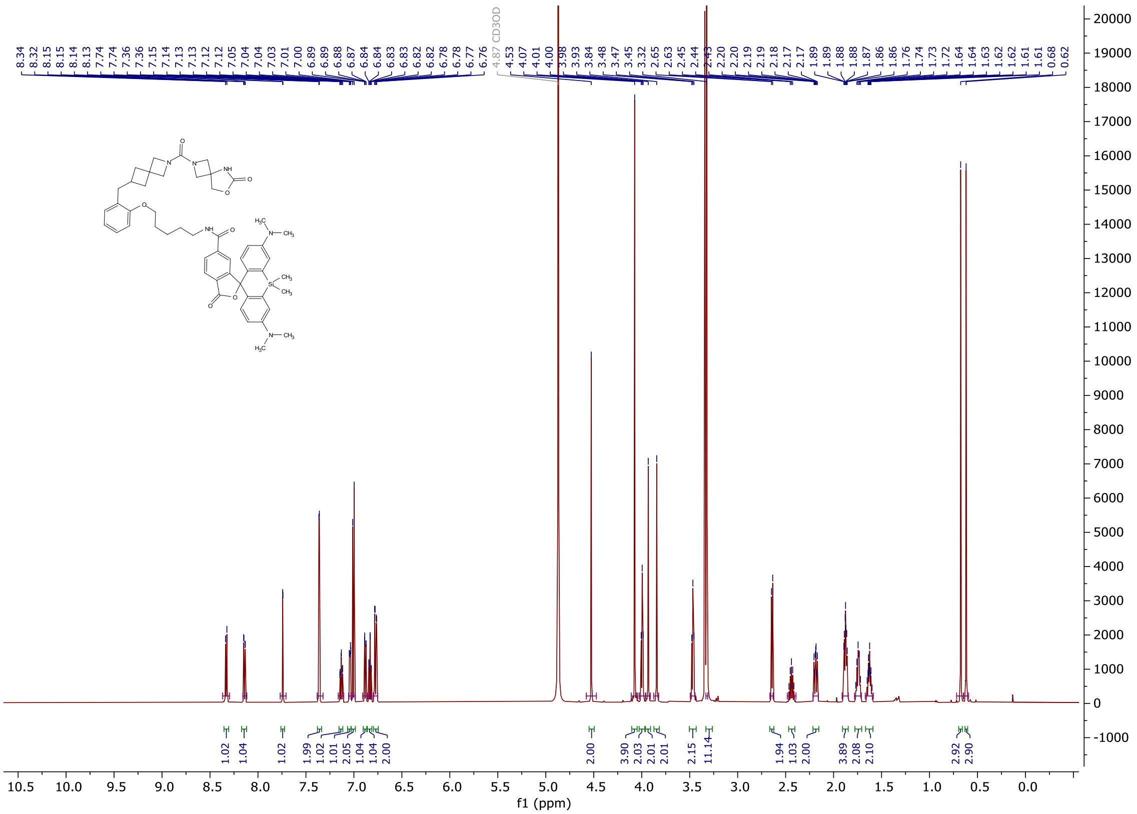

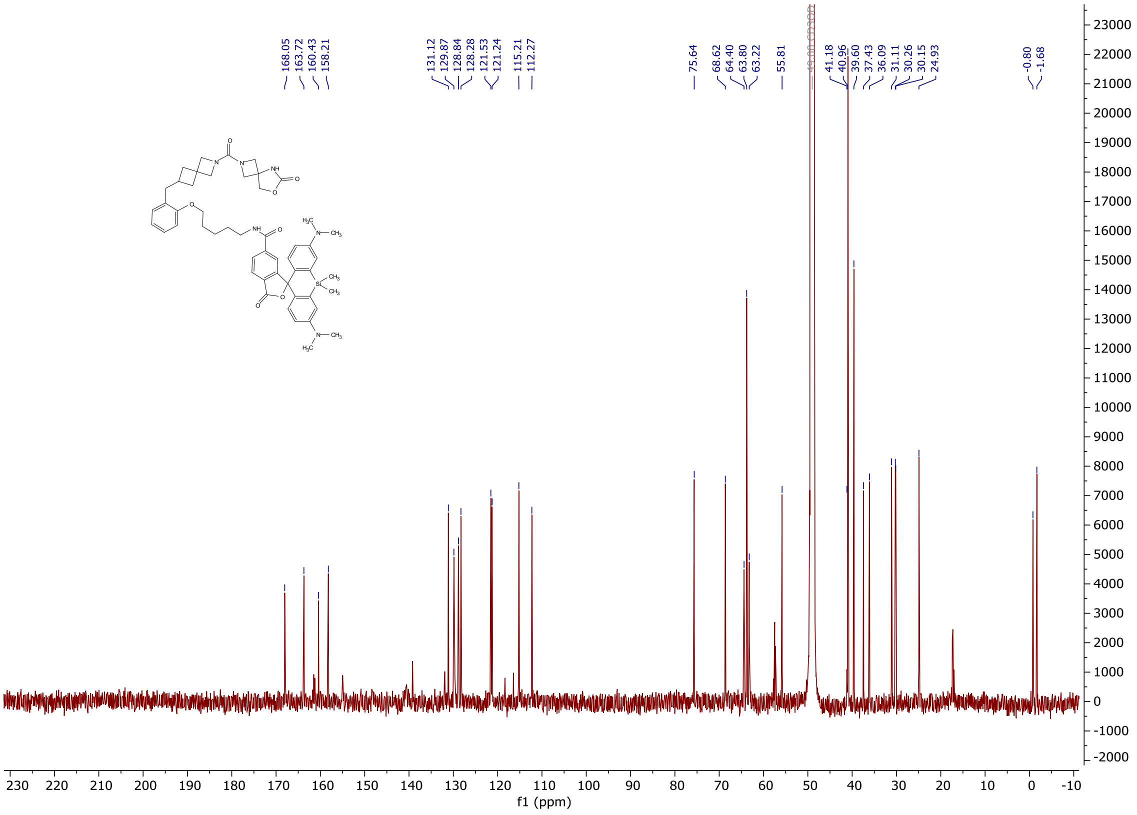

Probe 11

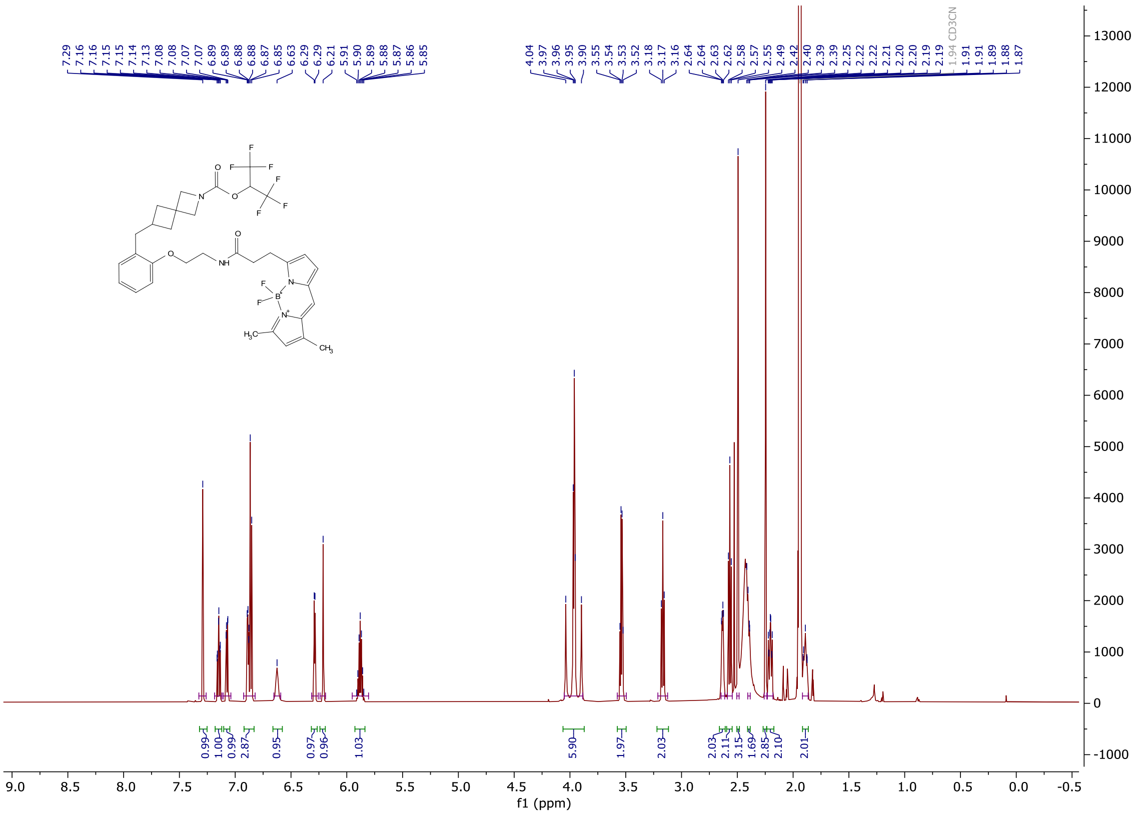

7.29 7.16 7.15 7.15 7.14 7.13 7.08 7.08 7.07 7.07 6.89 6.89 6.88 6.88 6.87 6.85 6.63 6.29 6.29 6.21 5.91 5.90 5.89 5.88 5.87 5.86 5.85
4.04 3.97 3.96 3.95 3.90 3.55 3.54 3.53 3.52 3.18 3.17 3.16 2.64 2.64 2.63 2.62 2.58 2.57 2.55 2.49 2.42 2.40 2.39 2.39 2.25 2.22 2.22 2.21 2.20 2.20 2.19 2.19 1.94 CD3CN 1.91 1.91 1.89 1.88 1.87
0.99 1.00 0.99 2.87 0.95 0.97 0.96 1.03 5.90 1.97 2.03 2.03 2.11 1.15 1.69 2.85 2.10 2.01
f1 (ppm)

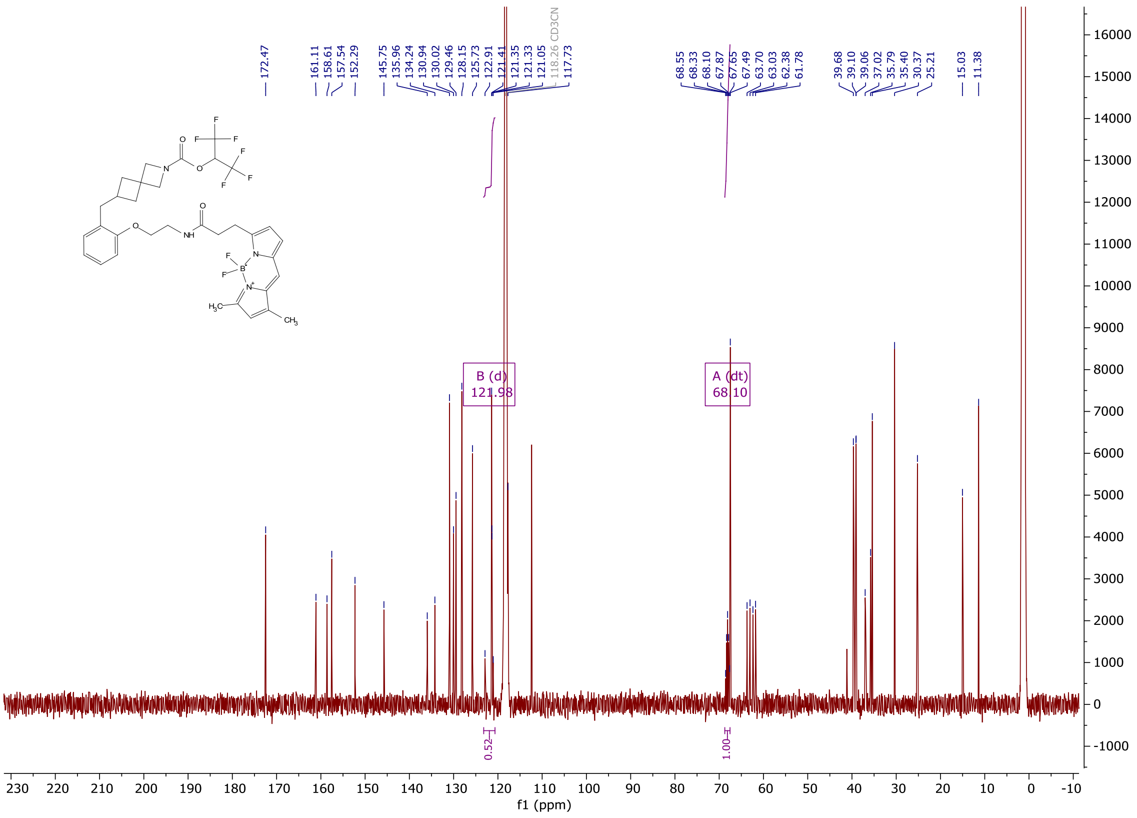

172.47 161.11 158.61 157.54 152.29 145.75 135.96 134.24 130.94 130.02 129.46 128.15 125.73 122.91 121.41 121.35 121.33 121.05 118.26 CD3CN 117.73
68.55 68.33 68.10 67.87 67.65 67.49 63.70 63.03 62.38 61.78 39.68 39.10 39.06 37.02 35.79 35.40 30.37 25.21 15.03 11.38
B (d) 121.98
A (dt) 68.10
0.52
1.00
f1 (ppm)

Probe 12

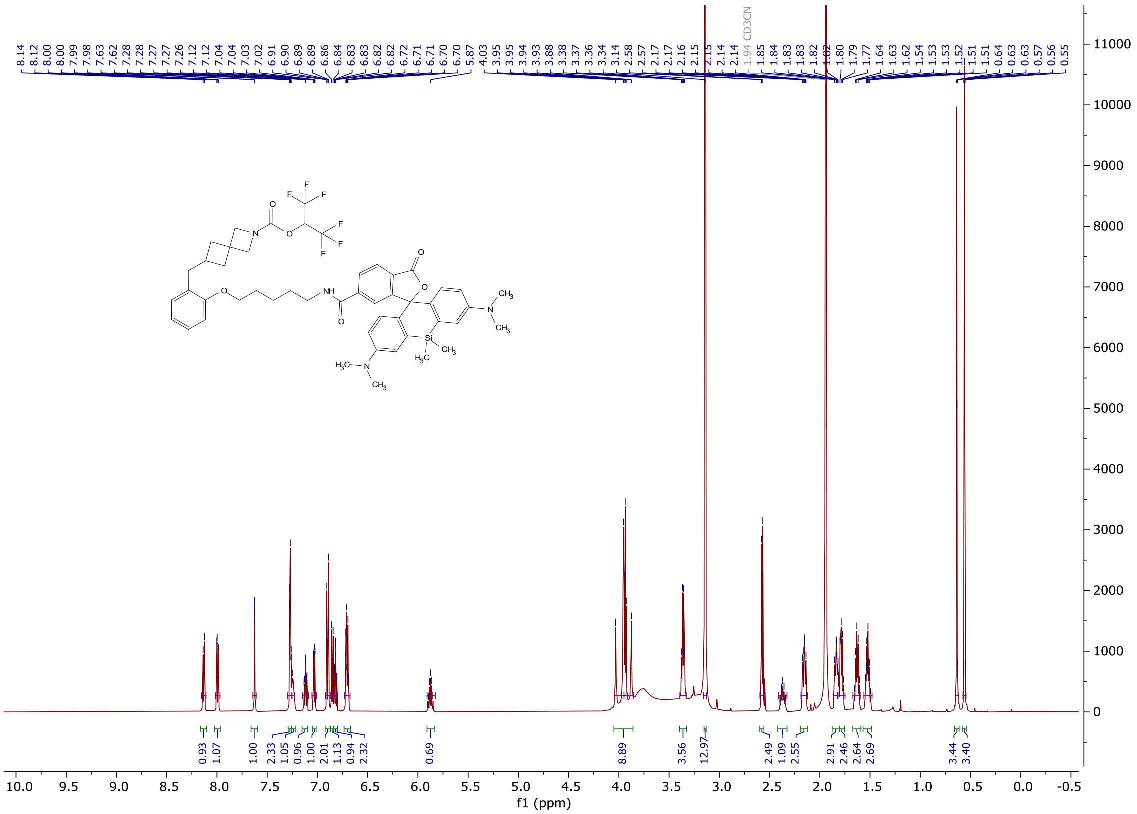

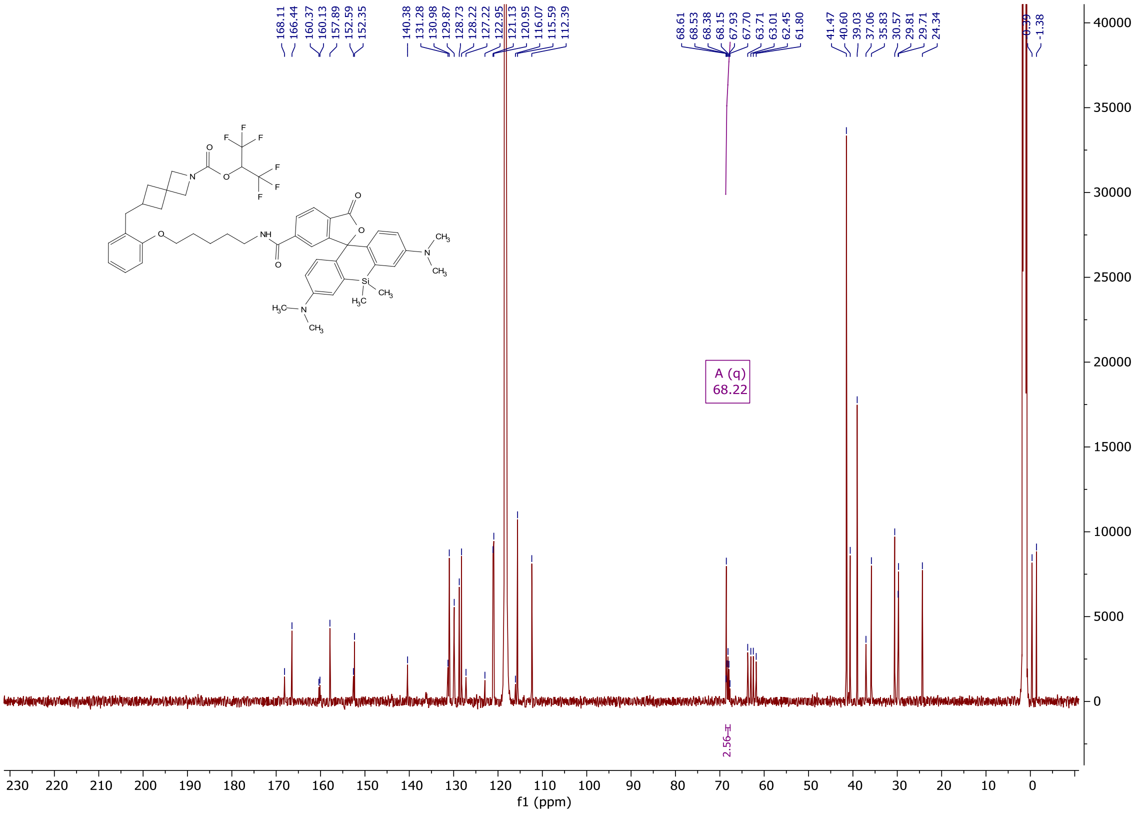

Probe 13

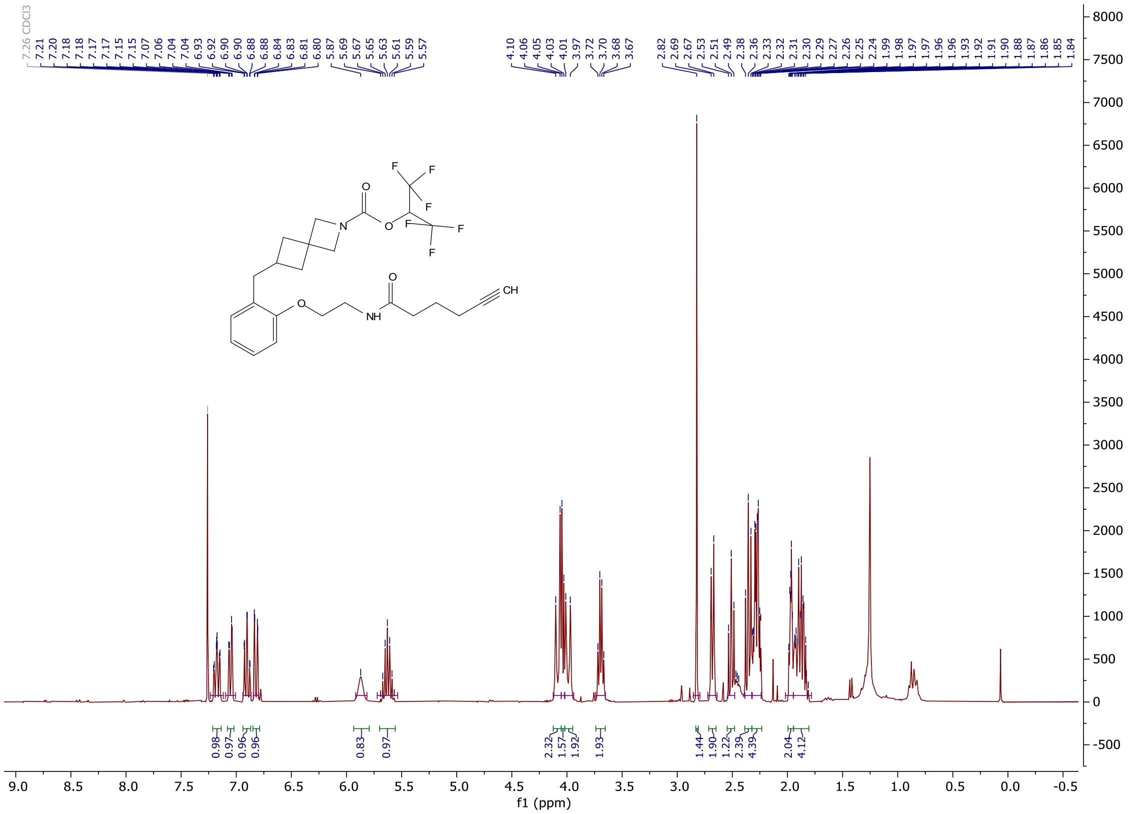

7.26 CDCl3
7.21 7.20 7.18 7.18 7.17 7.17 7.15 7.07 7.06 7.04 7.04 6.93 6.92 6.90 6.90 6.88 6.88 6.84 6.83 6.81 6.80 5.87 5.69 5.67 5.65 5.63 5.61 5.59 5.57
4.10 4.06 4.05 4.03 4.01 3.97 3.72 3.70 3.68 3.67
2.82 2.69 2.67 2.53 2.51 2.49 2.38 2.36 2.33 2.32 2.31 2.30 2.29 2.27 2.26 2.25 2.24 1.99 1.98 1.97 1.97 1.96 1.93 1.92 1.91 1.90 1.88 1.87 1.86 1.85 1.84
O
F
N
NH
CH
0.98 0.97 0.96 0.96 0.83 0.97 2.32 1.57 1.92 1.93 1.44 1.90 1.22 2.39 4.39 2.04 4.12
f1 (ppm)

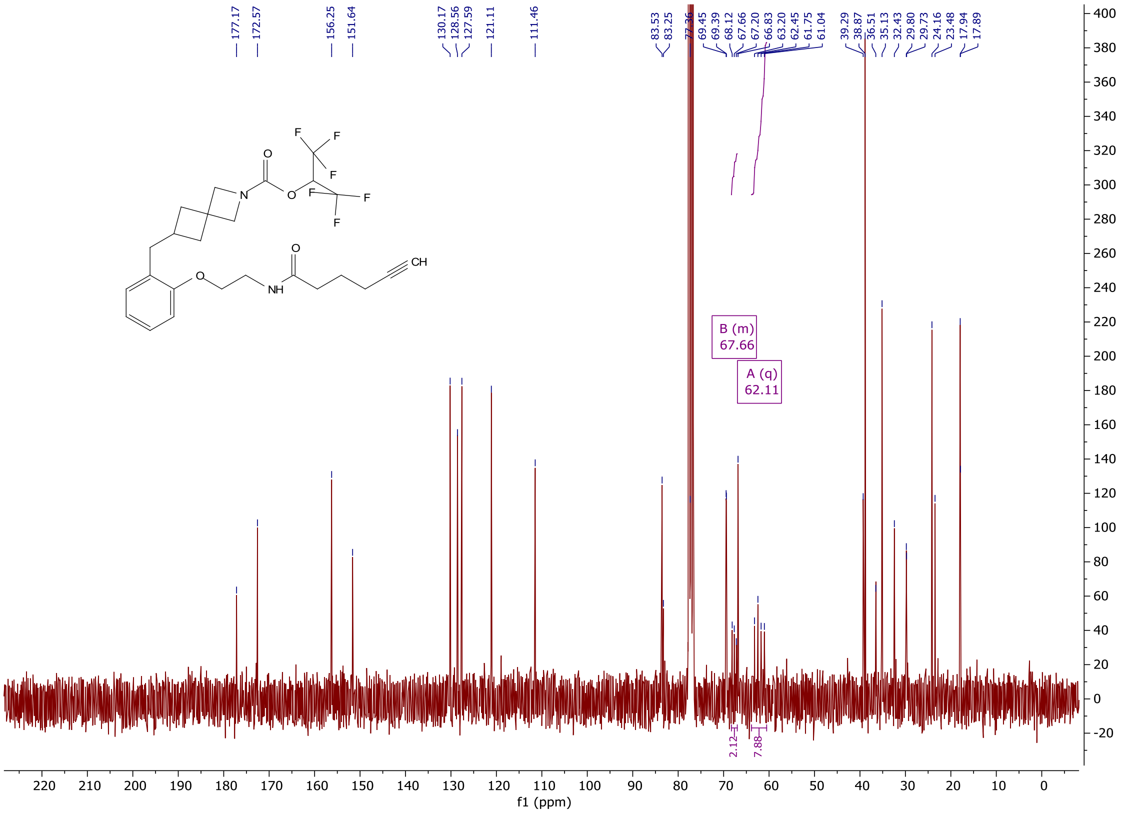

177.17 172.57 156.25 151.64 130.17 128.56 127.59 121.11 111.46 83.53 83.25 77.36 69.45 69.39 68.12 67.66 67.20 66.83 63.20 62.45 61.75 61.04 39.29 38.87 36.51 35.13 32.43 29.80 29.73 24.16 23.48 17.94 17.89
O
F
N
NH
CH
B (m)
67.66
A (q)
62.11
2.12
7.88
f1 (ppm)

Probe 14

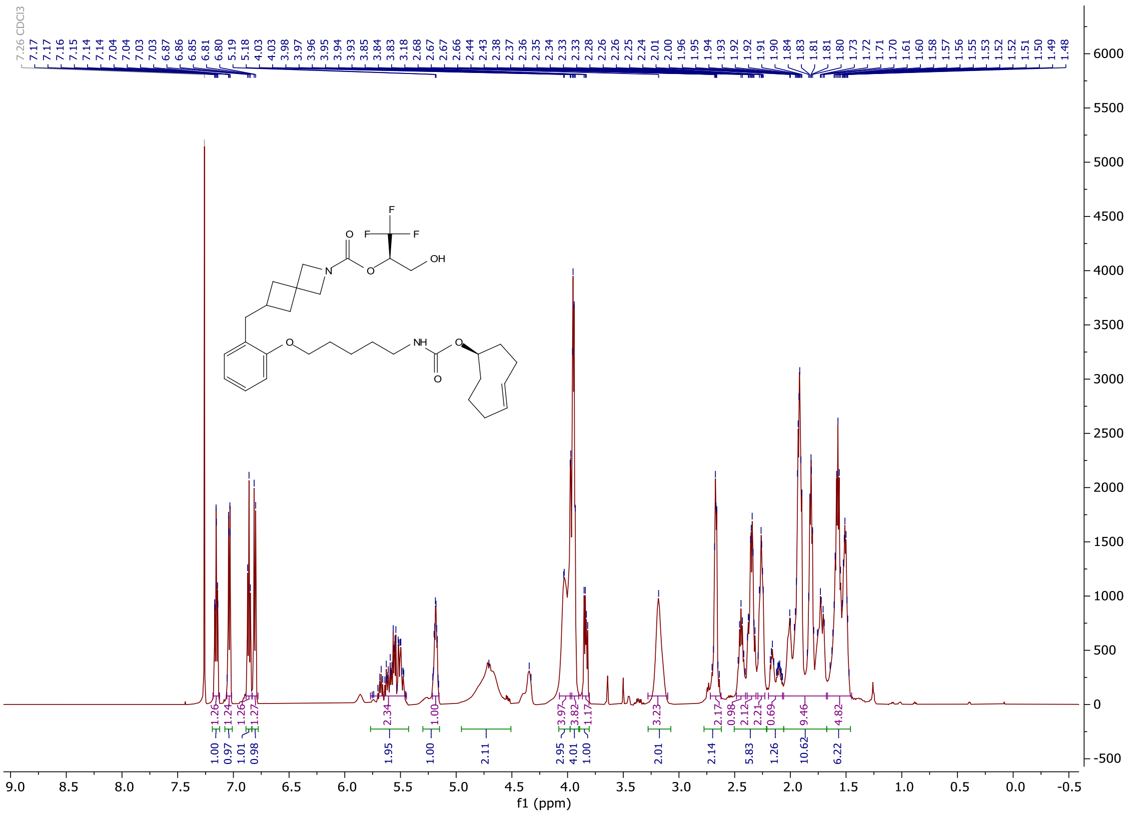
7.26 CDCl3
7.17 7.17 7.16 7.15 7.14 7.04 7.03 7.03 6.87 6.86 6.85 6.81 6.80 5.19 5.18 4.03 4.03 3.98 3.97 3.96 3.95 3.94 3.93 3.85 3.84 3.83 3.18 2.68 2.67 2.67 2.66 2.44 2.43 2.38 2.37 2.36 2.35 2.34 2.33 2.33 2.28 2.26 2.26 2.25 2.24 2.01 2.00 1.96 1.95 1.94 1.93 1.92 1.92 1.91 1.90 1.84 1.83 1.81 1.81 1.80 1.73 1.72 1.71 1.70 1.61 1.60 1.58 1.57 1.56 1.55 1.53 1.52 1.52 1.51 1.50 1.49 1.48
1.26 1.24 1.26 1.27 2.34 1.00 3.97 3.82 1.17 3.23 2.17 0.98 2.12 2.21 0.69 9.46 4.82
1.00 0.97 1.01 0.98 1.95 1.00 2.11 2.95 4.01 1.00 2.01 2.14 5.83 1.26 10.62 6.22
F
F
F
O
OH
N
O
O
NH
O
O
f1 (ppm)

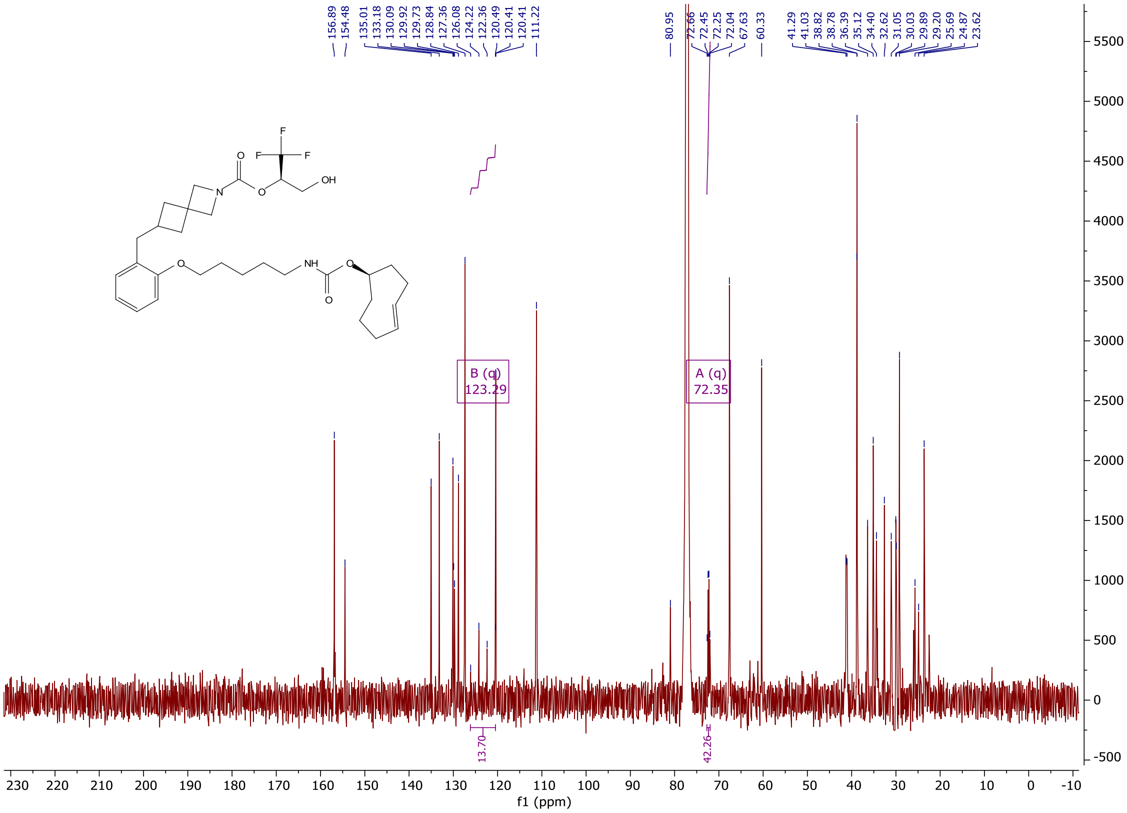
156.89 154.48 135.01 133.18 130.09 129.92 129.73 128.84 127.36 126.08 124.22 122.36 120.49 120.41 120.41 111.22 80.95 72.66 72.45 72.25 72.04 67.63 60.33 41.29 41.03 38.82 38.78 36.39 35.12 34.40 32.62 31.05 30.03 29.89 29.20 25.69 24.87 23.62
B (q)
123.29
A (q)
72.35
13.70
42.26
f1 (ppm)
